# A method for finding epistatic effects of maternal and fetal variants

**DOI:** 10.3389/fgene.2025.1420641

**Published:** 2025-03-31

**Authors:** Michael Nodzenski, Min Shi, David M. Umbach, Brian Kidd, Taylor Petty, Clarice R. Weinberg

**Affiliations:** ^1^ Biostatistics and Computational Biology Branch, National Institute of Environmental Health Sciences, Research Triangle Park, NC, United States; ^2^ Sciome LLC, Research Triangle Park, NC, United States

**Keywords:** epistasis, maternal effects, case-parent design, oral cleft, evolutionary algorithm

## Abstract

**Introduction:**

Pregnancy involves a double genome, and genetic variants in the mother and her fetus can act together to influence risk for pregnancy complications, adverse pregnancy outcomes, and diseases in the offspring. Large search spaces have hindered the discovery of sets of single nucleotide polymorphisms (SNPs) that act epistatically.

**Methods:**

Previously, we proposed a method for case-parent studies, called the Genetic Algorithm for Detecting Genetic Epistasis using Triads or Siblings (GADGETS), that can reveal autosomal epistatic SNP-sets in the child’s genome. Here we incorporate maternal SNPs, thereby extending GADGETS to nominate SNP-sets containing offspring loci only, maternal loci only, or both. We use a permutation procedure to impose a preference for epistatic over outcome-related but non-epistatic SNP sets. Our maternal-fetal extension uses case-complement-sibling pairs together with mother-father pairs, exploiting Mendelian transmission and a mating-symmetry assumption.

**Results:**

In simulations of 1,000 case-parents triads with 10,000 candidate SNPs, GADGETS successfully detected simulated multi-locus effects involving 3-5 SNPs but was somewhat less successful at distinguishing epistatic SNPs from sets of non-epistatic SNPs that each conferred high risk independently. Though the epistasis-mining algorithms MDR-PDT, TrioFS, and EPISFA-LD were originally designed to find epistatic offspring variants, we generalize them to include maternal SNPs and search more broadly. GADGETS outperformed those competitors and could successfully mine a much larger list of candidate SNPs. Applied to dbGaP data, GADGETS nominated several multi-SNP maternal-fetal sets as potentially-interacting risk factors for orofacial clefting.

**Discussion:**

The extended version of GADGETS can mine for epistasis that involves maternal SNPs.

## 1 Introduction

For health conditions with “complex” etiologies, risk is influenced by both genetic and non-genetic factors. An interesting possibility, particularly for conditions with onset early in life, is that the prenatal environment plays a role. That intrauterine exposures can impact the health of the developing fetus is well established; for example, fetal alcohol syndrome can include brain damage, characteristic modifications in facial features, and growth deficits (May et al., 2018).

The interplay of the mother’s genotype and that of her fetus can also affect the gestation. For example, in pregnancies affected by Rh incompatibility, in which the mother’s blood is Rh-negative and the fetus is Rh-positive, the mother produces antibodies that attack fetal red blood cells (Urbaniak and Greiss, 2000). Aside from the immediate danger posed to the fetus, epidemiological evidence suggests that related mechanisms may predispose the child to schizophrenia later in life (Hollister et al., 1996; Cannon et al., 2002). Indeed, SNPs in the gene that encodes Rh factor in both mother and child genotypes appear to jointly influence the child’s risk of schizophrenia (Palmer et al., 2002).

Three types of mother-genotype/child-disease associations have been investigated: maternally-mediated genetic effects, maternal-fetal interactions, and “imprinting.” This paper proposes methods for detecting the first two of these. Maternally-mediated genetic effects arise when the maternal genotype, possibly acting through the intrauterine environment, affects the child’s disease risk, regardless of the child’s genotype. Maternal-fetal interactions, in contrast, arise when the maternal and child genotypes synergistically affect the child’s disease risk. The genetic loci involved may be the same in mother and child, but need not be. Some maternal-fetal interactions involving the same locus in both genomes are called “maternal-fetal genotype incompatibilities” (Sinsheimer et al., 2003; Childs et al., 2011). We consider here only autosomal genetic effects.

Studies of maternally-mediated effects and maternal-fetal interactions can effectively use a case-parents triad design, in which individuals with the condition of interest and their biological parents (regardless of parental case status) are genotyped for the autosome. In addition, the triad design can be used to study conditions that affect the mother or mother-child unit, like preeclampsia or premature rupture of the membrane during pregnancy. Weinberg, Wilcox and Lie (1998) described a log-linear approach for estimating a maternal SNP’s effect based on the idea that, under a mating-symmetry assumption, risk-related maternal variants should be more common in the mother than in the father. Sinsheimer, Palmer and Woodward (2003) extended the log-linear approach to model maternal-fetal incompatibilities, and further extensions accommodate a variety of additional data types and assumptions (Kraft et al., 2004; Kraft et al., 2005; Minassian et al., 2005; Hsieh et al., 2006; Childs et al., 2010; Childs et al., 2011). Cordell, Barratt and Clayton (2004) described a related method based on conditional logistic regression that could model either maternally-mediated effects or maternal-fetal interactions.

These methods evaluate one or a few pre-specified loci. None were designed to mine for higher-order maternally-mediated effects or maternal-fetal interactions among a large collection of candidate maternal and child SNPs. Identifying multi-SNP interactions involves search spaces that are usually too large to examine all possible combinations of SNPs, even among relatively few candidates. For example, among 10,000 candidate SNPs, the number of four-SNP sets exceeds 10^14^.

Existing algorithms designed to mine for epistatic genetic interactions using triad data include GADGETS (Nodzenski et al., 2022), MDR-PDT (Martin et al., 2006), TrioFS (Schwender et al., 2011), and EPISFA-LD (Xiang et al., 2020). None can search genome-wide, and they differ substantially in the number of candidate SNPs they can accommodate. While GADGETS can mine 10,000 candidates, the others generally allow only several hundred (Nodzenski et al., 2022).

Our primary goal is to demonstrate that, by including maternal SNPs as candidates, GADGETS’s search capabilities expand to include both epistatic multi-SNP maternally-mediated effects and maternal-fetal interactions. Further, we show that the same tactic of including maternal SNPs also expands the capabilities of MDR-PDT, TrioFS, and EPISFA-LD.

This paper is structured as follows. We begin by reviewing GADGETS’s current implementation and then describe modifications needed to incorporate maternal variants. We discuss how those modifications impact previously developed permutation-based inferential procedures and graphical visualization techniques. Next, we carry out simulations to assess GADGETS’s ability to detect epistatic multi-SNP maternally-mediated effects or maternal-fetal interactions by searching among 10,000 candidate SNPs. In scenarios with two nonoverlapping epistatic SNP-sets, we attempt to identify both. In scenarios that have an epistatic SNP-set together with a group of non-epistatic risk-related SNPs, we attempt to isolate the epistatic SNP-set. We then describe how to adapt MDR-PDT, TrioFS, and EPISFA-LD to search for interactions involving maternal SNPs and compare their performance with GADGETS’s using 25–500 candidate SNPs. Finally, we apply our extended version of GADGETS to candidate SNPs from a case-parents GWAS of orofacial clefting (Beaty et al., 2010) to nominate sets of maternal and fetal variants as possibly jointly risk-associated. Throughout, we assume a case-parents design in which the child has the condition under study; however, our approach also applies to triads ascertained because the mother or mother-child unit suffered a shared condition, such as a pregnancy complication.

## 2 Materials and methods

### 2.1 Review of GADGETS’s original implementation

GADGETS (Nodzenski et al., 2022) is an epistasis-mining algorithm that takes as input a collection of autosomal candidate SNPs and searches for SNP-sets that exhibit preferential joint transmission to affected offspring, giving evidence that they are synergistically related to the disease. GADGETS can broadly be conceptualized as a two-stage process: 1) a search to nominate risk-relevant SNP-sets, and 2) post-processing the nominated SNP-sets to filter out non-epistatic ones. Its search strategy employs a computational optimization technique known as a “genetic algorithm,” also known as an “evolutionary algorithm,” which mimics Darwinian evolution through natural selection in an artificial population of SNP-sets (Holland, 1975). Post-processing includes a permutation procedure and selection and weighting for visualization.

#### 2.1.1 Genetic algorithm

We assume a case-parent triad design and a list of candidate autosomal SNPs. GADGETS’s genetic algorithm optimizes a fitness function that maps a SNP-set, set of d SNPs, to a fitness score. The fitness score is designed to be larger for SNP-sets with stronger evidence for joint transmission to affected offspring. For a fixed SNP-set size d , the genetic algorithm searches for high fitness SNP-sets by 1) generating an initial population of SNP-sets by randomly sampling from the list of candidate SNPs; 2) assigning a fitness score to each SNP-set; and 3) passing that population through a series of generations in which high fitness SNP-sets are more likely to propagate to the next-generation. At each generation, diversity is enhanced through operations inspired by biological mutation and crossover. To enable a broad search capable of recovering multiple risk-related SNP-sets, we simulate this evolution-inspired process separately within each of a thousand distinctly evolving island populations (Andre and Koza, 1996), with occasional cross-migration among islands within specified four-island clusters. This island structure enables the analyst to exploit parallel computing. Because the SNP-set size d is constant across generations, we carry out separate runs of GADGETS across a range of values for *d*, from two to five in this paper. At convergence, results for each SNP-set size are aggregated across islands and stored for integrated analysis.

Each SNP-set’s fitness score for GADGETS is based on the paired genotype difference vectors from every triad. Genotypes are coded as 0, 1, or 2, representing copies of an analyst-designated allele (usually the minor allele, but the choice does not affect results). For the $i^{\text{th}}$ triad, the difference vector, $x_{i}$ , is computed as $x_{i}=D_{i}-C_{i}$ , where $D_{i}$ is a vector of d elements containing allele counts for each SNP in that SNP-set for the $i^{\text{th}}$ case, and $C_{i}$ is the corresponding vector of untransmitted parental alleles, comprising the complement-sibling alleles. (We assume Mendelian transmission, so none of the d SNPs is related to fetal survival.) Then, under the null hypothesis that none of the SNPs is related to disease, the $i^{\text{th}}$ case would be equally likely to inherit either allele from each parent. Consequently, under a general no-effect null, each element of $x_{i}$ has expectation zero.

This fitness score relies on the intuition that, if all SNPs in a SNP-set act jointly, then those alleles should be inherited together by cases more frequently than by their paired complement-siblings. Consequently, across families, the associated difference vectors should systematically differ from the zero vector and tend to lie in the same orthant. The fitness score quantifies how well a SNP-set aligns with that expectation by using a quadratic form that is similar to a paired Hotelling’s T^2^ statistic.

To prioritize detecting SNP-sets with epistatic effects compared to those where each SNP can influence risk even without the others present (i.e., a set of SNPs with separate, noninteracting effects), GADGETS uses several tactics. First, the fitness score gives more weight to families whose difference vectors are long, because they should be more informative about epistasis. Second, GADGETS makes data-driven decisions about which allele at each locus, and in the context of a particular SNP-set, to designate a provisional risk allele and whether its mode of inheritance in that set appears to be recessive. Finally, GADGETS shrinks the length of the weighted mean difference vector because, for truly epistatic SNP-sets, a case should carry provisional risk alleles at all *d* loci in a SNP-set more often than does their complement sibling. To further prioritize SNPs with joint super-multiplicative effects, we set the paired-difference covariances to 0 for the difference vector’s variance for SNPs in the set that are not in linkage, e.g., those on different chromosomes. Let ${\bar{x}}_{w}$ be the resulting shrunken weighted mean genotype difference vector, w be the sum of the weights, and $\hat{\Sigma }$ be the corresponding weighted covariance matrix. The fitness score $\left(S\right)$ , is $S=w{\bar{x}}_{w}^{T}{\hat{\Sigma }}^{-1}{\bar{x}}_{w}$ (Nodzenski et al., 2022).

#### 2.1.2 Permutation-based inference

We previously introduced (Nodzenski et al., 2022) an epistasis test that is applicable to individual SNP-sets and assesses whether the data suggest that the set’s component SNPs contribute super-multiplicatively to disease risk. This test helps eliminate non-epistatic SNP-sets from the sets nominated by the genetic algorithm and aids visualization of interacting SNP-sets via network plots. The permutations replicate the null scenario in which the disease risk is attributable to a specific SNP-set only due to multiplicative marginal effects across the constituent unlinked SNPs, which contrasts with the alternative in which there are epistatic effects involving the genetically unlinked elements of the SNP-set. Any SNPs in the SNP-set considered linked are treated as a single locus in the permutations. First, at each component locus in the SNP-set, the genotypes for the mother, father, and case are kept linked together. Then permutation-based pseudo-families are formed by randomly reassembling those fragmented triads (Supplementary Figure S1). Absent population stratification, each such pseudo-family should have been equally likely to be sampled as an observed family under a no-epistasis null hypothesis. This permutation procedure preserves the marginal effects for each unlinked component locus but destroys super-multiplicative joint effects by ensuring that unlinked sets of SNPs occur together independently within the reassembled pseudo-families. The epistasis test p-value compares the fitness-score for the SNP-set from the actual families to the distribution of fitness scores from these permutation-based pseudo-families. We note however that the resulting p-value is strictly valid as a p-value only when the data used for testing are independent from those used to nominate the specific SNP-set, e.g., when applied to a separate data set or enough families have been studied to allow a hold-out set. Because our simulations and our data application use the same data for both, we use the term “h-value” to remind us of that limitation; nevertheless, smaller “h-values” suggest epistasis. We use h-values based on 10,000 sets of randomly re-assembled case-parent pseudo-families.

#### 2.1.3 Visualization

To visualize results for a particular dataset, simulated or real, GADGETS aggregates evidence across runs with different SNP-set sizes into a single network plot (Nodzenski et al., 2022). After the GA evolution has converged for the island populations, graphical scores are assigned to individual SNPs and, separately, to pairs of SNPs, such that higher scores reflect stronger evidence that a particular SNP or SNP pair participates in risk-related epistasis. The graphical scores rely on a subset of the SNP-sets nominated by the genetic algorithm together with h-values from the epistasis permutation test for those SNP-sets. Nodzenski et al. (2022) proposed restricting attention to the top 10 highest-fitness SNP-sets for each d . Then single SNP scores and paired-SNP scores are computed based on the h-values for the SNP-sets they are part of. In the resulting network plot, a larger, darker node (representing a SNP) reflects a larger SNP-specific score, while a thicker, darker connection (edge) between nodes corresponds to a larger pair score.

### 2.2 Proposed modifications to include maternal SNPs

Extending GADGETS to search for epistatic maternally-mediated effects or maternal-fetal interactions requires revising the list of candidate SNPs, the fitness score, permutation testing, and visualization. Otherwise, GADGETS operates as detailed in Nodzenski et al. (2022). In particular, we used the default values for all tuning parameters as specified in that paper.

#### 2.2.1 List of candidate SNPs

If GADGETS is provided a list of candidates that includes autosomal maternal loci in addition to autosomal child loci, the extended algorithm can stochastically consider SNP-sets comprising child SNPs only, maternal SNPs only, or a mix. To include maternal SNPs, we use fathers as their paired controls, under an assumption of mating symmetry in the study’s source population. Under that assumption, mother-father pairs with genotypes (*g*_*1*_ *, g*_*2*_) are as likely as are those with genotypes (*g*_*2*_ *, g*_*1*_), for any genotypes *g*_*1*_ and *g*_*2*_. (This assumption is weaker than random mating and does not require absence of population stratification.) If, conditional on the affected child’s genotype, those parental SNPs are unrelated to the child’s risk of disease, there should also be mating symmetry among the studied families.

The list of candidate SNPs that is input into GADGETS must now designate maternal versus offspring loci. In our simulations we always include both the maternal and child SNP at each given candidate locus, so that, for *p* candidate loci, GADGETS would be provided 2*p* candidate SNPs. This mirrored inclusion of the same candidate maternal and child loci is not required. Although maternal effects have mostly not been directly studied, variants previously identified by case-control studies, *e.g.*, in GWAS, but that are actually only related to risk when the mother carries them, could often nevertheless have produced observable case/control differences through those maternal effects, simply because more case mothers would have carried those variants and passed them on. This argues for mirrored inclusion of the same SNPs in the fetus and in the mother, allowing them to compete without prejudice in the stochastic search.

**FIGURE 1:**
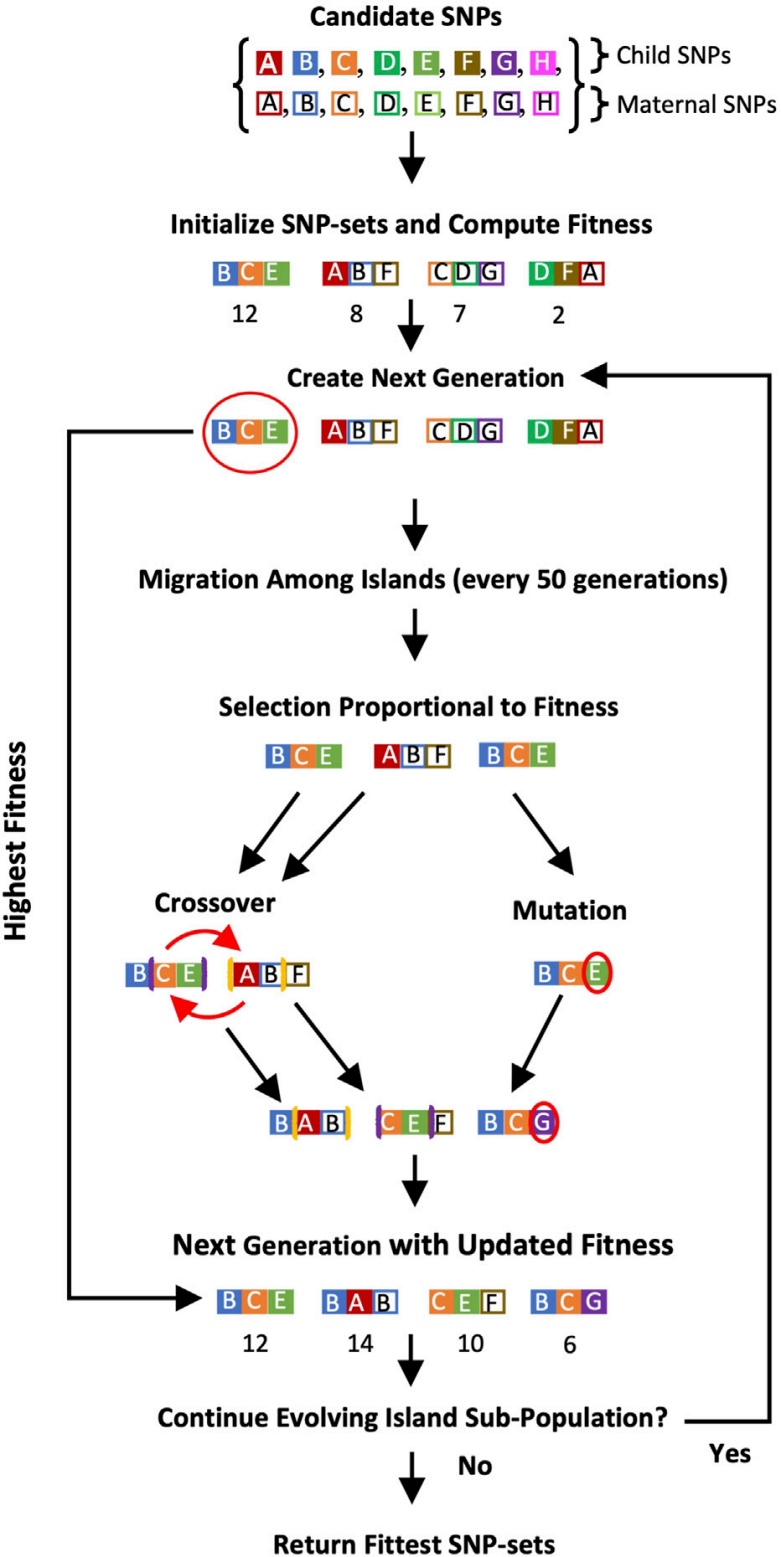
Flowchart of the GADGETS algorithm for a single island subpopulation. In this schematic, we include four SNP-sets of size d=3 . Details about the island model and migration among islands are in Nodzenski et al. (2022).

A schematic showing GADGETS’s algorithm for combined maternal and fetal candidate SNPs appears in Figure 1.

#### 2.2.2 Fitness score

For a revised fitness score, if a SNP-set’s $j^{th}$ element is a maternal SNP, then the $j^{th}$ element of that family’s genotype difference vector is the difference in allele count between the mother and father. We provide details regarding computation of the covariance matrix, $\hat{\Sigma }$ , and carrying out mutations (Supplementary Methods S1).

For SNP-sets that include only child SNPs, this fitness score is unmodified and should again reflect evidence of epistasis (Nodzenski et al., 2022). For sets containing only maternal SNPs, under mating symmetry, maternal SNPs that jointly increase risk, *i.e.,* those with maternally-mediated epistatic effects, should be jointly carried more frequently by mothers of cases than by their fathers. That asymmetry will also occur if risk is enhanced by a maternal fetal interaction, such as the maternal-fetal incompatibility of the Rh example in the Introduction. For example, in affected triads one allele could be systematically absent in the mother but systematically present in the fetus. Then, the evident maternal risk allele and the evident fetal risk allele will be the two different alleles at that locus and that maternal and fetal configuration of alleles will tend to co-occur in affected triads. Elements of ${\bar{x}}_{w}$ that correspond to maternal SNPs should therefore be large in magnitude only when the SNPs are risk-related through maternal effects or maternal-fetal interactions or incompatibilities.

#### 2.2.3 Permutation-based inference

The h-value for the permutation-based epistasis test is computed analogously in that we again create permuted data sets of reassembled pseudo-families based on unlinked loci, and then compare the observed fitness score to those based on permuted data. For a SNP-set containing only child loci or one containing only maternal loci, the process is exactly as before except for genotypic source. For a SNP-set containing both maternal and child loci, we consider a mother-child pair of loci from the same chromosome as non-separable, as if linked, and permute them as a unit. This precaution prevents the generation of Mendelian incompatibilities within the resulting pseudo-families. A small h-value again reflects evidence for multi-locus epistasis involving only child SNPs, only maternal SNPs, or a combination (Supplementary Methods S2).

With a SNP-set composed of both maternal and child SNPs, the epistasis test does not focus on maternal-fetal interaction as a separate alternative; its permutations are designed to preserve only marginal effects by individual loci, not to preserve epistasis between loci within either the maternal subset or the child subset. To provide an h-value that instead specifically reflects evidence for joint involvement of both maternal and child SNPs, we can employ the same general approach as the epistasis test, but we now jointly permute all maternal SNPs as a unit, and, separately, all child SNPs (Supplementary Figure S2). These permutations allow marginal SNP effects and/or epistasis within the maternal and child subsets but destroy any joint maternal-fetal effects. This test should only be done when, within the SNP-set, no child locus is on the same chromosome as any maternal locus.

### 2.3 Simulations

We simulated multi-SNP maternally-mediated effects and maternal-fetal effects in triad data using the TriadSim R package (Shi et al., 2018), slightly modifying the software to allow risk to depend on both fetal and maternal SNPs but otherwise retaining default settings. TriadSim uses actual parent data but removes any systematic differences between parental autosomal genotypes by interchanging them at random and then selects offspring to have the condition where selection probabilities depend on genotype according to a user-specified model for risk. To simulate triad data for a scenario with an epistatic SNP-set of a pre-specified size, the user specifies a baseline risk and the risk associated with the SNP-set. We used a logistic model to simulate dominant genetic interactions with a super-multiplicative effect of jointly carrying at least one copy of each variant in the SNP-set. Some scenarios also include non-epistatic SNPs that increase risk through a jointly multiplicative effect, *i.e.*, contributing just main effects in the logistic model. To simulate realistic linkage disequilibrium structure, the software uses actual data from dbGaP as a template; we used data from chromosomes 10–13 from a GWAS of orofacial clefting (Beaty et al., 2010), restricted to families from Asian populations. SNPs designated as risk-related were spread among these four chromosomes (Supplementary Material). We used candidate SNP lists that were modestly pruned for linkage disequilbrium (LD) (all pairwise $R^{2}<0.8$ ) and minor allele frequency (>0.01). Each simulated data set contained 1,000 families and 5,000 distinct genetic loci, 1,250 from each chromosome. The datasets input into GADGETS thus included a total of 10,000 input candidate SNPs (5,000 child and 5,000 maternal).

We simulated data for 1,000 families from 24 different scenarios (Supplementary Table S1). To see how stable the results would be across multiple simulated datasets from the same scenario, we simulated each scenario ten times. Twelve scenarios included just one risk-related SNP-set: two with an epistatic maternally-mediated effect; and ten with a maternal-fetal interaction. Eight scenarios each included two risk-related SNP-sets: five with both being maternal epistatic or both fetal or both maternal-fetal, and three in which the effects were of two distinct types (*e.g.,* one maternal-fetal SNP-set, one maternal-only SNP-set). We also included three scenarios with one epistatic SNP-set together with four or five nonepistatic risk-related SNPs. Regardless of scenario, the epistatic SNP-sets always included between three and five SNPs. Finally, we included a scenario without any epistasis, with four child and four maternal independently risk-related SNPs. Scenarios that included non-epistatic risk-related SNPs allowed us to probe how well GADGETS could distinguish epistatic from non-epistatic (log-additive) multi-SNP effects.

For visualization, we used the 10 SNP-sets with the highest fitness scores for each d to construct graphical scores.

### 2.4 Comparison with competing methods

Although MDR-PDT, TrioFS, and EPISFA-LD were not originally designed to consider maternal SNPs, we modified them by using the same approach that we implemented to extend GADGETS. Since these methods are all fundamentally based on comparisons of case genotypes to control genotypes, we input parental and fetal genetic data so that maternal SNPs were treated like additional case SNPs and paternal SNPs as their corresponding control SNPs.

We compared GADGETS’s performance in discovering SNP-sets with epistatic maternal-fetal or maternal-only effects to the performance of these competitors using modified versions of simulation Scenarios 1, 2, 11, and 12. We still based these simulations on 1,000 families but scaled back the number of candidate SNPs from 10,000 to 24, 100, or 500 to accommodate limitations among the competitors. The list of candidate SNPs now included 12, 50, and 250 distinct genetic loci (each specified once as maternal and once as child), comprising the three SNPs with a simulated epistatic effect alongside SNPs randomly sampled from the remaining 4,997 simulated loci.

GADGETS, MDR-PDT and TrioFS require the analyst to pre-specify a size for the SNP-sets considered ( d in GADGETS, though GADGETS later combines the evidence from several choices). We specified the correct value (*i.e.,* 3) so that each could nominate the exact risk-related SNP-set. GADGETS and MDR-PDT nominate SNP-sets only of that size, TrioFS nominates SNP-sets up to that size. EPISFA-LD does not enforce a SNP-set size and can return sets containing any number of SNPs. Though an appealing feature in real applications, it complicates these comparisons because EPISFA-LD and TrioFS have larger implicit search spaces than MDR-PDT and GADGETS. To ensure comparable computational resources, we ran each method on a single processor. Other computational tuning parameters matched those reported for Scenario 2 in Nodzenski et al. (2022).

### 2.5 Application

We used GADGETS to investigate whether genetic epistasis involving maternal SNPs may be related to risk of cleft lip, with or without cleft palate (CL/P). We selected candidate SNPs from a GWAS of orofacial clefting (Beaty et al., 2010) and analyzed families of European and Asian ancestry separately. (These data are publicly available through dbGaP.) Because any SNP that participates in an epistatic SNP-set should have been somewhat over-transmitted to cases and thereby exhibit a small induced marginal effect, nominating candidate SNPs from from previous GWAS results when searching for epistatis is a principled way to keep the size of the candidate pool manageable. Consequently, we included the candidate SNPs curated by (Li et al., 2015), chosen based on marginal associations in children or based on having a role in the Wnt signaling pathway, and we separately supplemented that list with loci that showed evidence for maternal-effects (p ≤ 5 x 10^−4^ in either Asian or European ancestry groups) based on results from Shi et al. (2012). In Asians, we analyzed 1,312 total candidate SNPs (656 each for mothers and children) across 886 families. In Europeans, we analyzed 1,408 total SNPs (704 each for mothers and children) across 652 families.

## 3 Results

### 3.1 Recovery of simulated epistatic SNP-sets by GADGETS

Across the 24 simulation scenarios, GADGETS often recovered SNP-sets involved in fetal-only epistasis, maternal-only epistasis, and multi-SNP maternal-fetal interactions. For the ten scenarios involving a single SNP-set with maternal-fetal interaction (Scenarios 1–10), when d was equal to the true number of risk-related SNPs, the top-scoring SNP-set was the risk-associated SNP-set for at least 80% of replicates in seven of the ten scenarios (Supplementary Table S2) and in 74 of the 100 total runs. For the two scenarios involving a single 3-SNP set associated with a maternal epistatic effect (Scenarios 11–12), when *d* was 3, the highest-fitness SNP-set was always the true risk-related one.

GADGETS also frequently recovered both risk-related SNP-sets in the eight scenarios that simulated two such SNP-sets (Supplementary Table S3). When *d* was 3, at least one of the two was always ranked among the top-two GADGETS-nominated SNP-sets for each replicate of Scenarios 13–20. Additionally, the top two GADGETS-nominated SNP-sets were the true risk-associated sets for at least five of ten replicates in seven of the eight scenarios. Likewise, GADGETS often recovered both risk-related SNP-sets when each had a different type of epistatic effect, *e.g.*, one maternal-fetal and the other maternal-only (Scenarios 17–19), or when both epistatic SNP-sets involved only fetal SNPs (Scenario 20). Scenario 21 was epistasis-null, as it only included SNPs with individual effects on risk; with no truly epistatic SNP-sets present, GADGETS-nominated SNP-sets, regardless of size, always contained at least one, most often two of the SNPs that had individual effects. For Scenarios 22 and 23, where each had a 3-SNP epistatic set in competition with singletons with strong independent effects (relative risk near 2), when using the correct SNP-set size, GADGETS top-scoring nomination was the correct epistatic SNP-set in five and six of the ten replicate scenarios, respectively (Supplementary Table S2). Using larger SNP-set sizes, the top scoring SNP-set often contained the correct epistatic SNP-set as a subset. For Scenario 24, which had a 4-SNP epistatic set in competion with singletons, GADGETS using the correct SNP-set size nominated the correct set in seven out of ten replicates.

#### 3.1.1 Simulated risk-related SNP-sets identified in network plots

**FIGURE 2:**
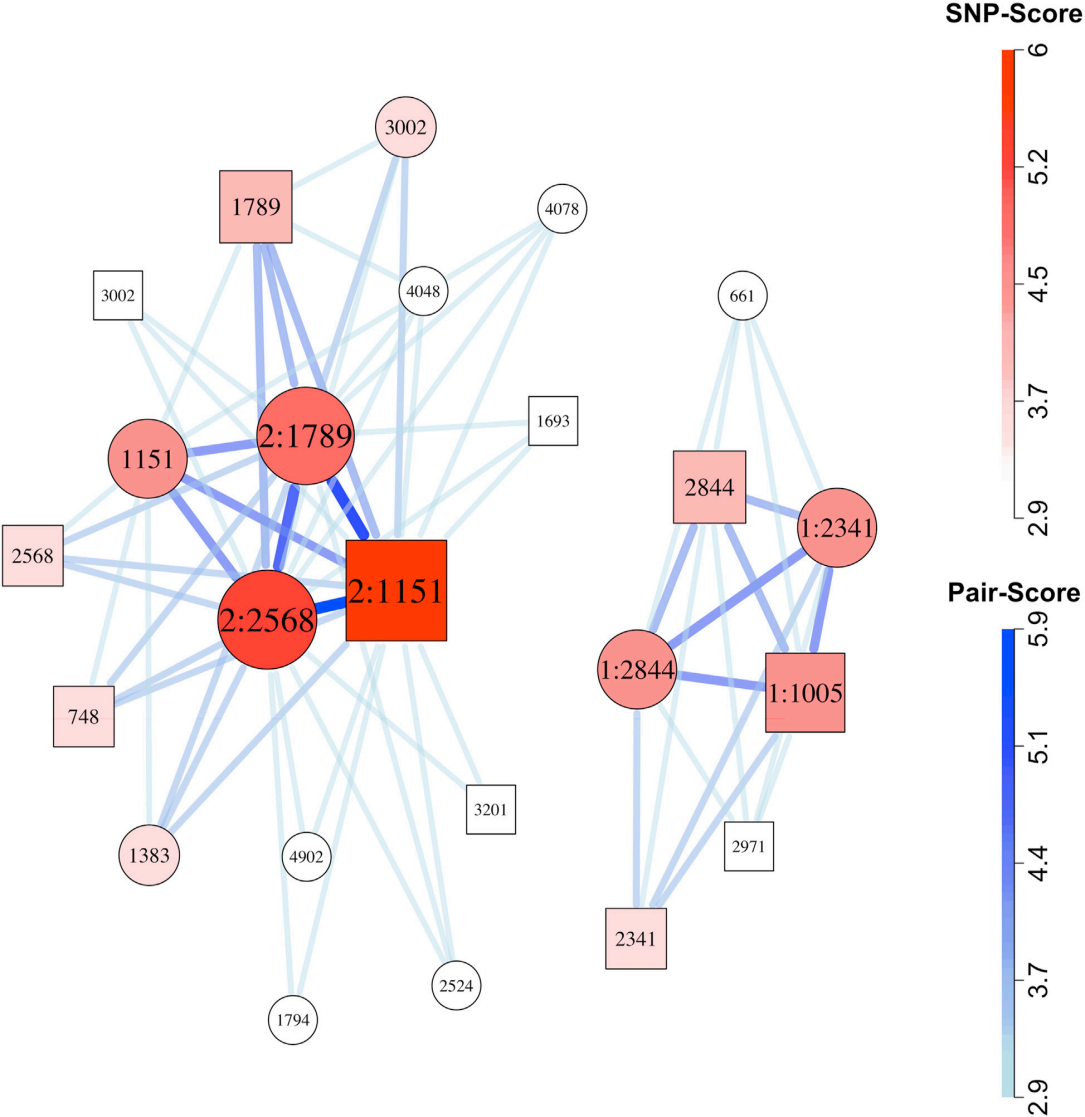
Network plot for simulation Scenario 13, replicate 10. Circles represent child SNPs and squares represent maternal SNPs. SNP label “1:” indicates membership in the first risk-related SNP-set, and label “2:” indicates membership in the second risk-related SNP-set. Both SNP-sets represent maternal-fetal interactions. A SNP with no colon in the label is not-risk related. The number following the colon is the simulated SNP’s identifier. Maternal and child SNPs with the same identifier represent the same locus. The SNP-sets that contributed to this plot were selected using the method described by Nodzenski et al. (2022). After applying that filter, a total of 67 SNP-pairs (comprising 24 SNPs) received graphical scores. We plotted all of those pairs. Thicker, darker connections indicate higher SNP-pair graphical scores; larger, darker vertices indicate higher individual SNP graphical scores.

We constructed network plots for one randomly selected replicate from each simulation scenario (Figure 2; Supplementary Figures S3–S25). Of the 12 network plots for scenarios with one simulated risk-related SNP-set and no singleton risk-related SNPs, all contained the full SNP-set. Of the eight network plots from simulation scenarios with two risk-related SNP-sets, seven of the eight plots included both SNP-sets, while the remaining plot (Supplementary Figure S21) included just one. For the three scenarios with one epistatic SNP-set plus a number of SNPs with strong but independent marginal effects, the epistatic SNP-set was often, but not always, found in the network plots from the ten replicates. The network plots for those scenarios tended to be cluttered, however, and the epistatic sets were sometimes hard to see distinctly, even when present. (e.g., Supplementary Figures S22–S25). For Scenario 21 with no epistasis (Supplementary Figure S22), weak visual evidence wrongly supported possible epistasis.

### 3.2 Recovery of risk-related epistatic SNP-sets by competing methods

By augmenting case and complement-sibling genotypes with maternal and paternal genotypes as if they respectively represented cases and matched controls, GADGETS, MDR-PDT, TrioFS and EPISFA-LD all recovered risk-related SNP sets with higher-order maternal-fetal interaction effects (Table 1; Supplementary Table S4). GADGETS tended to run the fastest and was also most able to identify the risk-related SNP-sets, particularly when searching at least 100 candidate SNPs. It always nominated the full set of risk-related SNPs as its highest ranked SNP set (Table 1). For 100 candidate SNPs, GADGETS often ran faster than any competitor, while, for 500 candidates, it always ran substantially faster than the others.

**TABLE 1:** Comparison of GADGETS with competitors in finding a simulated maternal-fetal genetic interaction over a range of input SNP numbers. The simulation scenario is a modified version of Scenario 2; it involves a single risk-related SNP-set with one maternal and two child SNPs. Due to limitations in the number of input SNPs that competitors could analyze with reasonable run times, the number of candidate SNPs was at most 500. For each replicate, half of the input SNPs are child SNPs, and the remainder are the corresponding maternal SNPs. Run times are hours:minutes:seconds.

Input SNPs	GADGETS		MDR-PDT		TrioFS		EPISFA-LD	
	Max risk SNPs found (Rank)^a^	Run time	Max risk SNPs found (Rank)^a^	Run time	Max risk SNPs found (Rank)^a^	Run time	Max risk SNPs found (Rank)^a^ ^b^	Run time
Replicate 1								
24	3(1)	00:01:25	2(1)	00:00:04	3(1)	00:41:51	3[3](1)	00:00:26
100	3(1)	00:01:27	2(1)	00:04:15	2(1)	00:44:13	3[2](Top 3)	00:01:25
500	3(1)	00:07:12	0	09:46:19	0	03:26:35	**	**
Replicate 2								
24	3(1)	00:01:25	2(1)	00:00:04	3(1)	00:43:59	3[1](Top 2)	00:00:31
100	3(1)	00:01:29	2(6)	00:04:43	2(6)	00:43:49	3[0](Top 2)	00:01:47
500	3(1)	00:07:13	2(4)	08:59:16	2(4)	03:38:14	**	**
Replicate 3								
24	3(1)	00:01:24	3(1)	00:00:03	3(1)	00:44:06	3[0](1)	00:00:34
100	3(1)	00:01:30	3(1)	00:05:08	2(1)	00:43:56	3[0](1)	00:02:05
500	3(1)	00:07:17	3(1)	08:58:14	0	03:39:13	**	**
Replicate 4								
24	3(1)	00:01:30	1(1)	00:00:04	3(1)	00:42:11	3[1](1)	00:00:44
100	3(1)	00:01:30	2(3)	00:05:26	1(1)	00:44:14	3[0](Top 2)	00:02:42
500	3(1)	00:08:04	0	10:16:28	0	03:37:44	**	**
Replicate 5								
24	3(1)	00:01:29	2(1)	00:00:04	3(1)	00:41:33	3[2](1)	00:01:10
100	3(1)	00:01:31	2(1)	00:04:48	3(3)	00:49:13	3[2](1)	00:02:50
500	3(1)	00:07:13	2(1)	09:51:38	2(1)	03:26:55	**	**
Replicate 6								
24	3(1)	00:01:24	2(1)	00:00:04	3(1)	00:51:51	3[2](Top 2)	00:00:32
100	3(1)	00:01:36	2(1)	00:04:59	2(1)	00:43:36	3[1](Top 2)	00:03:46
500	3(1)	00:07:10	2(1)	09:51:53	1(1)	03:38:11	**	**
Replicate 7								
24	3(1)	00:01:26	2(1)	00:00:04	3(1)	00:48:11	3[1](Top 2)	00:00:33
100	3(1)	00:01:32	2(1)	00:05:22	3(1)	00:50:16	3[1](Top 2)	00:02:06
500	3(1)	00:07:10	2(1)	09:52:40	2(1)	03:36:06	**	**
Replicate 8								
24	3(1)	00:01:26	2(1)	00:00:05	3(1)	00:48:44	3[0](Top 2)	00:01:55
100	3(1)	00:01:33	2(1)	00:05:08	2(1)	00:50:48	3[0](Top 3)	00:03:52
500	3(1)	00:07:08	2(1)	09:51:46	2(9)	03:55:48	**	**
Replicate 9								
24	3(1)	00:01:24	2(1)	00:00:05	3(1)	00:48:52	3[2](Top 2)	00:00:35
100	3(1)	00:01:31	2(7)	00:05:37	2(1)	00:50:50	3[1](Top 3)	00:01:50
500	3(1)	00:07:03	2(1)	09:49:02	0	03:40:23	**	**
Replicate 10								
24	3(1)	00:01:28	2(1)	00:00:05	3(2)	00:50:46	3[0](Top 2)	00:00:48
100	3(1)	00:01:34	1(10)	00:05:22	0	00:41:50	3[0](Top 2)	00:02:00
500	3(1)	00:08:07	0	08:59:23	0	03:53:49	**	**

^a^ Maximum number of SNPs, contained in the risk-related SNP-set, in any single SNP-set/model among the 10 highest ranking SNP-sets/models, and corresponding SNP-set/model rank (1 = highest). Zero is reported when models failed to identify any risk-related SNPs. ** is reported when the software was unable to output results.

^b^ Square brackets indicate the number of non-risk-related SNPs, returned by EPISFA-LD; it can return sets of any size, rather than a pre-specified size of interest.

Although GADGETS’ performance was better, MDR-PDT, TrioFS, and EPISFA-LD performed reasonably well in finding maternal-fetal interactions. Regardless of scenario, when considering 100 or fewer candidates, EPISFA-LD always returned all three risk-related SNPs, although it usually nominated at least one (and sometimes several) SNP that was not risk-related along with the risk-related ones. It was unable to output results for 500 candidate SNPs. Even for 500 candidates, MDR-PDT frequently nominated at least two risk-related SNPs among its top ten ranked models, sometimes as its highest ranked model; long run-times were a limitation. TrioFS often recovered at least two, and sometimes all three, risk-related SNPs among its top models, even when considering 500 candidate SNPs.

Results were similar when searching for epistatic maternally-mediated effects (Supplementary Tables S5, S6).

### 3.3 Runtimes for competing methods

We used a high-performance computing cluster that allowed up to 135 simultaneous single core jobs per user (NVIDIA V100 Tensor Core GPU, 16G – 32G memory). Making use of those distributed computing resources, when d ranged from 2 to 5, for 1,000 families and 10,000 candidate SNPs, GADGETS typically took 10–15 min to run. Precise timings for jobs on single cores that did not use distributed computing, considering 1,000 families, 25-500 candidate SNPs, and d=3 , are reported in Table 1, and Supplementary Tables S4–S6.

### 3.4 Application

**FIGURE 3:**
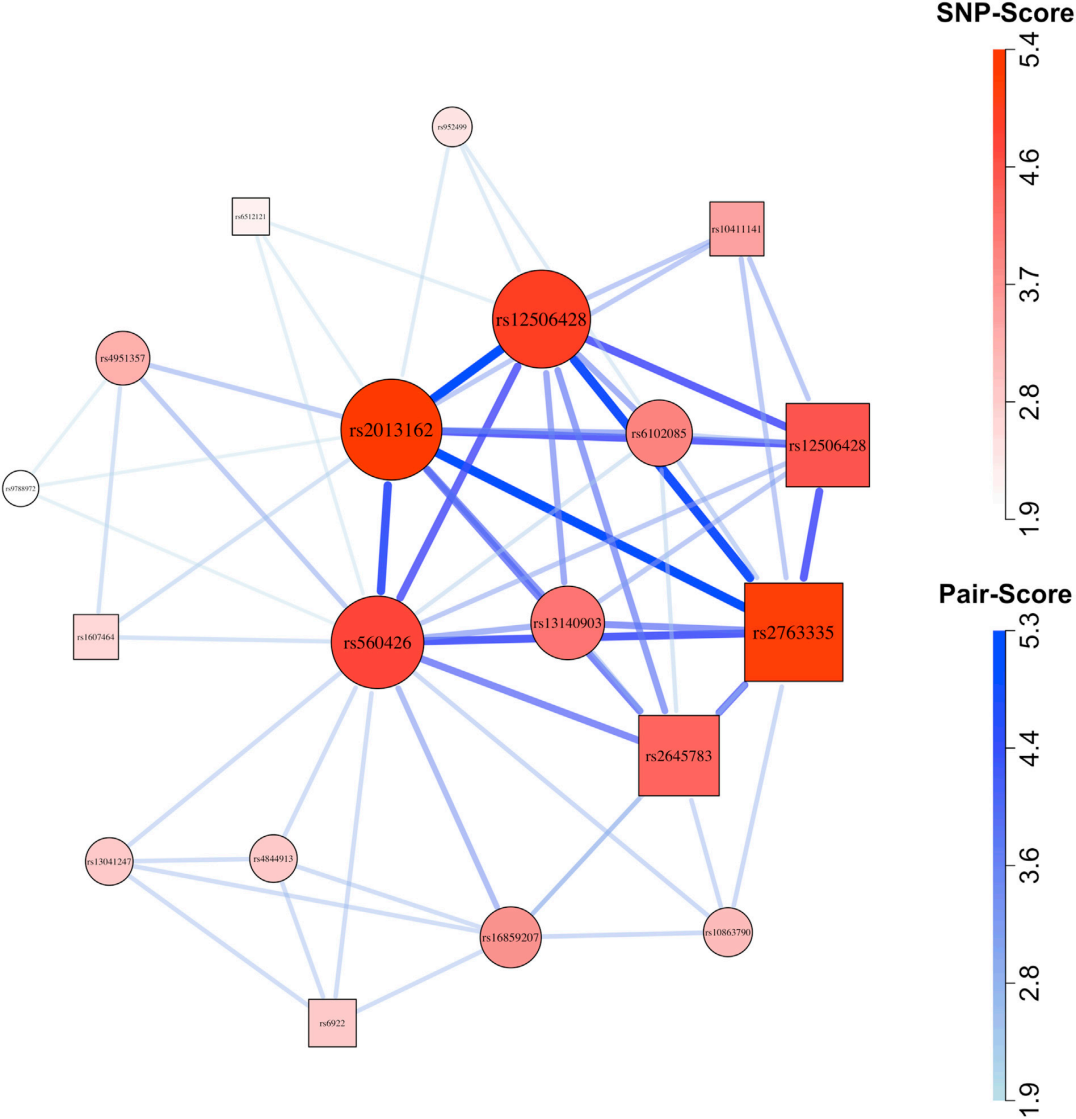
Network plot for Asian (cleft lip with or without cleft palate) case-parent triad data. Circles represent child SNPs and squares represent maternal SNPs. The SNP-sets that contributed to this plot were selected using the method described by Nodzenski et al. (2022). After applying that filter, we plotted all 60 SNP-pairs (comprising 19 SNPs) that received graphical scores. Thicker, darker connections indicate higher SNP-pair graphical scores; larger, darker vertices indicate higher individual SNP graphical scores.

**FIGURE 4:**
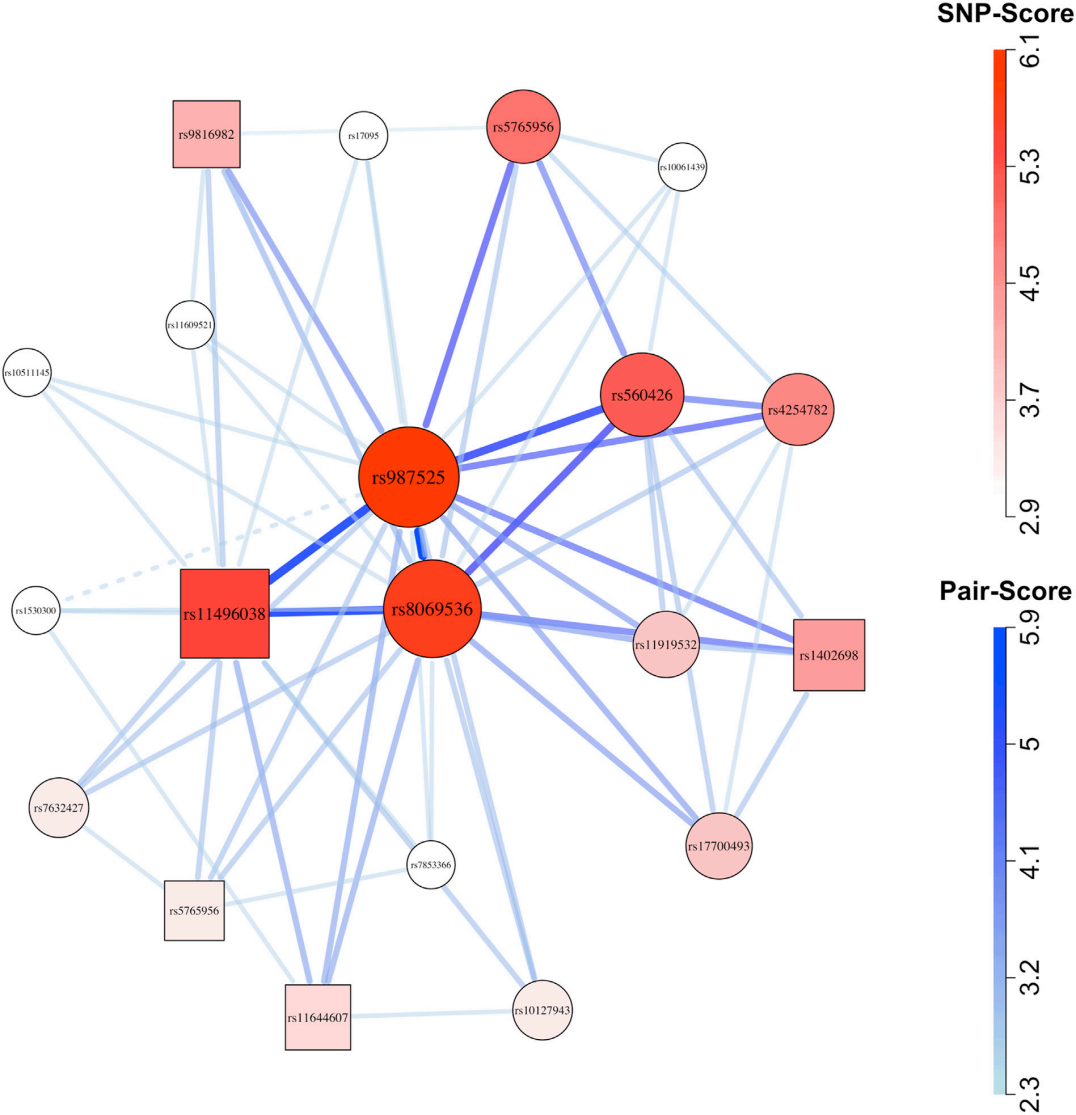
Network plot for European (cleft lip with or without cleft palate) case-parent triad data. Circles represent child SNPs and squares represent maternal SNPs. The SNP-sets that contributed to this plot were selected using the method described by Nodzenski et al. (2022). After applying that filter, we plotted all 65 SNP-pairs (comprising 20 SNPs) that received graphical scores. Thicker, darker connections indicate higher SNP-pair graphical scores; larger, darker vertices indicate higher individual SNP graphical scores.

GADGETS network plots suggested possible maternal-fetal interactions in both ancestry groups. In Asians (Figure 3), the highest-fitness SNP-set of size d=5 (child rs560426, child rs2013162, child rs12506428, child rs13140903, and maternal rs2763335) appeared prominently. The epistasis test indicated evidence for genetic interaction among those SNPs (h = 0.0001), and the maternal-fetal interaction test specifically suggested maternal-fetal effects (h = 0.0001), with the joint risk genotype carried by 48 mother/case pairs versus 4 father/complement-sibling pairs. In Europeans (Figure 4), the second-ranked SNP-set of size three (maternal rs11496038, child rs987525, and child rs8069536) appeared prominently in the network. The h-value for epistasis was small (h = 0.0011) and so was the maternal-fetal interaction h-value (h = 0.009), with the joint risk genotype carried by 53 mother/case pairs versus 7 father/complement-sibling pairs. Similarly, the third ranked SNP-set of size four (maternal rs1402698, child rs11919532, child rs987525, and child rs8069536) appeared near the center of the plot, and both h-values were small (epistasis h = 0.0001, maternal-fetal h = 0.0041, with the joint risk genotype carried by 45 mother/case pairs versus 3 father/complement-sibling pairs).

## 4 Discussion

Across 24 diverse simulation scenarios, we showed that, by treating mothers as cases and fathers as controls, GADGETS could reliably search 10,000 candidate SNPs to detect epistatic maternal effects and/or maternal-fetal interactions involving 2-5 total SNPs, while also retaining its previously demonstrated ability to detect epistasis among child SNPs. In scenarios with two simulated risk-related SNP sets, regardless of whether the effect was epistatic among inherited SNPs, epistatic maternally-mediated, or maternal-fetal interaction, GADGETS frequently identified both. GADGETS was somewhat less successful at distinguishing simulated supermultiplicative epistatic effects from multi-SNP independent multiplicative effects, and often nominated single SNPs along with epistatic sets.

Our simulated data was based on epistatic risk scenarios in which the epistasis was fairly strong, and the algorithm’s performance will not always be as good. Our goal here was to establish that the algorithm can work to nominate epistatic sets of variants. For it to succeed, at least part of an epistatic SNP set must have been included in the list of candidates employed. Careful attention to nomination of SNPs for consideration would exploit prior evidence of case/control differences either for mothers or for offspring and/or evidence based on transmission distortion. Typically such prior evidence would be marginal (per SNP) and not based on epistasis. One good source of SNP candidates would be SNPs that have been found to be predictive in a polygenic risk score. The user might want to screen out SNPs that are found to be rare in the case families, as such SNPs would likely not be involved in epistasis that could explain a substantial fraction of the cases. The user might also want to restrict pairwise LD for SNPs included in the list. We do not recommend GADGETS for studying haplotypes. Biological reasons for including variants with known relevant functional effects could also be important.

We regard GADGETS as a promising tool for uncovering epistatic SNP-sets, but we see it as a tool more for exploration and data mining than for inference. Our simulations involved 1,000 case-parent triads, a larger number than many studies would have available. We did not examine GADGETS’s performance across a range of study sizes, but note that the search space is huge and even strong epistasis involving low-frequency variants will be difficult to find without a large number of families. If one has later access to an independent set of case-parent triads from the same population, then permutation tests can provide valid p values for confirming epistasis.

When applied to triads affected by CL/P, GADGETS nominated SNP-sets with plausible maternal-fetal interactions. Statistical evidence for maternal-fetal genetic interaction was provided by low h-values combined with indicators of large joint effects, *i.e*., many more mother/case pairs than father/complement-sibling pairs carried nominated SNP-sets. From a biological standpoint, some maternal SNPs in those SNP-sets were biologically plausible as contributing to a suboptimal gestation. For example, in Europeans, the third-ranked SNP-set of size d=4 contained a maternal SNP (rs1402698) in the *GRB14* gene, a gene whose expression is related to glucose homeostasis (Ding et al., 2020). Supporting a role for glucose metabolism, epidemiological studies have associated maternal diabetes with a higher risk of orofacial clefts (Spilson et al., 2001; Correa et al., 2008; Tinker et al., 2020). Taken together, these findings suggest that maternal SNP rs1402698 could, through its relationship to glucose control in mothers, influence the child’s risk of clefting.

In Asians, some of the maternal SNPs in SNP-sets with high fitness scores also appeared to have plausible biological links to palate defects. In particular, the top-ranked SNP-set of size d=5 contained maternal SNP rs2763335, which is located in the *COL13A1* gene. Mutations in that gene can cause congenital myasthenic syndromes, neurological conditions that are often accompanied by a high-arched palate (Logan et al., 2015; Dusl et al., 2019; Rodriguez Cruz et al., 2019).

Although the performance of GADGETS was our main focus, an interesting secondary finding was that MDR-PDT, TrioFS, and EPISFA-LD can be adapted to detect maternal-fetal or epistatic maternally-mediated effects with the same data extension that we used for GADGETS. Although, in these single core, nonparallel runs, GADGETS typically ran faster and more completely identified the sets of epistatic SNPs, efforts to scale up those competing methods to accommodate more candidate SNPs and to optimize for interactions that involve maternal variants may be worthwhile.

We did not consider the possible role of imprinting, where implications for risk depend on the parent of origin. Cordell, Barratt and Clayton (2004) noted that power to distinguish between imprinting and maternal-fetal effects may be limited in analyses based on complement-sibling controls. We anticipate that that may be true for GADGETS. For example, if a maternally-inherited copy of an allele (but not a paternally-inherited copy) confers increased risk for the child, then mothers of cases would carry that allele more often than fathers, and cases would jointly carry the allele more often than would the complement-siblings. To GADGETS, such a SNP-set could spuriously appear to be a maternal-fetal interaction. In practice, detailed follow-up analysis, based on inferring (based on nearby SNPs) the parent-of-origin for that SNP for all families where the case and both parents were all heterozygous, is therefore required to carefully consider the inheritance patterns of SNP-sets nominated by GADGETS.

We also did not consider the possibly interactive role of a maternal exposure such as smoking, or a persistent maternal phenotype like high BMI or diabetes. Such considerations are important and are the subject of our ongoing work.

In addition to conditions like birth defects that only affect offspring, GADGETS could be used to study the joint effects of maternal and fetal genomes on conditions that affect the mother only or the mother-child unit, such as pregnancy complications, by regarding the mother or mother-child unit as the “case”. For example, studies have used a triad design to investigate the role of genetics in preeclampsia (Jääskeläinen et al., 2016; Luo et al., 2018). GADGETS could be applied directly for such studies.

We did not address how to accommodate families where one of the parents was not genotyped. While GADGETS can accommodate sporadic missing genotypes (the family will be considered uninformative for the corresponding SNP), a family in which the genotypes from one parent are completely missing must be excluded in the current implementation. In actual studies, fathers are often unavailable, but one could potentially include such families by genotyping unaffected siblings and using that sibling information to do multiple imputation of the paternal genotypes. Studies might also want to consider interactions involving environmental exposures or heterogeneity across outcome sub-phenotypes. We are currently investigating extensions applicable to those questions.

We noted an interesting phenomenon with our visualization method. While network plots often displayed the simulated epistatic sets prominently, we saw a tendency for maternal SNPs corresponding to an offspring-based epistastic SNP-set or for offspring SNPs corresponding to a maternally-mediated epistatic SNP-set to appear in the network plot, albeit less prominently. This phenomenon arises because, for any SNP-set, large family weights can induce a high fitness score when a subset of the component SNPs have epistatic effects. In GADGETS, SNP-sets that contain maternal and offspring SNPs from the same locus tend to be assigned large family weights, even when the epistasis involves only the maternal SNPs or only the offspring SNPs.

In summary, GADGETS can detect autosomal epistatic maternal and maternal-fetal effects in triad studies by treating mothers as cases and fathers as their paired controls under an assumption of mating symmetry in the source population. Though GADGETS does not yet scale up to genome-wide, it can accommodate substantially more candidate SNPs than competitors, and good candidates are often available from previous GWAS. GADGETS should be a useful tool in helping researchers characterize more fully the joint roles of maternal and fetal genetic variants in the development of complex young-onset diseases and pregnancy complications.

## Data Availability

The simulated data used to evaluate our proposed method are publicly available. The simulated data for Scenarios 1-20 can be found at: https://figshare.com/articles/dataset/simulated_data_tar_gz/22041074/1?file=39120551. The simulated data for Scenarios 21-24 can be found at: https://figshare.com/articles/dataset/simulateddata_21to24_tar_gz/25460671/1. The data used for the clefting examples can be obtained from dbGAP at: https://www.ncbi.nlm.nih.gov/projects/gap/cgi-bin/study.cgi?study_id=phs000774.v1.p1.
