# Supplementary material for: A method for finding epistatic effects of maternal and fetal variants
Source: Front Genet. 2025 Mar 31;16:1420641. doi: 10.3389/fgene.2025.1420641 (PMC11995191; doi:10.3389/fgene.2025.1420641)

Supplementary Material

A method for finding epistatic effects of maternal and fetal variants

Michael Nodzenski,^1^ Min Shi,^1^ David M. Umbach,^1^ Brian Kidd, ^2^ Taylor Petty, ^2^ Clarice R. Weinberg*^1^

^1^Biostatistics and Computational Biology Branch, National Institute of Environmental Health Sciences, NIH, Research Triangle Park, NC 27709, USA

^2^Sciome LLC, 2 Davis Drive, Research Triangle Park, NC 27709, USA

***Correspondence:** Clarice R. Weinberg: weinberg@niehs.nih.gov

# Supplementary Methods

**1.1 Modifications to GADGETS’s algorithm to accommodate maternal SNPs**

In addition to the modifications described in the main text, two other points warrant clarification regarding GADGETS’s operations when maternal SNPs are included among the input candidates.

First, we point out a detail about the calculation of the covariance matrix, $\hat{\Sigma}$. When two loci in a SNP-set are regarded as genetically unlinked (*e.g.*, on different chromosomes), GADGETS sets the corresponding element of $\hat{\Sigma}$ to zero. When a SNP-set contains both child and maternal loci from the same biological chromosome, even though they represent separate individuals, we do not set their covariance to zero.

Second, for maternal candidate SNPs eligible to enter the algorithm via mutation, pre-computed sampling probabilities are based on marginal effects estimated using conditional logistic regression (Nodzenski, et al., 2022), except substituting maternal and paternal genotypes for the case and complement-sibling genotypes, respectively.

**1.2 Interpretation of h-values from the permutation-based epistasis test**

Interpretation of h-values from the permutation-based epistasis test depends on the makeup of the specific SNP-set to which the procedure is applied. For a child-only SNP-set, the h-value assesses evidence for epistasis involving at least two SNPs. For a maternal-only SNP-set, the h-value quantifies evidence of an epistatic maternally-mediated effect involving at least two maternal SNPs. For a mixed SNP-set with both maternal and child SNPs, interpretation is less straightforward and depends on the assumed linkage structure of the component SNPs.

With these mixed SNP-sets, we use the term ‘linkage’ somewhat loosely. We regard a set of SNPs as ‘in linkage’ if they are not assumed to be independently inherited under the no-epistatic-effects null. The assumption encompasses the usual concept for loci located near each other on the same physical chromosome where, for convenience, we operationalize ‘near each other’ as ‘on the same chromosome’ – though other choices are possible. We extend the concept, however, to regard a locus in the offspring and a locus in the mother, both on the same nominal chromosome, as in linkage - despite their being in different individuals. In our epistasis test, SNPs assumed ‘in linkage’ are permuted as a unit, whereas those assumed to be unlinked are shuffled independently. Our definition of linkage for maternal and offspring SNPs prevents any permuted pseudo-families from violating Mendelian inheritance.

Recognizing how linked loci are permuted is key to interpreting h-values. If a SNP-set contains SNPs that are assumed to be linked, the marginal *and* joint effects of those linked SNPs in the observed data are preserved in the permuted data. Consequently, the h-value will not reflect any joint effects among linked SNPs. Among unlinked SNPs or groups of SNPs, however, the permutation procedure will destroy synergistic effects, so that a low h-value provides evidence for epistasis among those unlinked units.

In general, the h-value for a SNP-set that includes both maternal and fetal SNPs does not necessarily reflect maternal-fetal interaction; instead, it assesses non-specific multi-locus genetic effects. Consider a SNP-set of two child SNPs and two maternal SNPs and assume they are on four different chromosomes. A low h-value might reflect epistasis involving only the two fetal SNPs, an epistatic maternally-mediated effect involving only the two maternal SNPs, a maternal-fetal interaction involving one child SNP and one maternal SNP, or a higher-order synergism among maternal and child SNPs. Thus, the h-value could be impacted by any combination of child-SNP epistasis, epistatic maternally-mediated effects, or maternal-fetal interactions, and can only be interpreted as quantifying non-specific evidence for genetic interaction.

In special cases, however, an h-value for a mixed SNP-set must assess maternal-fetal interaction. Consider, for example, a SNP-set comprising two maternal SNPs and two child SNPs, with both maternal SNPs linked and both child SNPs linked but with the maternal and child SNPs on different chromosomes. More generally, if analysts are interested in examining evidence specifically for maternal-fetal interactions, we recommend computing the maternal-fetal interaction h-value, as described in the main text.

**1.3 Selection of risk-related SNPs in simulations**

We selected risk-related SNPs for epistatic SNP-sets or for singletons from the list of candidate SNPs on chromosomes 10-13. For a candidate SNP to be eligible to be risk-related, a SNP first had to meet the minor allele frequency requirement appropriate for the given scenario (Supplementary Table S1); thus, eligible SNPs were a subset, delineated by minor allele frequency, of the candidate SNPs.

For scenarios that had a single risk-related SNP-set and contained only mother or only child SNPs, the SNPs in the set were sampled at random from the eligible SNPs, one per chromosome, moving through the chromosomes in numerical order, stopping when the 3-SNP or 4-SNP set was filled. If a scenario involved single SNP-set of size three or four that contained a mix of mother and child SNPs with all mother and child SNPs from different loci, all the maternal SNPs were sampled first, followed by the child SNPs. In the scenarios with risk-related SNP-set of size five, one of the chromosomes would necessarily contain two SNPs. In those scenarios, the risk-related SNP-set had either two maternal and three child SNPs or three maternal and two maternal SNPs. When a risk-related SNP-set of size five had two maternal SNPs, those were sampled at random from the eligible SNPs on chromosome 10, and the remaining three SNPs (child) were each sampled, one per chromosome, from chromosomes 11, 12, and 13. When a risk-related SNP-set of size five had two child SNPs, those were sampled at random from the eligible SNPs on chromosome 13, and the remaining three SNPS (maternal) were each sampled, one per chromosome, from chromosomes 10, 11, and 12. For scenarios involving epistatic maternal and child SNPs at the same locus, sampling was done as described above for the appropriate number of maternal SNPs and loci were duplicated as child SNPs.

In scenarios with two risk-related SNP-sets, the first SNP-set was sampled following the rules describe above, the SNPs selected into that SNP-set were removed from the list of eligible SNPs, and the second risk-related SNP-set again used the same rules applied to the reduced list of eligible SNPs.

In scenarios 21-24 that involved multiple singleton risk-related SNPs, possibly in addition to an epistatic SNP-set, the epistatic SNP-set was sampled first (if needed) following the rules described above and its SNPs were removed from the set of eligible SNPs. Singleton risk SNPs were sampled at random, one per chromosome, cycling from chromosome 10 through chromosome 13, beginning again with chromosome 10, and continuing until the number of singletons needed for the scenario was achieved.

# Supplementary Tables and Figures

## Supplementary Tables

**Supplementary Table S1.** Simulation Scenarios

|  | Number of | | |  |  |  |  |
| --- | --- | --- | --- | --- | --- | --- | --- |
| Simulation scenario | Risk-related SNP-sets | Maternal SNPs in SNP-set^a^ | Child SNPs in SNP-set^a^ | Same maternal and child SNPs^b^ | Risk with all interacting SNPs^c^ | Minor allele frequency^d^ | Number of cases (controls) carrying epistatic risk-related SNP-set^e^ |
| 1 | 1 | 1 | 2 | No | 40/1000 | 0.075-0.085 | 64(0); 69(1); 89(4); 87(7); 94(4); 94(3); 73(5); 83(2); 80(1); 85(7) |
| 2 | 1 | 1 | 2 | No | 30/1000 | 0.075-0.085 | 61(5); 60(2); 63(4); 51(5); 75(6); 64(4); 74(4); 62(3); 71(2); 53(4) |
| 3 | 1 | 2 | 1 | No | 40/1000 | 0.075-0.085 | 83(5); 63(12); 72(5); 81(7); 80(6); 81(5); 74(4); 91(3); 95(6); 91(9) |
| 4 | 1 | 2 | 1 | No | 30/1000 | 0.075-0.085 | 84(6); 74(1); 67(4); 58(5); 72(4); 72(4); 71(3); 61(2); 67(1); 71(2) |
| 5 | 1 | 2 | 2 | No | 100/1000 | 0.095-0.105 | 75(0); 71(2); 76(1); 83(1); 92(2); 77(2); 60(2); 69(3); 60(3); 67(0) |
| 6 | 1 | 2 | 2 | No | 75/1000 | 0.095-0.105 | 51(1); 54(2); 51(1); 36(1); 46(0); 57(0); 55(1); 60(0); 50(2); 64(0) |
| 7 | 1 | 2 | 2 | Yes | 20/1000 | 0.075-0.085 | 65(8); 88(6); 92(11); 103(15); 76(6); 71(10); 81(6); 76(6); 71(12); 78(12) |
| 8 | 1 | 2 | 2 | Yes | 15/1000 | 0.075-0.085 | 65(9); 62(11); 62(11); 61(13); 68(5); 67(10); 51(6); 66(13); 71(13); 66(6) |
| 9 | 1 | 3 | 2 | No | 300/1000 | 0.110-0.120 | 76(0); 73(0); 92(0); 95(0); 85(4); 76(2); 77(1); 89(1); 94(0); 101(0) |
| 10 | 1 | 3 | 2 | No | 250/1000 | 0.110-0.120 | 62(0); 74(0); 53(0); 59(0); 57(0); 68(1); 56(0); 70(2); 54(0); 57(0) |
| 11 | 1 | 3 | 0 | NA | 40/1000 | 0.075-0.085 | 70(2); 72(1); 88(6); 68(4); 75(5); 69(4); 76(1); 79(2); 76(3); 71(6) |
| 12 | 1 | 3 | 0 | NA | 30/1000 | 0.075-0.085 | 73(0); 59(8); 58(4); 73(3); 59(1); 55(3); 63(2); 52(6); 64(3); 56(5) |
| 13 | 2 | 1, 1 | 2, 2 | No | 40/1000 | 0.075-0.085 | 79(2), 74(6); 85(1), 73(4); 93(4), 76(6); 86(1), 77(2); 87(2), 65(5);  80(5), 71(5); 66(4), 89(1); 86(2), 78(3); 74(5), 76(7); 80(6), 73(4) |
| 14 | 2 | 1, 1 | 2, 2 | No | 30/1000 | 0.075-0.085 | 55(4), 67(2); 66(4), 69(3); 64(2), 62(3); 61(3), 68(6); 74(2), 59(5);  71(2), 69(3); 61(4), 60(6); 59(4), 68(6); 72(2), 58(7); 64(5), 56(5) |
| 15 | 2 | 3, 3 | 0, 0 | NA | 40/1000 | 0.075-0.085 | 86(5), 86(7); 71(2), 68(3); 83(2), 66(4); 74(7), 81(2); 57(5), 67(1);  88(4), 78(7); 87(3), 69(6); 98(2), 87(2); 71(0), 73(8); 79(6), 67(2) |
| 16 | 2 | 3, 3 | 0, 0 | NA | 30/1000 | 0.075-0.085 | 71(2), 59(8); 80(3), 63(4); 56(4), 56(2); 72(6), 69(4); 95(5), 64(8);  56(3), 63(3); 61(4), 48(5); 64(1), 74(4); 59(5), 53(4); 68(3), 67(2) |
| 17 | 2 | 1, 3 | 2, 0 | No | 40/1000 | 0.075-0.085 | 75(7), 81(2); 76(2), 73(5); 71(4), 79(1); 68(3), 73(1); 82(5), 77(4);  76(3), 66(6); 69(4), 75(8); 62(5), 81(2); 81(5), 84(5); 89(4), 69(9) |
| 18 | 2 | 1, 0 | 2, 3 | No | 40/1000 | 0.075-0.085 | 75(5), 69(4); 77(3), 81(6); 82(4), 75(2); 84(2), 76(2); 80(4), 69(1);  76(5), 73(3); 77(7), 69(2); 90(6), 67(3); 78(4), 71(3); 78(1), 80(3) |
| 19 | 2 | 3, 0 | 0, 3 | NA | 40/1000 | 0.075-0.085 | 78(2), 71(8); 75(4), 89(3); 75(3), 75(2); 67(5), 75(1); 77(1), 69(2);  76(5), 68(5); 68(4), 76(5); 77(2), 74(7); 86(3), 69(3); 69(5), 77(2) |
| 20 | 2 | 0, 0 | 3, 3 | NA | 40/1000 | 0.075-0.085 | 94(5), 74(6); 78(7), 78(9); 79(8), 75(4); 83(2), 67(4); 80(4), 91(5);  84(4), 79(2); 83(3), 96(4); 83(5), 80(4); 97(4), 75(4); 75(8), 77(5) |
| 21 | 8 | 1,1,1,1,  0,0,0,0 | 0,0,0,0,  1,1,1,1 | NA | 3.02/1000 | 0.070-0.080 | NA |
| 22 | 6 | 0,0,0,0,  1,1 | 3,1,1,1,  0,0 | NA | 40/1000; 3.33/1000 | 0.070-0.080 | 58(1); 73(5); 61(5); 67(3); 71(4); 64(4); 65(6); 54(4); 69(9); 72(4) |
| 23 | 6 | 3,1,1,1,  0,0 | 0,0,0,0,  1,1 | NA | 35/1000; 3.13/1000 | 0.080-0.090 | 79(3); 92(4); 73(4); 65(6); 53(3); 67(4); 85(4); 73(6); 68(2); 78(8) |
| 24 | 6 | 2,1,1,0,0 | 2,0,0,1,1 | No | 90/1000; 3.05/1000 | 0.100-0.110 | 56(6); 72(5); 67(1); 59(3); 68(2); 77(7); 61(2); 72(3); 70(2); 70(6) |

^a^ Values for the 10 replicates are separated by semi-colons. If a scenario involved two risk-related SNP-sets, the number of SNPs in each SNP-set are separated by a comma.

^b^ Risk-related genotypes for simulation scenarios with the same maternal and child SNPs are comprised of four total SNPs but only two distinct genetic loci. At each distinct genetic locus, both the mother and child must carry risk-related alleles.

^c^ The risk for those who do not carry a risk-related SNP-set genotype is 1.66/1000 for every scenario. In scenarios with two risk-related SNP-sets, the value reported is the risk associated with carrying either one of those SNP-sets, but not both. For people carrying both SNP-sets, risks combine multiplicatively, *i.e*., the log odds of risk will increase by twice the log odds ratio associated with carrying exactly one of the SNP-sets. In scenario 21, the number indicates risk associated with carrying any one of the eight single risk-related SNPs. For people carrying several of them, risks again combine multiplicatively (i.e., the log odds of risk when carrying *n* SNPs will increase by *n* times the log odds ratio of a single SNP). In scenarios 22-24, the first (larger) number is the risk associated with carrying the epistatic set of more than one SNP, and the second is the risk of carrying each individual single risk SNP, where risks combine multiplicatively as before for combinations.

^d^ Minor allele frequency among the parents of cases in the template data input into the simulation software.

^e^ Replicates for each simulation scenario are separated by semicolons. If more than one risk-related SNP-set was simulated, risk-related SNP-sets within a particular replicate are separated by a comma. For maternal-fetal interactions, the ‘control’ has a risk genotype when the father has a risk genotype at each risk-related maternal SNP and the complement-sibling has a risk genotype at each risk-related child SNP. For simulated maternal effects (scenarios 11 and 12), the ‘control’ has a risk genotype when the father has a risk genotype at each risk-related SNP. In scenarios 21-24, counts are given only for epistatic risk-related SNP- sets, not for single risk-related SNPs.

**Supplementary Table S2**. Recovery of simulated epistatic maternally-mediated effects and maternal-fetal interactions by GADGETS for simulations involving a single epistatic risk-related SNP-set. Entries are the proportion of replicates out of ten whose top ranked SNP-set contained the number of risk-related SNPs indicated by the column for the set-size ($d$) indicated by the row.

| Set-size (*d*) | ≥ 1 SNP | ≥ 2 SNPs | ≥ 3 SNPs | ≥ 4 SNPs | ≥ 5 SNPs |
| --- | --- | --- | --- | --- | --- |
| **Scenario 1 (maternal-fetal interaction with 3 risk-related SNPs)** | | | | | |
| 2 | 1.0 | 1.0 | - | - | - |
| 3 | 1.0 | 1.0 | 1.0 | - | - |
| 4 | 1.0 | 1.0 | 1.0 | - | - |
| 5 | 1.0 | 1.0 | 1.0 | - | - |
| **Scenario 2 (maternal-fetal interaction with 3 risk-related SNPs)** | | | | | |
| 2 | 0.7 | 0.7 | - | - | - |
| 3 | 0.8 | 0.8 | 0.8 | - | - |
| 4 | 0.8 | 0.8 | 0.8 | - | - |
| 5 | 0.8 | 0.8 | 0.8 | - | - |
| **Scenario 3 (maternal-fetal interaction with 3 risk-related SNPs)** | | | | | |
| 2 | 0.9 | 0.9 | - | - | - |
| 3 | 0.9 | 0.9 | 0.9 | - | - |
| 4 | 0.9 | 0.9 | 0.9 | - | - |
| 5 | 0.9 | 0.9 | 0.9 | - | - |
| **Scenario 4 (maternal-fetal interaction with 3 risk-related SNPs)** | | | | | |
| 2 | 0.9 | 0.9 | - | - | - |
| 3 | 0.9 | 0.9 | 0.9 | - | - |
| 4 | 0.9 | 0.9 | 0.9 | - | - |
| 5 | 1.0 | 1.0 | 1.0 | - | - |
| **Scenario 5 (maternal-fetal interaction with 4 risk-related SNPs)** | | | | | |
| 2 | 0.8 | 0.8 | - | - | - |
| 3 | 1.0 | 1.0 | 1.0 | - | - |
| 4 | 1.0 | 1.0 | 1.0 | 0.9 | - |
| 5 | 1.0 | 1.0 | 1.0 | 1.0 | - |
| **Scenario 6 (maternal-fetal interaction with 4 risk-related SNPs)** | | | | | |
| 2 | 0.3 | 0.2 | - | - | - |
| 3 | 0.8 | 0.8 | 0.7 | - | - |
| 4 | 0.9 | 0.9 | 0.8 | 0.7 | - |
| 5 | 1.0 | 1.0 | 1.0 | 0.9 | - |
| **Scenario 7 (maternal-fetal interaction with 4 risk-related SNPs)** | | | | | |
| 2 | 1.0 | 1.0 | - | - | - |
| 3 | 1.0 | 0.9 | 0.9 | - | - |
| 4 | 1.0 | 1.0 | 1.0 | 0.4 | - |
| 5 | 1.0 | 1.0 | 1.0 | 0.9 | - |
| **Scenario 8 (maternal-fetal interaction with 4 risk-related SNPs)** | | | | | |
| 2 | 0.5 | 0.5 | - | - | - |
| 3 | 0.7 | 0.7 | 0.4 | - | - |
| 4 | 0.9 | 0.8 | 0.6 | 0.1 | - |
| 5 | 0.9 | 0.9 | 0.7 | 0.6 | - |
| **Scenario 9 (maternal-fetal interaction with 5 risk-related SNPs)** | | | | | |
| 2 | 1.0 | 1.0 | - | - | - |
| 3 | 1.0 | 1.0 | 1.0 | - | - |
| 4 | 1.0 | 1.0 | 1.0 | 1.0 | - |
| 5 | 1.0 | 1.0 | 1.0 | 1.0 | 0.8 |
| **Scenario 10 (maternal-fetal interaction with 5 risk-related SNPs)** | | | | | |
| 2 | 0.6 | 0.4 | - | - | - |
| 3 | 0.9 | 0.9 | 0.8 | - | - |
| 4 | 0.9 | 0.9 | 0.9 | 0.9 | - |
| 5 | 1.0 | 1.0 | 1.0 | 1.0 | 0.9 |
| **Scenario 11 (epistatic maternally-mediated effect with 3 risk-related SNPs)** | | | | | |
| 2 | 1.0 | 1.0 | - | - | - |
| 3 | 1.0 | 1.0 | 1.0 | - | - |
| 4 | 1.0 | 1.0 | 1.0 | - | - |
| 5 | 1.0 | 1.0 | 1.0 | - | - |
| **Scenario 12 (epistatic maternally-mediated effect with 3 risk-related SNPs)** | | | | | |
| 2 | 1.0 | 1.0 | - | - | - |
| 3 | 1.0 | 1.0 | 1.0 | - | - |
| 4 | 1.0 | 1.0 | 1.0 | - | - |
| 5 | 1.0 | 1.0 | 1.0 | - | - |
| **Scenario 22 (epistatic child effect with 3 risk-related SNPs amidst 5 non-epistatic individual SNPs)** | | | | | |
| 2 | 0.0 | 0.0 | - | - | - |
| 3 | 0.5 | 0.5 | 0.5 | - | - |
| 4 | 0.8 | 0.8 | 0.8 | - | - |
| 5 | 0.8 | 0.8 | 0.8 | - | - |
| **Scenario 23 (epistatic maternal effect with 3 risk-related SNPs amidst 5 non-epistatic individual SNPs)** | | | | | |
| 2 | 0.5 | 0.5 | - | - | - |
| 3 | 0.6 | 0.6 | 0.6 | - | - |
| 4 | 0.8 | 0.8 | 0.8 | - | - |
| 5 | 0.8 | 0.8 | 0.8 | - | - |
| **Scenario 24 (maternal-fetal interaction with 4 risk-related SNPs (2 child and 2 maternal) amidst 4 non-epistatic individual SNPs)** | | | | | |
| 2 | 0.0 | 0.0 | - | - | - |
| 3 | 0.6 | 0.6 | 0.6 | - | - |
| 4 | 0.7 | 0.7 | 0.7 | 0.7 | - |
| 5 | 0.9 | 0.9 | 0.9 | 0.9 | - |

**Supplementary Table S3.** Recovery of simulated epistatic maternally-mediated effects, maternal-fetal interactions, and epistatic child-SNP interactions by GADGETS for simulations involving two 3-SNP risk-related SNP-sets. Entries are the proportion of replicates out of ten whose first or second ranked SNP-set contained the number of risk-related SNPs indicated by the column for the set-size ($d$) indicated by the row.

| Set-size (*d*) | Risk-related SNP-set 1 | | | Risk-related SNP-set 2 | | | Total Risk-related SNPs | | | | | |
| --- | --- | --- | --- | --- | --- | --- | --- | --- | --- | --- | --- | --- |
|  | ≥ 1 SNP | ≥ 2 SNPs | 3 SNPs | ≥ 1 SNP | ≥ 2 SNPs | 3 SNPs | ≥ 1 SNP | ≥ 2 SNPs | ≥ 3 SNPs | ≥ 4 SNPs | ≥ 5 SNPs | 6 SNPs |
| **Scenario 13 (two SNP-sets with maternal-fetal interactions)** | | | | | | | | | | | | |
| 2 | 0.8 | 0.8 | - | 0.6 | 0.6 | - | 1.0 | 1.0 | 0.4 | 0.4 | - | - |
| 3 | 0.9 | 0.9 | 0.9 | 0.6 | 0.6 | 0.6 | 1.0 | 1.0 | 1.0 | 0.5 | 0.5 | 0.5 |
| 4 | 0.8 | 0.8 | 0.8 | 0.3 | 0.3 | 0.3 | 1.0 | 1.0 | 1.0 | 0.1 | 0.1 | 0.1 |
| 5 | 0.7 | 0.7 | 0.7 | 0.3 | 0.3 | 0.3 | 1.0 | 1.0 | 1.0 | 0.0 | 0.0 | 0.0 |
| **Scenario 14 (two SNP-sets with maternal-fetal interactions)** | | | | | | | | | | | | |
| 2 | 0.7 | 0.7 | - | 0.5 | 0.5 | - | 1.0 | 1.0 | 0.2 | 0.2 | - | - |
| 3 | 0.9 | 0.9 | 0.9 | 0.6 | 0.6 | 0.6 | 1.0 | 1.0 | 1.0 | 0.5 | 0.5 | 0.5 |
| 4 | 0.5 | 0.5 | 0.5 | 0.5 | 0.5 | 0.5 | 1.0 | 1.0 | 1.0 | 0.0 | 0.0 | 0.0 |
| 5 | 0.5 | 0.5 | 0.5 | 0.5 | 0.5 | 0.5 | 1.0 | 1.0 | 1.0 | 0.0 | 0.0 | 0.0 |
| **Scenario 15 (two SNP-sets with epistatic maternally-mediated effects)** | | | | | | | | | | | | |
| 2 | 0.8 | 0.8 | - | 0.4 | 0.4 | - | 1.0 | 1.0 | 0.2 | 0.2 | - | - |
| 3 | 0.8 | 0.8 | 0.8 | 0.7 | 0.7 | 0.7 | 1.0 | 1.0 | 1.0 | 0.5 | 0.5 | 0.5 |
| 4 | 0.7 | 0.7 | 0.7 | 0.5 | 0.5 | 0.5 | 1.0 | 1.0 | 1.0 | 0.2 | 0.2 | 0.2 |
| 5 | 0.6 | 0.6 | 0.6 | 0.5 | 0.5 | 0.5 | 1.0 | 1.0 | 1.0 | 0.1 | 0.1 | 0.1 |
| **Scenario 16 (two SNP-sets with epistatic maternally-mediated effects)** | | | | | | | | | | | | |
| 2 | 0.8 | 0.8 | - | 0.3 | 0.3 | - | 1.0 | 1.0 | 0.1 | 0.1 | - | - |
| 3 | 1.0 | 1.0 | 1.0 | 0.3 | 0.3 | 0.3 | 1.0 | 1.0 | 1.0 | 0.3 | 0.3 | 0.3 |
| 4 | 0.8 | 0.8 | 0.8 | 0.2 | 0.2 | 0.2 | 1.0 | 1.0 | 1.0 | 0.0 | 0.0 | 0.0 |
| 5 | 0.8 | 0.8 | 0.8 | 0.2 | 0.2 | 0.2 | 1.0 | 1.0 | 1.0 | 0.0 | 0.0 | 0.0 |
| **Scenario 17 (SNP-set 1: maternal-fetal interaction, SNP-set 2: epistatic maternally-mediated effect)** | | | | | | | | | | | | |
| 2 | 0.6 | 0.6 | - | 0.8 | 0.8 | - | 1.0 | 1.0 | 0.4 | 0.4 | - | - |
| 3 | 0.9 | 0.9 | 0.9 | 0.9 | 0.9 | 0.9 | 1.0 | 1.0 | 1.0 | 0.8 | 0.8 | 0.8 |
| 4 | 0.2 | 0.2 | 0.2 | 0.8 | 0.8 | 0.8 | 1.0 | 1.0 | 1.0 | 0.0 | 0.0 | 0.0 |
| 5 | 0.2 | 0.2 | 0.2 | 0.8 | 0.8 | 0.8 | 1.0 | 1.0 | 1.0 | 0.0 | 0.0 | 0.0 |
| **Scenario 18 (SNP-set 1: maternal-fetal interaction, SNP-set 2: epistatic child-SNP interaction)** | | | | | | | | | | | | |
| 2 | 0.7 | 0.7 | - | 0.8 | 0.8 | - | 1.0 | 1.0 | 0.5 | 0.5 | - | - |
| 3 | 0.8 | 0.8 | 0.8 | 1.0 | 1.0 | 1.0 | 1.0 | 1.0 | 1.0 | 0.8 | 0.8 | 0.8 |
| 4 | 0.5 | 0.5 | 0.5 | 0.5 | 0.5 | 0.5 | 1.0 | 1.0 | 1.0 | 0.0 | 0.0 | 0.0 |
| 5 | 0.4 | 0.4 | 0.4 | 0.7 | 0.7 | 0.7 | 1.0 | 1.0 | 1.0 | 0.1 | 0.1 | 0.1 |
| **Scenario 19 (SNP-set 1: epistatic maternally-mediated effect, SNP-set 2: epistatic child-SNP interaction)** | | | | | | | | | | | | |
| 2 | 0.6 | 0.6 | - | 0.6 | 0.6 | - | 1.0 | 1.0 | 0.2 | 0.2 | - | - |
| 3 | 0.7 | 0.7 | 0.7 | 0.8 | 0.8 | 0.8 | 1.0 | 1.0 | 1.0 | 0.5 | 0.5 | 0.5 |
| 4 | 0.5 | 0.5 | 0.5 | 0.5 | 0.5 | 0.5 | 1.0 | 1.0 | 1.0 | 0.0 | 0.0 | 0.0 |
| 5 | 0.5 | 0.5 | 0.5 | 0.5 | 0.5 | 0.5 | 1.0 | 1.0 | 1.0 | 0.0 | 0.0 | 0.0 |
| **Scenario 20 (two SNP-sets with epistatic child-SNP interactions)** | | | | | | | | | | | | |
| 2 | 0.7 | 0.7 | - | 0.5 | 0.5 | - | 1.0 | 1.0 | 0.2 | 0.2 | - | - |
| 3 | 0.8 | 0.8 | 0.8 | 0.8 | 0.8 | 0.8 | 1.0 | 1.0 | 1.0 | 0.6 | 0.6 | 0.6 |
| 4 | 0.5 | 0.5 | 0.5 | 0.5 | 0.5 | 0.5 | 1.0 | 1.0 | 1.0 | 0.0 | 0.0 | 0.0 |
| 5 | 0.5 | 0.5 | 0.5 | 0.5 | 0.5 | 0.5 | 1.0 | 1.0 | 1.0 | 0.0 | 0.0 | 0.0 |

**Supplementary Table S4**. Comparison of GADGETS with competitors in finding a simulated maternal-fetal genetic interaction over a range of input SNP numbers. The simulation scenario is a modified version of Scenario 1; it involves a single risk-related SNP-set with one maternal and two child SNPs. Due to limitations in the number of input SNPs that competitors could analyze with reasonable run times, the number of candidate SNPs was at most 500. For each replicate, half of the input SNPs are child SNPs, and the remainder are the corresponding maternal SNPs. Run times are hours:minutes:seconds.

| Input SNPs | GADGETS | | MDR-PDT | | TrioFS | | EPISFA-LD | |
| --- | --- | --- | --- | --- | --- | --- | --- | --- |
|  | Max Risk SNPs Found (Rank)^a^ | Run Time | Max Risk SNPs Found (Rank)^a^ | Run Time | Max Risk SNPs Found (Rank)^a^ | Run Time | Max Risk SNPs Found (Rank)^ab^ | Run Time |
| **Replicate 1** | | | | | | | | |
| 24 | 3(1) | 00:01:42 | 3(7) | 00:00:04 | 3(1) | 00:45:03 | 3[0](Top 3) | 00:00:32 |
| 100 | 3(1) | 00:01:40 | 2(1) | 00:05:07 | 3(2) | 00:41:18 | 3[0](Top 4) | 00:02:34 |
| 500 | 3(1) | 00:07:32 | 2(2) | 10:02:40 | 0 | 03:45:45 | ** | ** |
| **Replicate 2** | | | | | | | | |
| 24 | 3(1) | 00:01:31 | 2(1) | 00:00:04 | 3(1) | 00:38:14 | 3[2](1) | 00:01:01 |
| 100 | 3(1) | 00:01:29 | 3(2) | 00:05:32 | 2(1) | 00:48:19 | 3[1](Top 2) | 00:03:02 |
| 500 | 3(1) | 00:07:12 | 2(1) | 10:10:29 | 2(1) | 03:44:53 | ** | ** |
| **Replicate 3** | | | | | | | | |
| 24 | 3(1) | 00:01:27 | 2(1) | 00:00:05 | 3(1) | 00:45:34 | 3[2](1) | 00:00:36 |
| 100 | 3(1) | 00:01:31 | 2(1) | 00:05:32 | 3(3) | 00:47:43 | 3[2](1) | 00:01:48 |
| 500 | 3(1) | 00:07:17 | 2(1) | 10:15:26 | 2(1) | 04:02:30 | ** | ** |
| **Replicate 4** | | | | | | | | |
| 24 | 3(1) | 00:01:29 | 2(1) | 00:00:06 | 3(1) | 00:46:27 | 3[1](Top 2) | 00:00:30 |
| 100 | 3(1) | 00:01:34 | 2(1) | 00:04:40 | 3(2) | 00:49:27 | 3[1](1) | 00:01:44 |
| 500 | 3(1) | 00:07:15 | 2(1) | 10:09:03 | 2(1) | 03:27:00 | ** | ** |
| **Replicate 5** | | | | | | | | |
| 24 | 3(1) | 00:01:36 | 2(1) | 00:00:04 | 3(1) | 00:45:50 | 3[0](Top 4) | 00:00:39 |
| 100 | 3(1) | 00:01:32 | 2(1) | 00:04:39 | 3(1) | 00:46:35 | 3[0](Top 2) | 00:02:06 |
| 500 | 3(1) | 00:07:23 | 2(1) | 09:54:51 | 2(1) | 03:45:15 | ** | ** |
| **Replicate 6** | | | | | | | | |
| 24 | 3(1) | 00:01:28 | 2(1) | 00:00:04 | 3(1) | 00:45:25 | 3[2](Top 2) | 00:00:29 |
| 100 | 3(1) | 00:01:33 | 2(1) | 00:04:48 | 3(2) | 00:47:44 | 3[1](Top 3) | 00:01:43 |
| 500 | 3(1) | 00:07:15 | 2(1) | 09:03:49 | 3(2) | 04:00:52 | ** | ** |
| **Replicate 7** | | | | | | | | |
| 24 | 3(1) | 00:01:29 | 2(1) | 00:00:04 | 3(1) | 00:45:36 | 3[1](1) | 00:00:54 |
| 100 | 3(1) | 00:01:33 | 2(1) | 00:04:44 | 3(3) | 00:47:58 | 3[1](1) | 00:03:46 |
| 500 | 3(1) | 00:07:17 | 2(1) | 10:14:08 | 1(10) | 03:57:50 | ** | ** |
| **Replicate 8** | | | | | | | | |
| 24 | 3(1) | 00:01:29 | 3(1) | 00:00:05 | 3(1) | 00:46:31 | 3[1](1) | 00:00:33 |
| 100 | 3(1) | 00:01:30 | 3(1) | 00:04:49 | 3(3) | 00:48:27 | 3[1](1) | 00:01:49 |
| 500 | 3(1) | 00:07:10 | 3(1) | 10:39:06 | 2(1) | 03:54:05 | ** | ** |
| **Replicate 9** | | | | | | | | |
| 24 | 3(1) | 00:01:30 | 2(1) | 00:00:05 | 3(1) | 00:45:29 | 3[1](Top 2) | 00:00:38 |
| 100 | 3(1) | 00:01:31 | 2(1) | 00:04:52 | 3(3) | 00:47:53 | 3[0](Top 2) | 00:03:02 |
| 500 | 3(1) | 00:07:16 | 2(1) | 09:49:45 | 3(1) | 03:42:37 | ** | ** |
| **Replicate 10** | | | | | | | | |
| 24 | 3(1) | 00:01:27 | 3(4) | 00:00:05 | 3(1) | 00:44:11 | 3[1](Top 3) | 00:00:43 |
| 100 | 3(1) | 00:01:29 | 2(1) | 00:04:51 | 3(1) | 00:46:56 | 3[0](Top 2) | 00:02:54 |
| 500 | 3(1) | 00:07:07 | 2(1) | 10:07:39 | 3(1) | 03:43:45 | ** | ** |

^a^Maximum number of SNPs contained in the risk-related SNP-set in any single SNP-set/model among the top 10 highest ranking SNP-sets/models, and corresponding SNP-set/model rank (1 = highest). Zero is reported when models failed to identify any risk-related SNPs. ** is reported when the software was unable to output results.

^b^Square brackets indicate the number of non-risk-related SNPs returned by EPISFA-LD; it can return sets of any size, rather than a pre-specified size of interest.

**Supplementary Table S5**. Comparison of GADGETS with competitors in finding a simulated epistatic maternally-mediated genetic interaction over a range of input SNP numbers. The simulation scenario is a modified version of Scenario 11; it involves a single risk-related SNP-set with three maternal SNPs. Due to limitations in the number of input SNPs that competitors could analyze with reasonable run times, the number of candidate SNPs was at most 500. For each replicate, half of the input SNPs are child SNPs, and the remainder are the corresponding maternal SNPs. Run times are hours:minutes:seconds.

| Input SNPs | GADGETS | | MDR-PDT | | TrioFS | | EPISFA-LD | |
| --- | --- | --- | --- | --- | --- | --- | --- | --- |
|  | Max Risk SNPs Found (Rank)^a^ | Run Time | Max Risk SNPs Found (Rank)^a^ | Run Time | Max Risk SNPs Found (Rank)^a^ | Run Time | Max Risk SNPs Found (Rank)^ab^ | Run Time |
| **Replicate 1** | | | | | | | | |
| 24 | 3(1) | 00:01:26 | 2(1) | 00:00:04 | 3(1) | 00:45:01 | 3[2](1) | 00:00:32 |
| 100 | 3(1) | 00:01:28 | 2(1) | 00:05:49 | 2(1) | 00:47:48 | 3[1](Top 2) | 00:01:33 |
| 500 | 3(1) | 00:07:16 | 2(1) | 09:19:19 | 2(1) | 03:36:17 | ** | ** |
| **Replicate 2** | | | | | | | | |
| 24 | 3(1) | 00:01:30 | 2(1) | 00:00:04 | 3(1) | 00:45:33 | 3[0](Top 3) | 00:00:47 |
| 100 | 3(1) | 00:01:33 | 2(1) | 00:06:05 | 2(1) | 00:41:47 | 3[0](Top 2) | 00:03:01 |
| 500 | 3(1) | 00:07:16 | 2(1) | 09:11:30 | 2(1) | 03:25:30 | ** | ** |
| **Replicate 3** | | | | | | | | |
| 24 | 3(1) | 00:01:22 | 2(1) | 00:00:05 | 3(1) | 00:44:11 | 3[1](Top 2) | 00:00:46 |
| 100 | 3(1) | 00:01:31 | 2(1) | 00:04:49 | 3(4) | 00:49:35 | 3[1](Top 2) | 00:02:24 |
| 500 | 3(1) | 00:07:16 | 2(1) | 10:03:02 | 2(1) | 03:56:00 | ** | ** |
| **Replicate 4** | | | | | | | | |
| 24 | 3(1) | 00:01:31 | 2(1) | 00:00:05 | 3(1) | 00:45:42 | 3[1](Top 3) | 00:00:39 |
| 100 | 3(1) | 00:01:29 | 2(1) | 00:05:06 | 3(3) | 00:49:17 | 3[1](Top 2) | 00:02:08 |
| 500 | 3(1) | 00:07:17 | 2(1) | 09:44:05 | 2(1) | 03:27:14 | ** | ** |
| **Replicate 5** | | | | | | | | |
| 24 | 3(1) | 00:01:30 | 2(1) | 00:00:05 | 3(1) | 00:49:02 | 3[1](Top 2) | 00:00:38 |
| 100 | 3(1) | 00:01:31 | 2(1) | 00:05:14 | 3(2) | 00:47:46 | 3[1](Top 2) | 00:02:17 |
| 500 | 3(1) | 00:07:23 | 2(1) | 09:45:59 | 2(1) | 03:54:43 | ** | ** |
| **Replicate 6** | | | | | | | | |
| 24 | 3(1) | 00:01:34 | 3(3) | 00:00:05 | 3(1) | 00:38:30 | 3[2](Top 2) | 00:00:35 |
| 100 | 3(1) | 00:01:34 | 3(4) | 00:05:35 | 2(1) | 00:46:55 | 3[1](Top 4) | 00:01:44 |
| 500 | 3(1) | 00:07:16 | 2(1) | 10:02:58 | 2(1) | 04:02:43 | ** | ** |
| **Replicate 7** | | | | | | | | |
| 24 | 3(1) | 00:01:29 | 2(1) | 00:00:05 | 3(1) | 00:50:29 | 3[2](1) | 00:01:04 |
| 100 | 3(1) | 00:01:30 | 2(1) | 00:06:16 | 2(1) | 00:46:56 | 3[2](1) | 00:05:08 |
| 500 | 3(1) | 00:07:28 | 2(1) | 10:05:29 | 2(1) | 04:02:23 | ** | ** |
| **Replicate 8** | | | | | | | | |
| 24 | 3(1) | 00:01:33 | 2(1) | 00:00:05 | 3(1) | 00:44:37 | 3[2](1) | 00:00:36 |
| 100 | 3(1) | 00:01:30 | 2(1) | 00:06:29 | 2(1) | 00:51:41 | 3[1](Top 2) | 00:01:47 |
| 500 | 3(1) | 00:07:20 | 2(1) | 10:31:26 | 2(1) | 04:00:46 | ** | ** |
| **Replicate 9** | | | | | | | | |
| 24 | 3(1) | 00:01:28 | 3(2) | 00:00:05 | 3(1) | 00:44:54 | 3[1](Top 2) | 00:00:30 |
| 100 | 3(1) | 00:01:30 | 3(5) | 00:06:29 | 3(3) | 00:50:48 | 3[1](1) | 00:02:53 |
| 500 | 3(1) | 00:07:22 | 2(1) | 10:31:57 | 2(1) | 03:34:16 | ** | ** |
| **Replicate 10** | | | | | | | | |
| 24 | 3(1) | 00:01:34 | 3(9) | 00:00:06 | 3(1) | 00:39:51 | 3[1](Top 2) | 00:00:49 |
| 100 | 3(1) | 00:01:33 | 2(1) | 00:04:31 | 3(3) | 00:50:47 | 3[1](Top 3) | 00:01:45 |
| 500 | 3(1) | 00:07:05 | 2(1) | 10:29:37 | 2(1) | 03:34:44 | ** | ** |

^a^Maximum number of SNPs contained in the risk-related SNP-set in any single SNP-set/model among the top 10 highest ranking SNP-sets/models, and corresponding SNP-set/model rank (1 = highest). Zero is reported when models failed to identify any risk-related SNPs. ** is reported when the software was unable to output results.

^b^Square brackets indicate the number of non-risk-related SNPs returned by EPISFA-LD; it can return sets of any size, rather than a pre-specified size of interest.

**Supplementary Table S6**. Comparison of GADGETS with competitors in finding a simulated epistatic maternally-mediated genetic interaction over a range of input SNP numbers. The simulation scenario is a modified version of Scenario 12; it involves a single risk-related SNP-set with three maternal SNPs. Due to limitations in the number of input SNPs that competitors could analyze with reasonable run times, the number of candidate SNPs was at most 500. For each replicate, half of the input SNPs are child SNPs, and the remainder are the corresponding maternal SNPs. Run times are hours:minutes:seconds.

| Input SNPs | GADGETS | | MDR-PDT | | TrioFS | | EPISFA-LD | |
| --- | --- | --- | --- | --- | --- | --- | --- | --- |
|  | Max Risk SNPs Found (Rank)^a^ | Run Time | Max Risk SNPs Found (Rank)^a^ | Run Time | Max Risk SNPs Found (Rank)^a^ | Run Time | Max Risk SNPs Found (Rank)^ab^ | Run Time |
| **Replicate 1** | | | | | | | | |
| 24 | 3(1) | 00:01:24 | 3(1) | 00:00:05 | 3(1) | 00:39:25 | 3[1](Top 2) | 00:00:26 |
| 100 | 3(1) | 00:01:31 | 3(6) | 00:05:29 | 2(1) | 00:49:43 | 3[1](1) | 00:01:53 |
| 500 | 3(1) | 00:07:11 | 2(1) | 10:00:55 | 2(1) | 03:39:54 | ** | ** |
| **Replicate 2** | | | | | | | | |
| 24 | 3(1) | 00:01:24 | 2(3) | 00:00:05 | 3(1) | 00:50:36 | 3[0](Top 3) | 00:01:12 |
| 100 | 3(1) | 00:01:29 | 2(7) | 00:04:47 | 2(1) | 00:48:53 | 3[1](Top 2) | 00:04:16 |
| 500 | 3(1) | 00:07:07 | 1(7) | 09:59:08 | 0 | 03:40:24 | ** | ** |
| **Replicate 3** | | | | | | | | |
| 24 | 3(1) | 00:01:24 | 2(1) | 00:00:04 | 3(1) | 00:47:52 | 3[0](1) | 00:00:41 |
| 100 | 3(1) | 00:01:32 | 2(2) | 00:04:53 | 2(2) | 00:46:52 | 3[0](1) | 00:04:18 |
| 500 | 3(1) | 00:07:16 | 0 | 10:05:39 | 2(2) | 03:39:41 | ** | ** |
| **Replicate 4** | | | | | | | | |
| 24 | 3(1) | 00:01:31 | 2(1) | 00:00:04 | 3(1) | 00:49:21 | 3[2](1) | 00:00:35 |
| 100 | 3(1) | 00:01:30 | 2(1) | 00:04:54 | 2(1) | 00:49:01 | 3[2](1) | 00:01:53 |
| 500 | 3(1) | 00:07:17 | 2(1) | 09:45:13 | 2(1) | 03:57:01 | ** | ** |
| **Replicate 5** | | | | | | | | |
| 24 | 3(1) | 00:01:26 | 2(1) | 00:00:05 | 3(1) | 00:45:48 | 3[1](1) | 00:00:39 |
| 100 | 3(1) | 00:01:29 | 2(1) | 00:04:55 | 2(1) | 00:40:56 | 3[1](1) | 00:02:06 |
| 500 | 3(1) | 00:07:07 | 2(1) | 10:00:45 | 2(1) | 03:49:48 | ** | ** |
| **Replicate 6** | | | | | | | | |
| 24 | 3(1) | 00:01:25 | 2(1) | 00:00:05 | 3(1) | 00:48:00 | 3[1](1) | 00:01:35 |
| 100 | 3(1) | 00:01:33 | 2(1) | 00:04:52 | 2(3) | 00:47:28 | 3[1](1) | 00:03:48 |
| 500 | 3(1) | 00:07:15 | 1(2) | 10:42:15 | 0 | 04:02:39 | ** | ** |
| **Replicate 7** | | | | | | | | |
| 24 | 3(1) | 00:01:26 | 2(1) | 00:00:05 | 3(1) | 00:49:16 | 3[1](Top 2) | 00:00:41 |
| 100 | 3(1) | 00:01:32 | 3(6) | 00:05:54 | 2(1) | 00:47:06 | 3[0](Top 2) | 00:02:17 |
| 500 | 3(1) | 00:07:06 | 2(1) | 10:43:52 | 2(1) | 03:28:54 | ** | ** |
| **Replicate 8** | | | | | | | | |
| 24 | 3(1) | 00:01:29 | 2(1) | 00:00:05 | 3(1) | 00:48:46 | 3[1](1) | 00:00:45 |
| 100 | 3(1) | 00:01:33 | 2(3) | 00:05:53 | 3(1) | 00:50:41 | 3[1](1) | 00:01:45 |
| 500 | 3(1) | 00:07:19 | 0 | 10:47:26 | 2(5) | 04:06:32 | ** | ** |
| **Replicate 9** | | | | | | | | |
| 24 | 3(1) | 00:01:23 | 3(8) | 00:00:05 | 3(1) | 00:48:18 | 3[1](Top 2) | 00:00:42 |
| 100 | 3(1) | 00:01:32 | 2(5) | 00:04:47 | 2(1) | 00:48:03 | 3[1](1) | 00:01:40 |
| 500 | 3(1) | 00:07:10 | 1(8) | 10:06:25 | 2(1) | 03:55:46 | ** | ** |
| **Replicate 10** | | | | | | | | |
| 24 | 3(1) | 00:01:27 | 2(1) | 00:00:05 | 3(1) | 00:45:23 | 3[0](1) | 00:00:40 |
| 100 | 3(1) | 00:01:31 | 2(4) | 00:04:42 | 2(1) | 00:48:21 | 3[0](1) | 00:02:07 |
| 500 | 3(1) | 00:07:07 | 2(1) | 10:12:02 | 1(5) | 03:38:10 | ** | ** |

^a^Maximum number of SNPs contained in the risk-related SNP-set in any single SNP-set/model among the top 10 highest ranking SNP-sets/models, and corresponding SNP-set/model rank (1 = highest). Zero is reported when models failed to identify any risk-related SNPs. ** is reported when the software was unable to output results.

^b^Square brackets indicate the number of non-risk-related SNPs returned by EPISFA-LD; it can return sets of any size, rather than a pre-specified size of interest.

## Supplementary Figures

**Supplementary Figure S1. Illustration of GADGETS’s epistasis test.** For this illustration, we consider a SNP-set of five component SNPs. The fourth and fifth SNPs in the set are assumed in linkage. The test is applicable when at least one pair of loci is assumed unlinked. Below, we shorten ‘complement-siblings' to ‘complements’. Matrix cells represent genotypes, columns represent SNPs, and rows correspond to families. The procedure involves randomly shuffling genotypes for each unlinked locus, maintaining the observed case/complement-sibling pairs. When component SNPs are assumed linked, those genotypes are shuffled as a unit. Conducting permutations in this way will preserve marginal effects in the permuted data (and joint effects, for loci assumed linked under the null), but should destroy any joint effects across unlinked SNPs. When run on data independent of that used by GADGETS, the ‘h-value’ from this procedure should be uniformly distributed under the no-epistasis null hypothesis and is a valid p-value. Because we ran the epistasis test on the same data that GADGETS used, h-values will not be uniformly distributed under the null. Regardless, a lower value suggests less evidence for the null hypothesis.


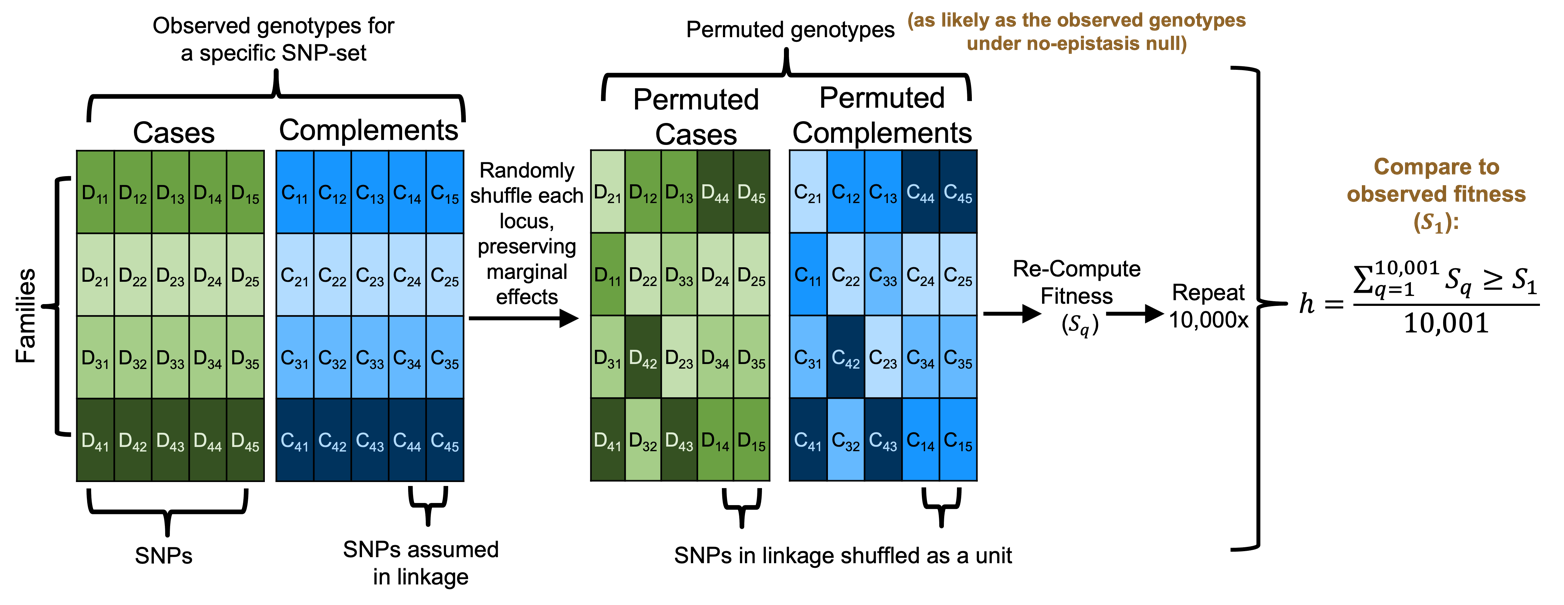


**Supplementary Figure S2. Illustration of GADGETS’s maternal-fetal interaction test.** The test is a modification of the epistasis test; the null hypothesis is now that there are no maternal-fetal interaction effects among the component SNPs in the SNP-set. For this illustration, we consider a SNP-set of five component SNPs. The first three loci are child SNPs and the last two are maternal. We assume all component SNPs are unlinked: the procedure is applicable to any SNP-set in which, among all possible maternal-SNP/child-SNP pairs, no pair of loci is assumed linked under the null. The procedure involves randomly permuting the maternal/paternal genotype pairs jointly as a unit, and separately doing so for case/complement-sibling pairs. Doing so will preserve any effects that only involve the maternal SNPs or only involve child SNPs but will destroy any maternal-fetal effects. In the diagram, matrix cells represent genotypes, columns represent SNPs, and rows correspond to families. When run on data independent of that used by GADGETS, the h-value from this procedure should be uniformly distributed under the no-maternal-fetal-interaction null hypothesis and is a valid p-value. Because we use the same data that GADGETS used, h-values will not be uniformly distributed under the null. Regardless, a lower value suggests less evidence for the null hypothesis.


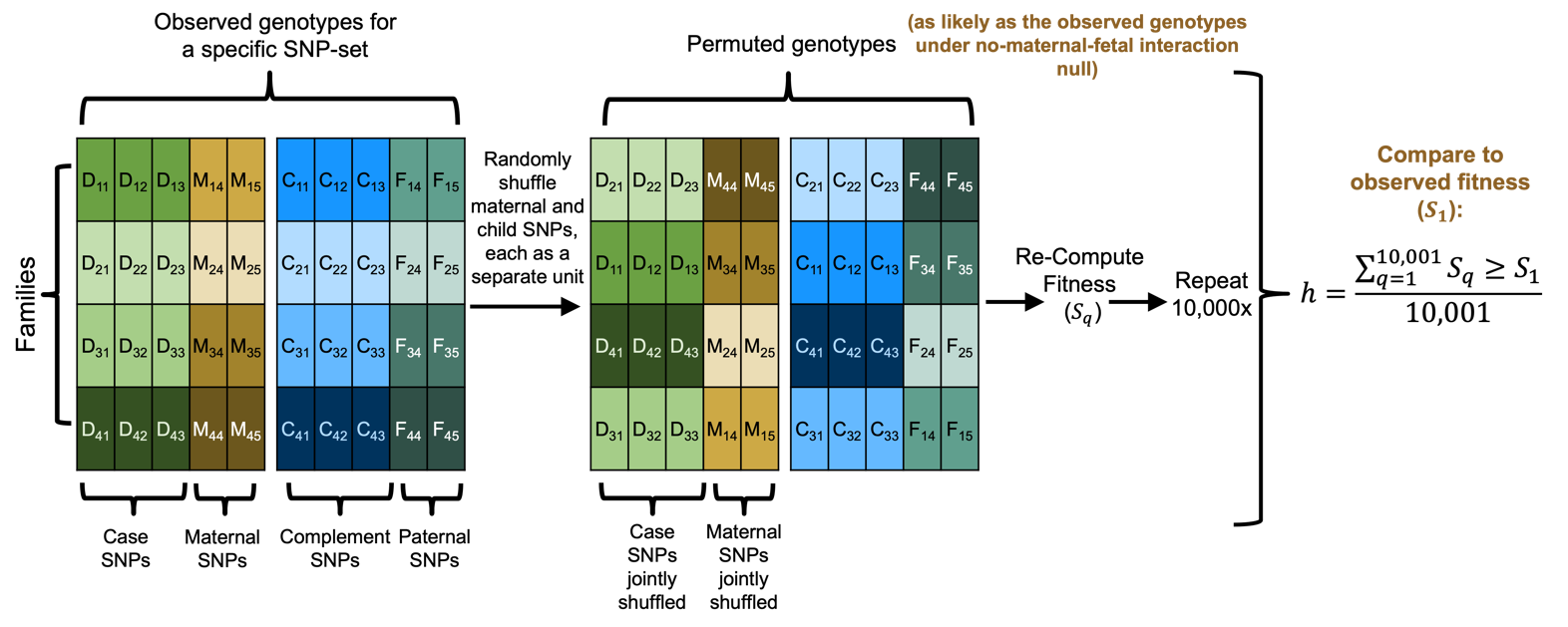


**Supplementary Figure S3. Network plot for simulation scenario 1, replicate 5.** Circles represent child SNPs and squares represent maternal SNPs. SNP label ‘1:’ indicates membership in the SNP-set with a simulated maternal-fetal interaction effect. The number following the colon is the simulated SNP's identifier. A SNP with no colon in the label is not-risk related. Maternal and child SNPs with the same identifier represent the same locus. The SNP-sets that contributed to this plot were selected using the method described by Nodzenski *et al.* (Nodzenski et al., 2022). After applying that filter, we plotted all 51 SNP-pairs (comprising 18 SNPs) that received graphical scores. Thicker, darker connections indicate higher SNP-pair graphical scores; larger, darker vertices indicate higher individual SNP graphical scores.


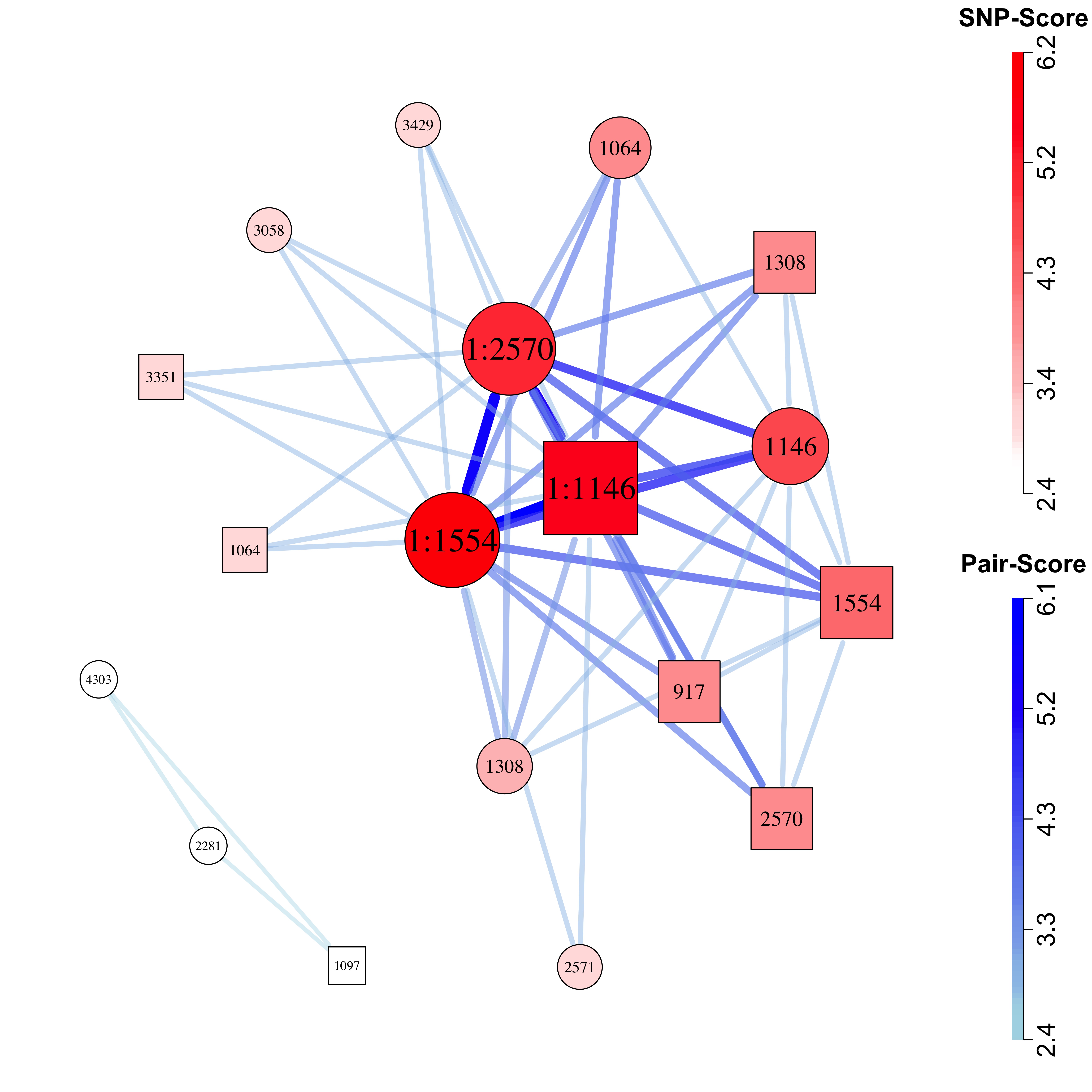


**Supplementary Figure S4. Network plot for simulation scenario 2, replicate 6.** Circles represent child SNPs and squares represent maternal SNPs. SNP label ‘1:’ indicates membership in the SNP-set with a simulated maternal-fetal interaction effect. The number following the colon is the simulated SNP's identifier. A SNP with no colon in the label is not-risk related. Maternal and child SNPs with the same identifier represent the same locus. The SNP-sets that contributed to this plot were selected using the method described by Nodzenski *et al.*(Nodzenski et al., 2022) . After applying that filter, we plotted all 58 SNP-pairs (comprising 18 SNPs) that received graphical scores. Thicker, darker connections indicate higher SNP-pair graphical scores; larger, darker vertices indicate higher individual SNP graphical scores.


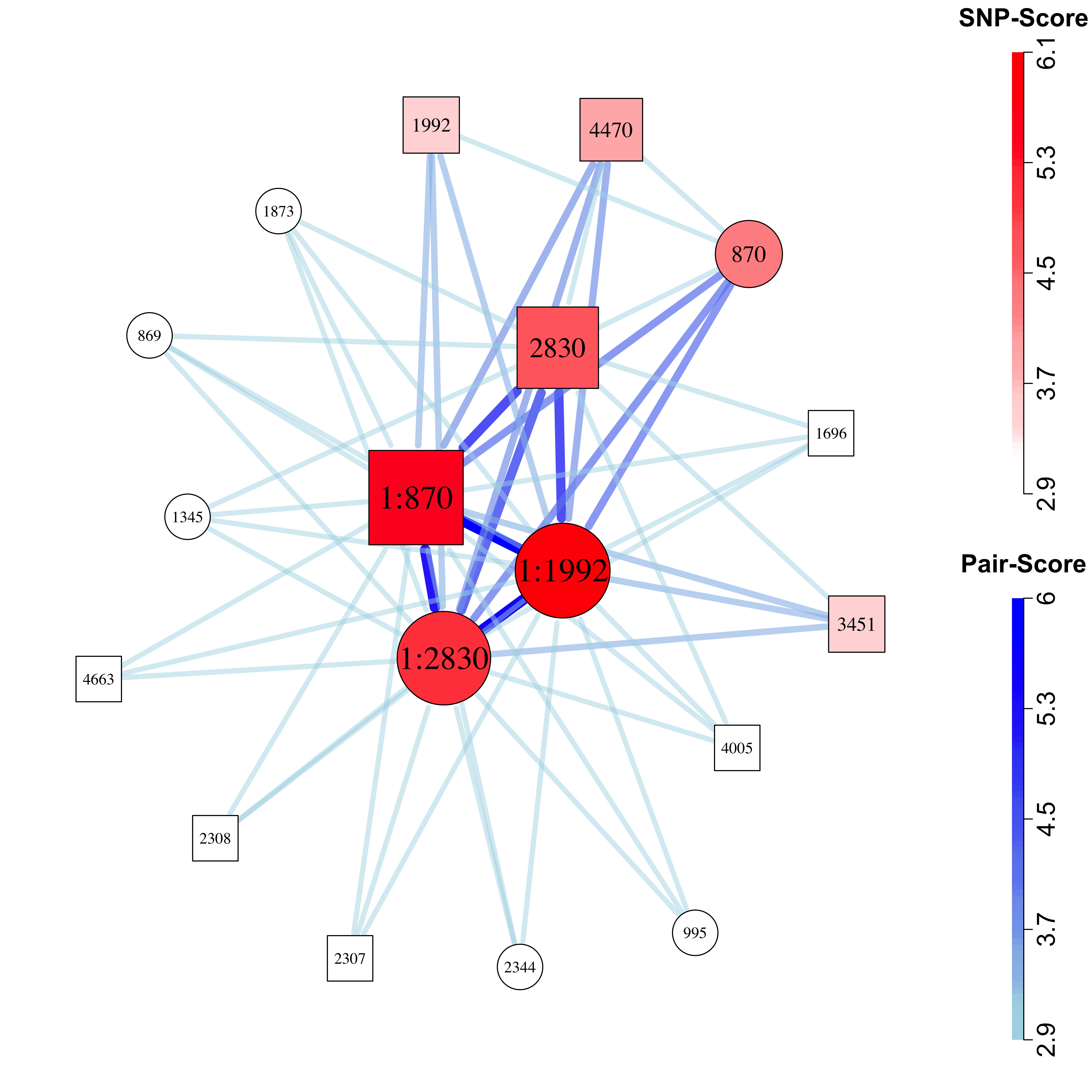


Supplementary Figure S5. Network plot for simulation scenario 3, replicate 9. Circles represent child SNPs and squares represent maternal SNPs. SNP label ‘1:’ indicates membership in the SNP-set with a simulated maternal-fetal interaction effect. The number following the colon is the simulated SNP's identifier. A SNP with no colon in the label is not-risk related. Maternal and child SNPs with the same identifier represent the same The SNP-sets that contributed to this plot were selected using the method described by Nodzenski *et al.*(Nodzenski et al., 2022) . After applying that filter, we plotted all 69 SNP-pairs (comprising 33 SNPs) that received graphical scores. Thicker, darker connections indicate higher SNP-pair graphical scores; larger, darker vertices indicate higher individual SNP graphical scores.


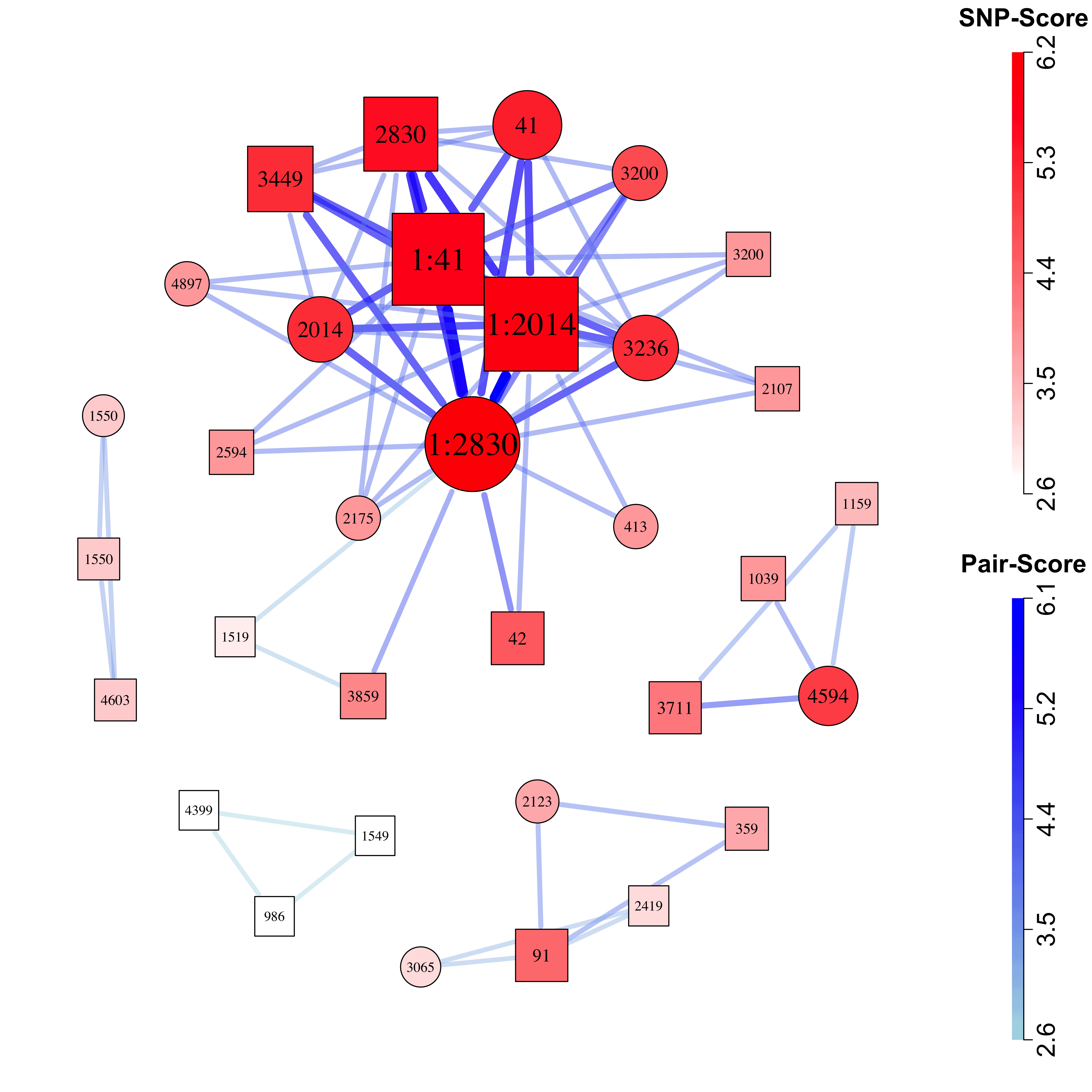


**Supplementary Figure S6. Network plot for simulation scenario 4, replicate 2.** Circles represent child SNPs and squares represent maternal SNPs. SNP label ‘1:’ indicates membership in the SNP-set with a simulated maternal-fetal interaction effect. The number following the colon is the simulated SNP's identifier. A SNP with no colon in the label is not-risk related. Maternal and child SNPs with the same identifier represent the same locus. The SNP-sets that contributed to this plot were selected using the method described by Nodzenski *et al.*(Nodzenski et al., 2022) . After applying that filter, we plotted all 70 SNP-pairs (comprising 30 SNPs) that received graphical scores. Thicker, darker connections indicate higher SNP-pair graphical scores; larger, darker vertices indicate higher individual SNP graphical scores.


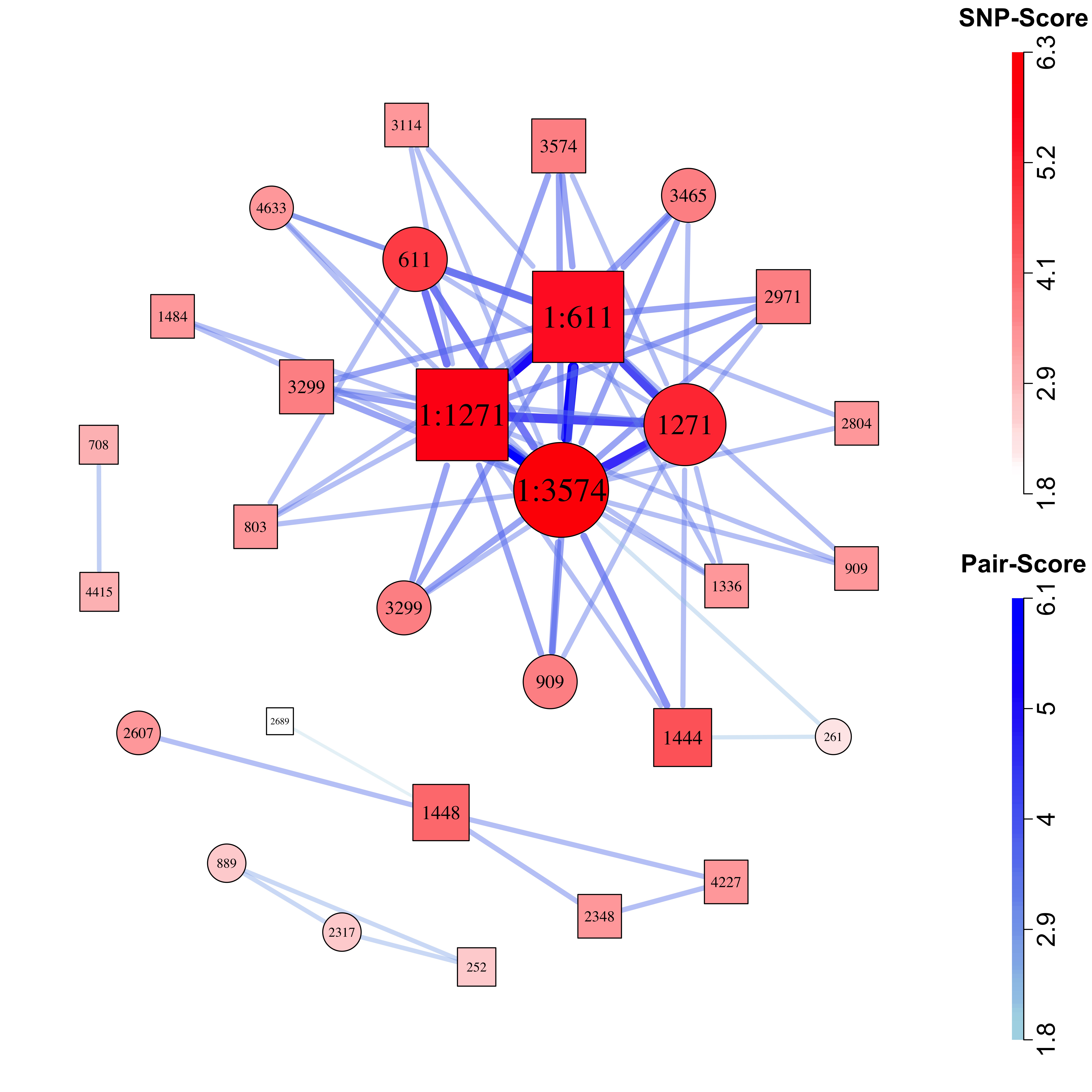


**Supplementary Figure S7. Network plot for simulation scenario 5, replicate 4.** Circles represent child SNPs and squares represent maternal SNPs. SNP label ‘1:’ indicates membership in the SNP-set with a simulated maternal-fetal interaction effect. The number following the colon is the simulated SNP's identifier. A SNP with no colon in the label is not-risk related. Maternal and child SNPs with the same identifier represent the same locus. The SNP-sets that contributed to this plot were selected using the method described by Nodzenski *et al.* (Nodzenski et al., 2022) . After applying that filter, we plotted all 70 SNP-pairs (comprising 29 SNPs) that received graphical scores. Thicker, darker connections indicate higher SNP-pair graphical scores; larger, darker vertices indicate higher individual SNP graphical scores.


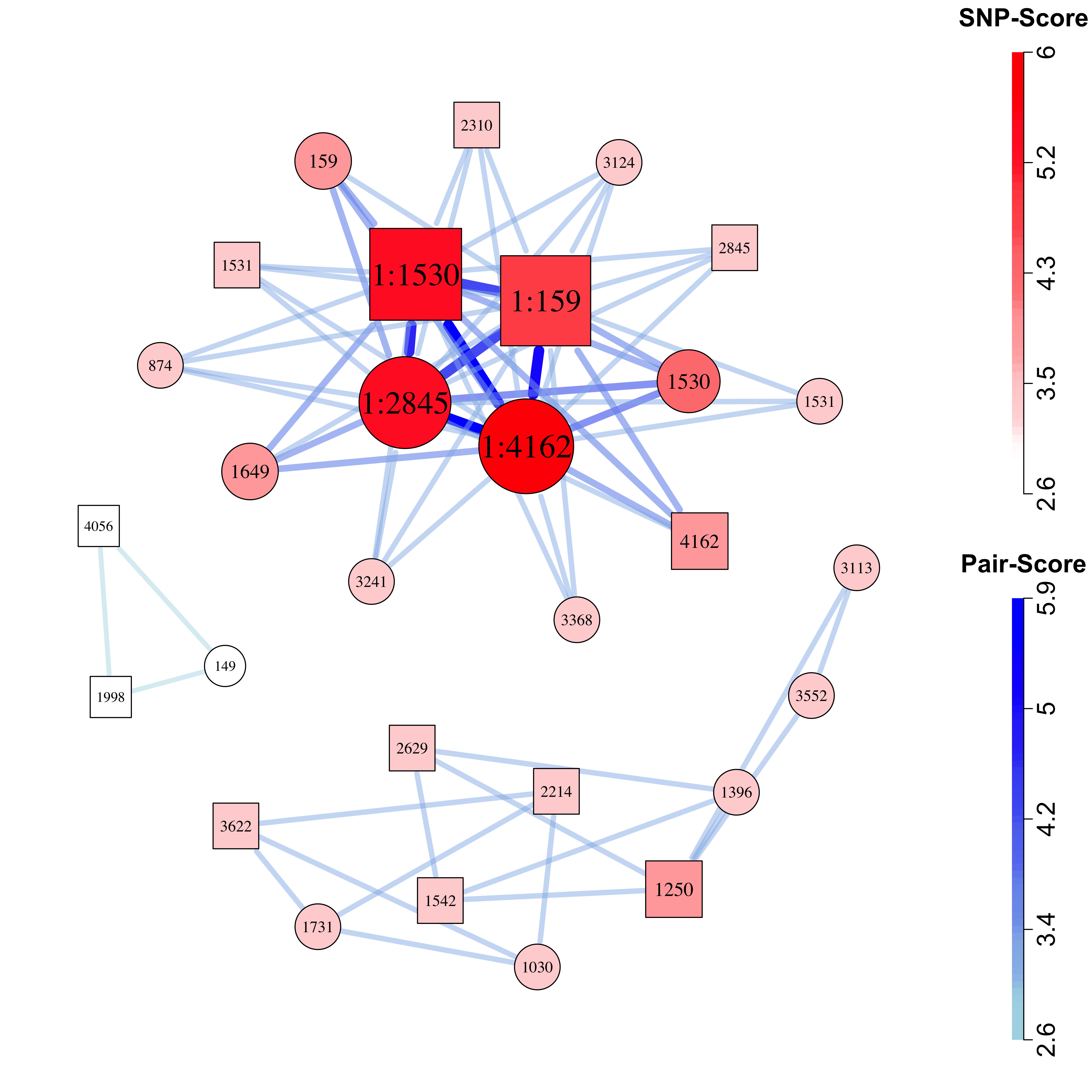


**Supplementary Figure S8. Network plot for simulation scenario 6, replicate 10.** Circles represent child SNPs and squares represent maternal SNPs. SNP label ‘1:’ indicates membership in the SNP-set with a simulated maternal-fetal interaction effect. The number following the colon is the simulated SNP's identifier. A SNP with no colon in the label is not-risk related. Maternal and child SNPs with the same identifier represent the same locus. The SNP-sets that contributed to this plot were selected using the method described by Nodzenski *et al.*(Nodzenski et al., 2022) . After applying that filter, we plotted all 89 SNP-pairs (comprising 39 SNPs) that received graphical scores. Thicker, darker connections indicate higher SNP-pair graphical scores; larger, darker vertices indicate higher individual SNP graphical scores.


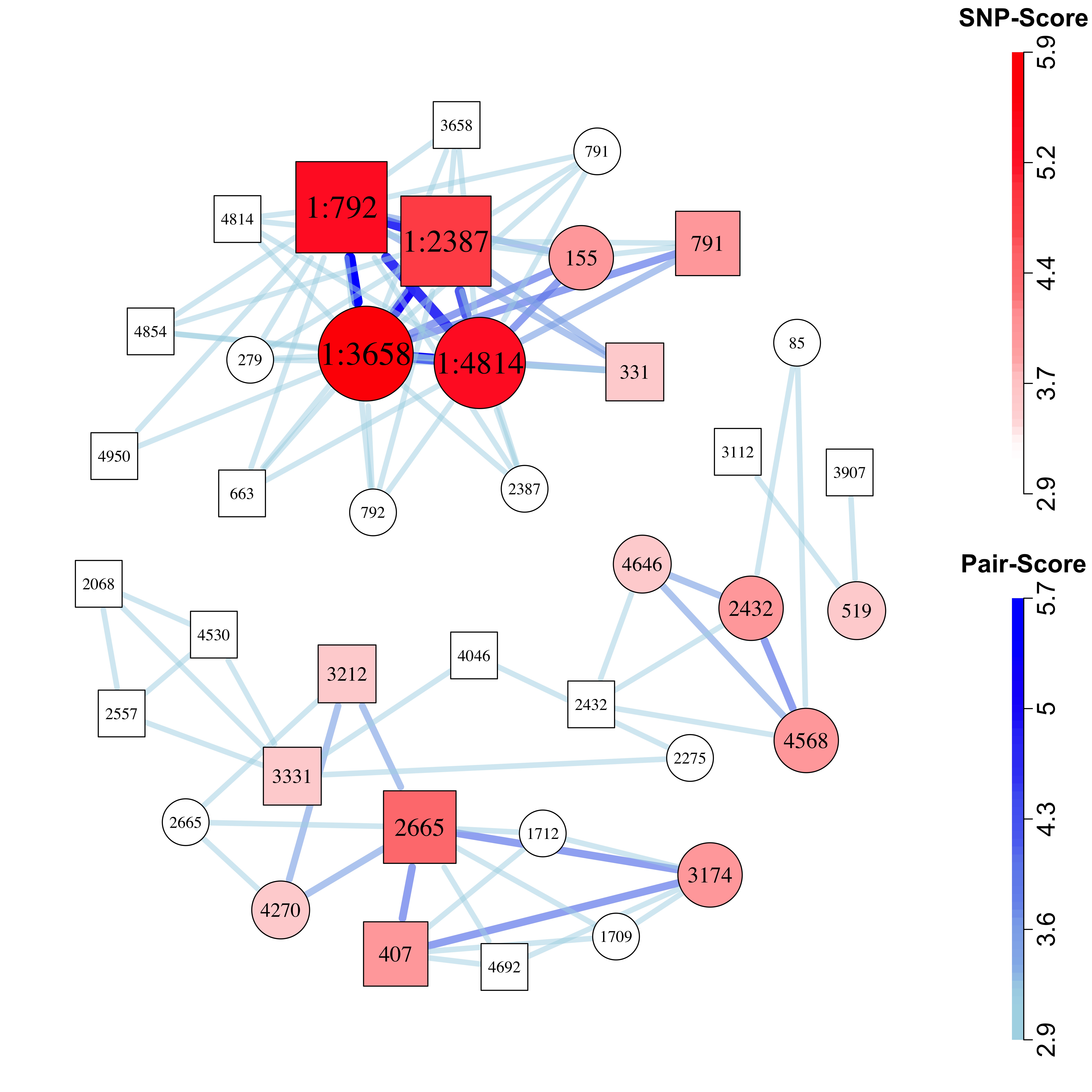


**Supplementary Figure S9. Network plot for simulation scenario 7, replicate 7.** Circles represent child SNPs and squares represent maternal SNPs. SNP label ‘1:’ indicates membership in the risk-related SNP-set with a maternal-fetal interaction effect. A SNP with no colon in the label is not-risk related. The number following the colon is the simulated SNP's identifier. Maternal and child SNPs with the same identifier represent the same locus. The SNP-sets that contributed to this plot were selected using the method described by Nodzenski *et al.*(Nodzenski et al., 2022) . After applying that filter, we plotted all 55 SNP-pairs (comprising 17 SNPs) that received graphical scores. Thicker, darker connections indicate higher SNP-pair graphical scores; larger, darker vertices indicate higher individual SNP graphical scores.


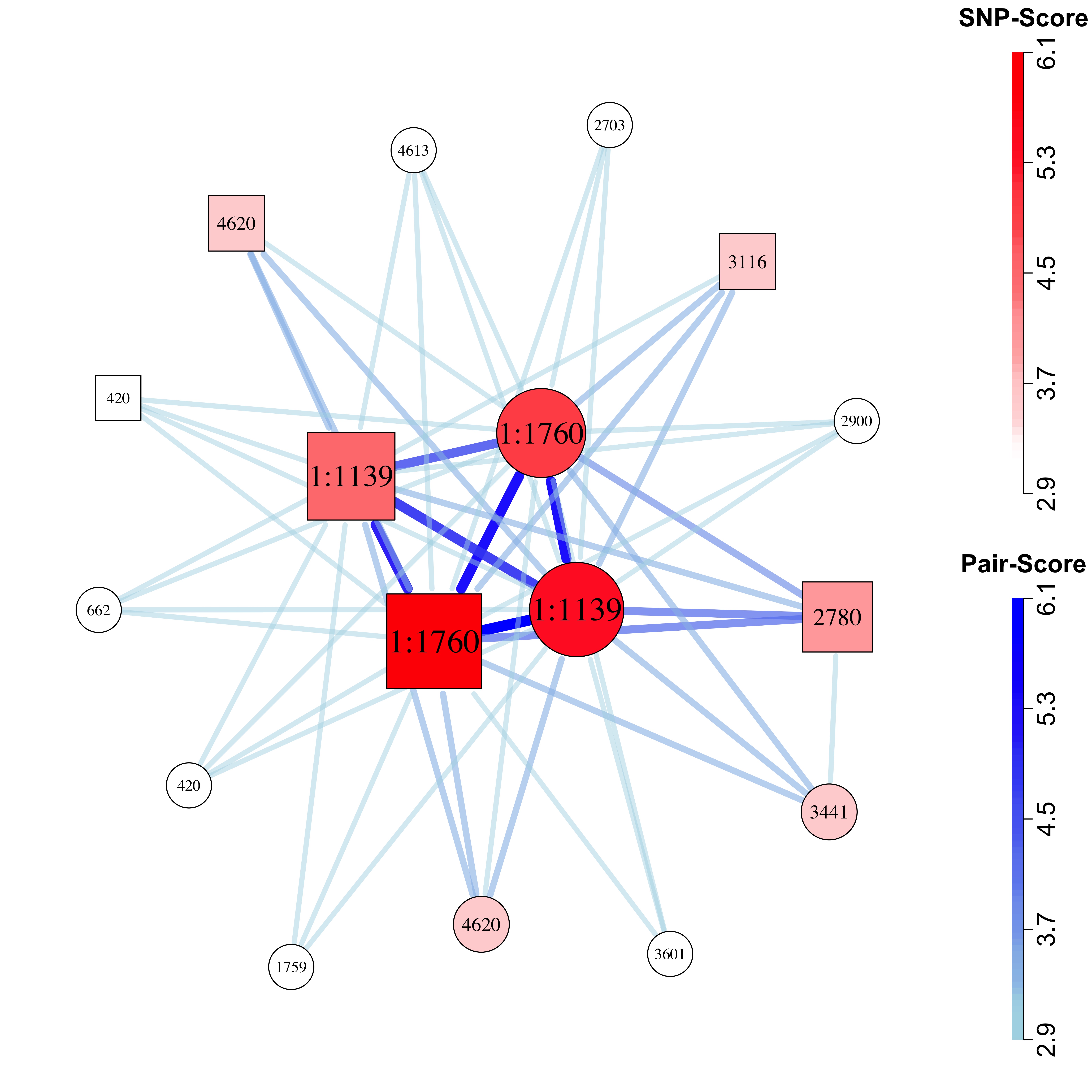


**Supplementary Figure S10. Network plot for simulation scenario 8, replicate 2.** Circles represent child SNPs and squares represent maternal SNPs. SNP label ‘1:’ indicates membership in the risk-related SNP-set with a maternal-fetal interaction effect. A SNP with no colon in the label is not-risk related. The number following the colon is the simulated SNP's identifier. Maternal and child SNPs with the same identifier represent the same locus. The SNP-sets that contributed to this plot were selected using the method described by Nodzenski *et al.*(Nodzenski et al., 2022) . After applying that filter, we plotted all 76 SNP-pairs (comprising 32 SNPs) that received graphical scores. Thicker, darker connections indicate higher SNP-pair graphical scores; larger, darker vertices indicate higher individual SNP graphical scores.


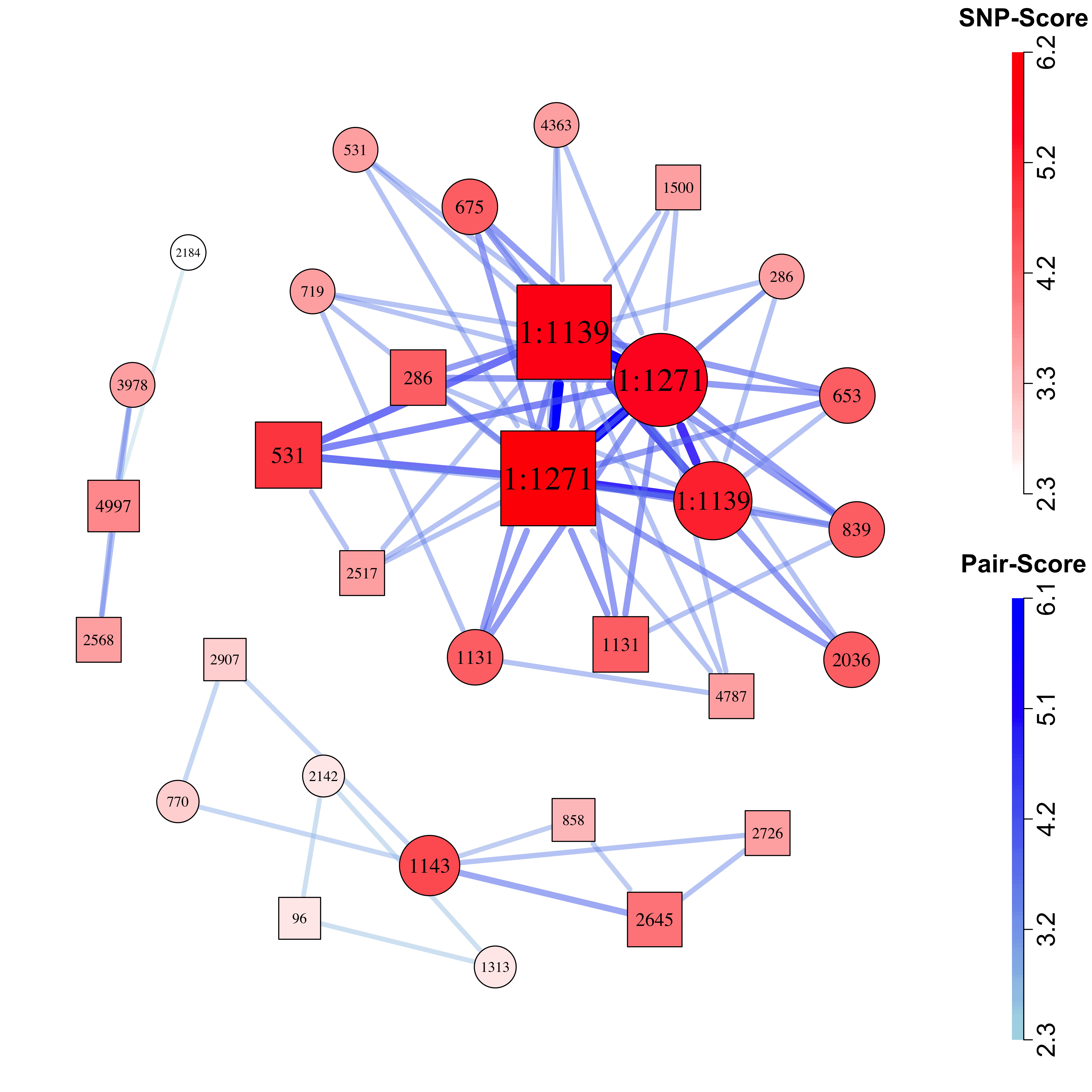


**Supplementary Figure S11. Network plot for simulation scenario 9, replicate 4.** Circles represent child SNPs and squares represent maternal SNPs. SNP label ‘1:’ indicates membership in the risk-related SNP-set with a maternal-fetal interaction effect. A SNP with no colon in the label is not-risk related. The number following the colon is the simulated SNP's identifier. Maternal and child SNPs with the same identifier represent the same locus. The SNP-sets that contributed to this plot were selected using the method described by Nodzenski *et al.*(Nodzenski et al., 2022) . After applying that filter, we plotted all 31 SNP-pairs (comprising 10 SNPs) that received graphical scores. Thicker, darker connections indicate higher SNP-pair graphical scores; larger, darker vertices indicate higher individual SNP graphical scores. Dashed connections indicate pairs of SNPs located on the same biological chromosome with pairwise R^2^ of at least 0.1 in controls (complement-siblings for child SNPs, fathers for maternal SNPs).


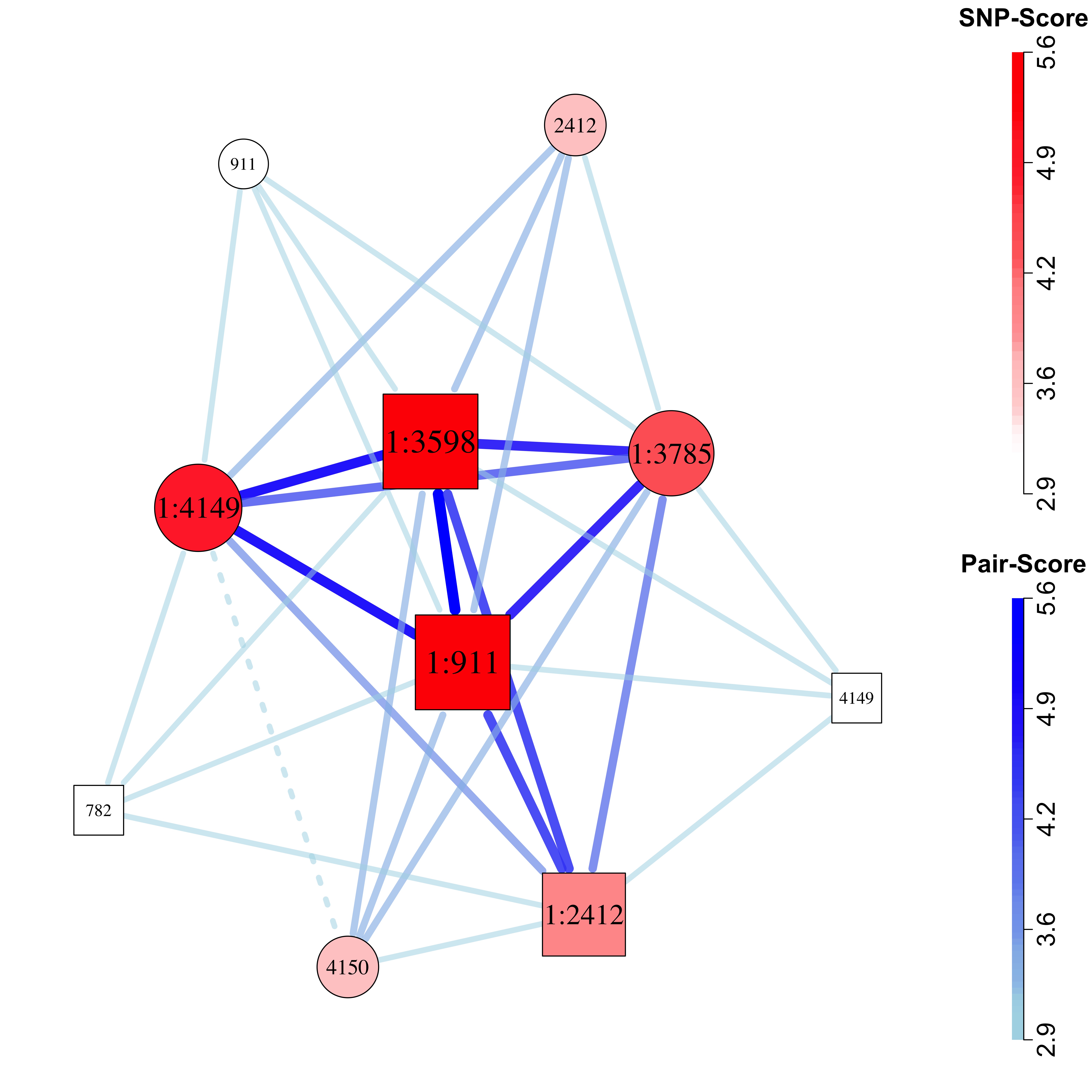


**Supplementary Figure S12. Network plot for simulation scenario 10, replicate 7.** Circles represent child SNPs and squares represent maternal SNPs. SNP label ‘1:’ indicates membership in the risk-related SNP-set with a maternal-fetal interaction effect. A SNP with no colon in the label is not-risk related. The number following the colon is the simulated SNP's identifier. Maternal and child SNPs with the same identifier represent the same locus. The SNP-sets that contributed to this plot were selected using the method described by Nodzenski *et al.*(Nodzenski et al., 2022) .After applying that filter, we plotted all 62 SNP-pairs (comprising 22 SNPs) that received graphical scores. Thicker, darker connections indicate higher SNP-pair graphical scores; larger, darker vertices indicate higher individual SNP graphical scores.


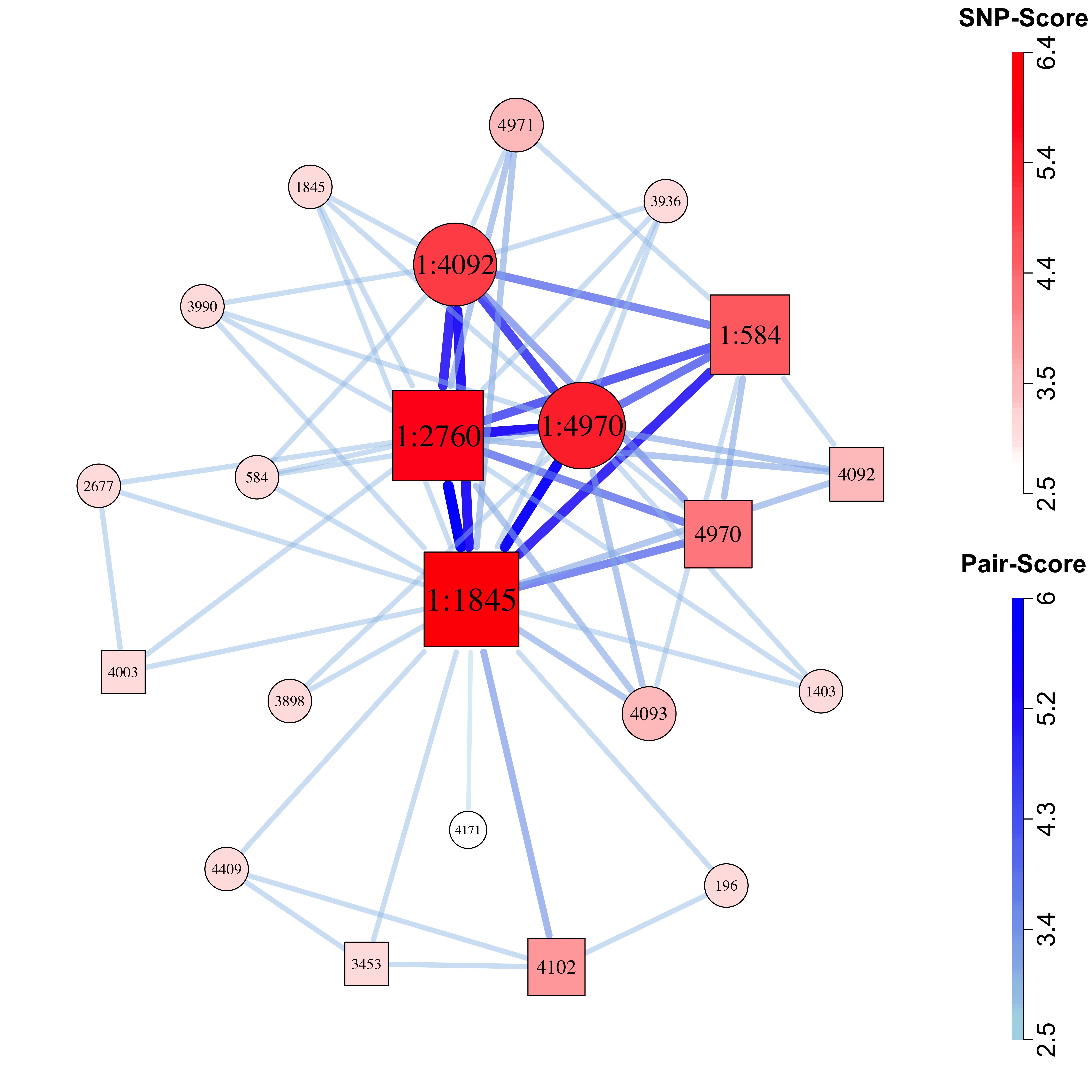


**Supplementary Figure S13. Network plot for simulation scenario 11, replicate 5.** Circles represent child SNPs and squares represent maternal SNPs. SNP label ‘1:’ indicates membership in the risk-related SNP-set with an epistatic maternally-mediated effect. A SNP with no colon in the label is not-risk related. The number following the colon is the simulated SNP's identifier. Parent and child SNPs with the same identifier represent the same locus. The SNP-sets that contributed to this plot were selected using the method described by Nodzenski *et al.*(Nodzenski et al., 2022) . After applying that filter, we plotted all 84 SNP-pairs (comprising 34 SNPs) that received graphical scores. Thicker, darker connections indicate higher SNP-pair graphical scores; larger, darker vertices indicate higher individual SNP graphical scores.


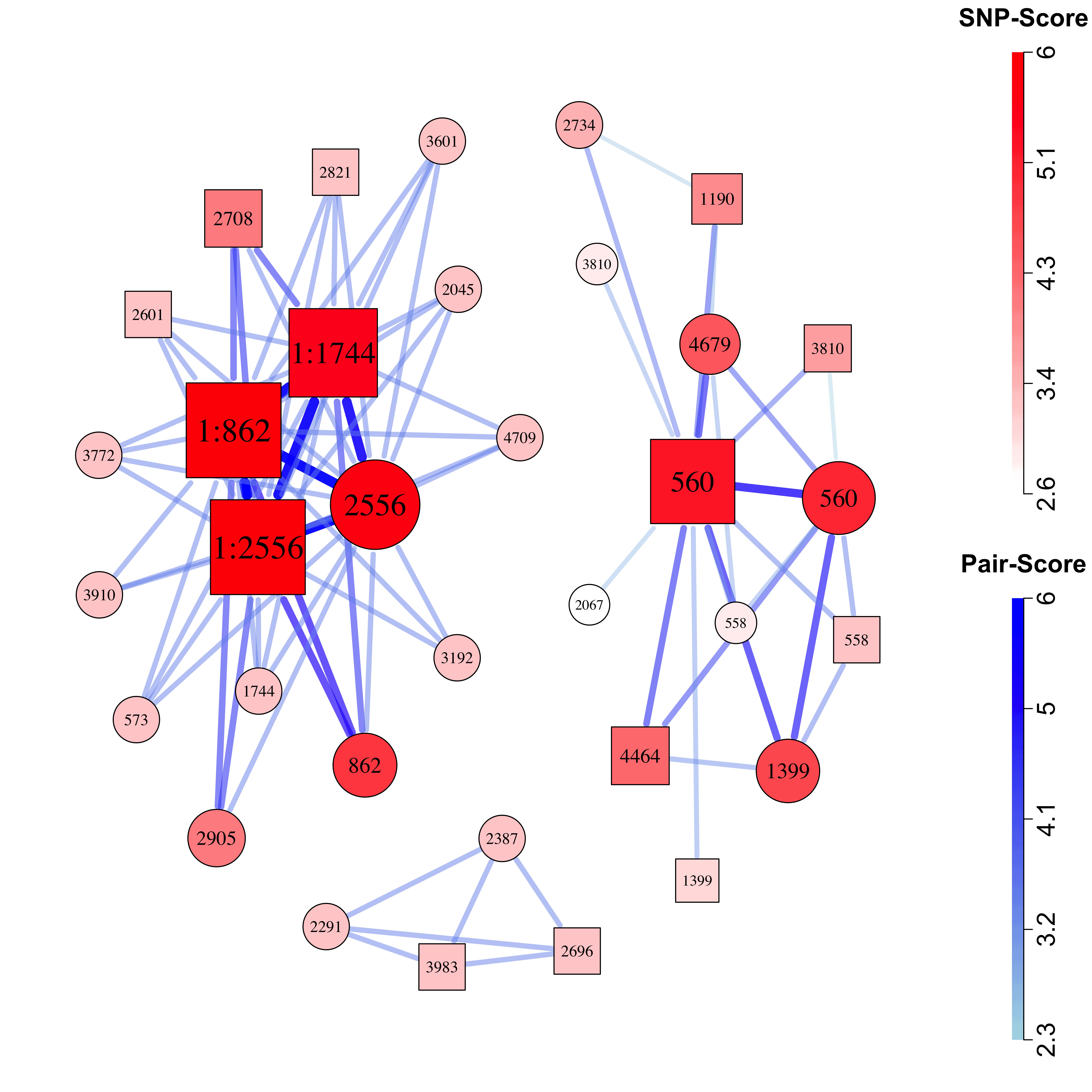


**Supplementary Figure S14. Network plot for simulation scenario 12, replicate 7.** Circles represent child SNPs and squares represent maternal SNPs. SNP label ‘1:’ indicates membership in the risk-related SNP-set with an epistatic maternally-mediated effect. A SNP with no colon in the label is not-risk related. The number following the colon is the simulated SNP's identifier. Maternal and child SNPs with the same identifier represent the same locus. The SNP-sets that contributed to this plot were selected using the method described by Nodzenski *et al.*(Nodzenski et al., 2022) . After applying that filter, we plotted all 80 SNP-pairs (comprising 37 SNPs) that received graphical scores. Thicker, darker connections indicate higher SNP-pair graphical scores; larger, darker vertices indicate higher individual SNP graphical scores.


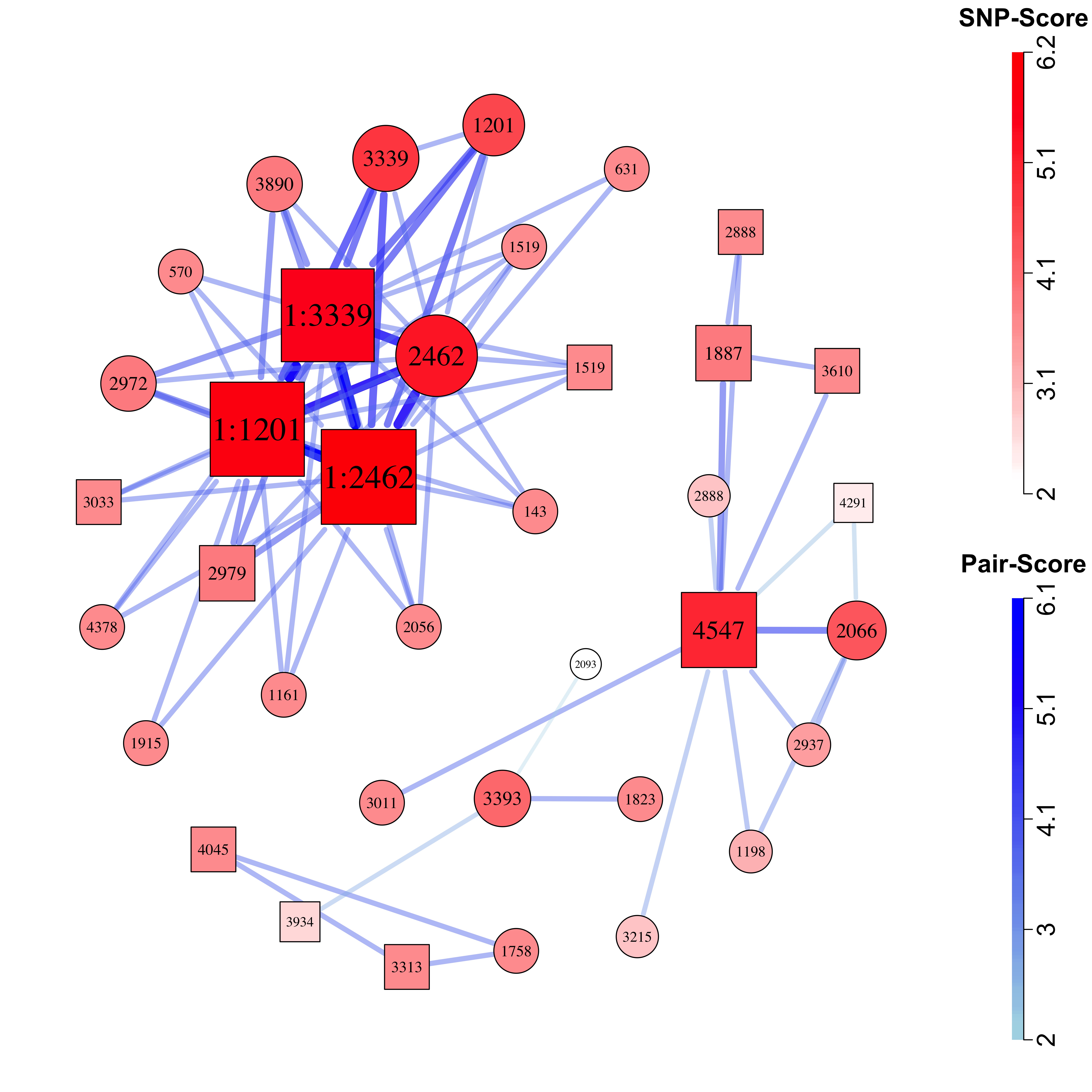


**Supplementary Figure S15. Network plot for simulation scenario 14, replicate 6.** Circles represent child SNPs and squares represent maternal SNPs. SNP labels ‘1:’ or ‘2:’ indicate membership in the first or second risk-related SNP-set, respectively, each with a simulated maternal-fetal interaction effect. The number following the colon is the simulated SNP's identifier. A SNP with no colon in the label is not-risk related. Mother and child SNPs with the same identifier represent the same locus. The SNP-sets that contributed to this plot were selected using the method described by Nodzenski *et al.*(Nodzenski et al., 2022) . After applying that filter, we plotted all 68 SNP-pairs (comprising 25 SNPs) that received graphical scores. Thicker, darker connections indicate higher SNP-pair graphical scores; larger, darker vertices indicate higher individual SNP graphical scores.


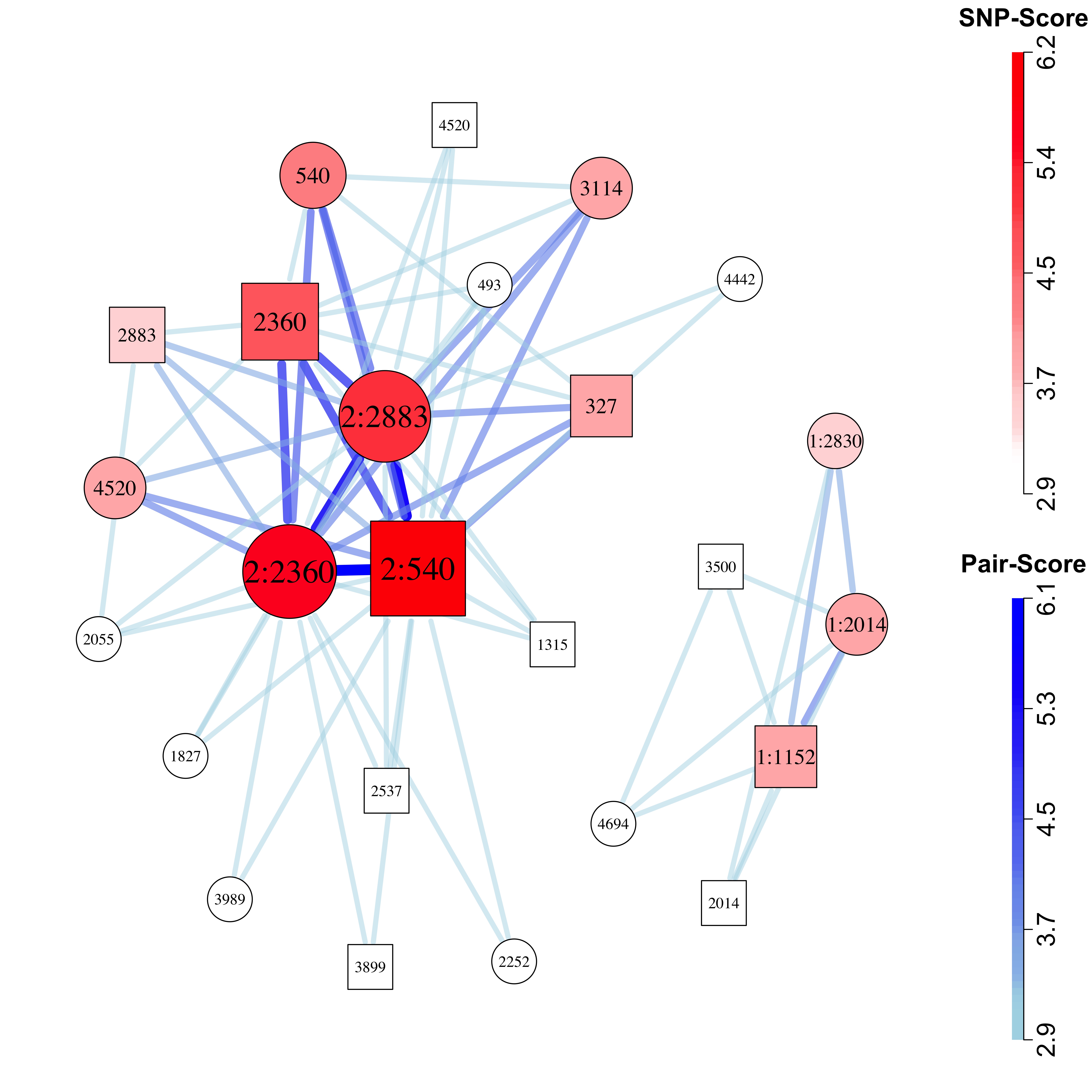


**Supplementary Figure S16. Network plot for simulation scenario 15, replicate 8.** Circles represent child SNPs and squares represent maternal SNPs. SNP labels ‘1:’ or ‘2:’ indicate membership in the first or second risk-related SNP-set, respectively, each with a simulated epistatic maternally-mediated effect. The number following the colon is the simulated SNP's identifier. A SNP with no colon in the label is not-risk related. Maternal and child SNPs with the same identifier represent the same locus. The SNP-sets that contributed to this plot were selected using the method described by Nodzenski *et al.*(Nodzenski et al., 2022) . After applying that filter, we plotted all 56 SNP-pairs (comprising 20 SNPs) that received graphical scores. Thicker, darker connections indicate higher SNP-pair graphical scores; larger, darker vertices indicate higher individual SNP graphical scores.


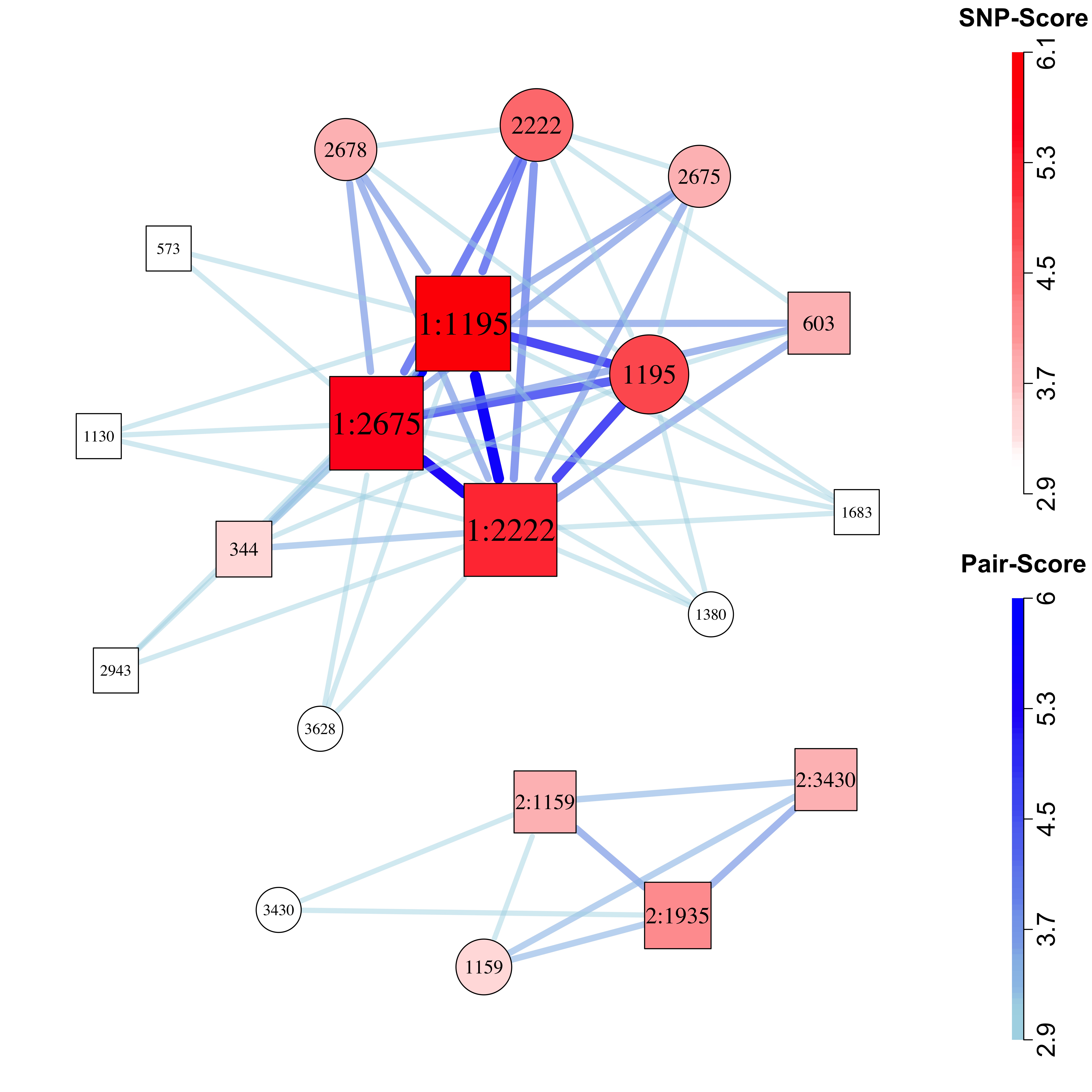


**Supplementary Figure S17. Network plot for simulation scenario 16, replicate 8.** Circles represent child SNPs and squares represent maternal SNPs. SNP labels ‘1:’ or ‘2:’ indicate membership in the first or second risk-related SNP-set, respectively, each with a simulated maternally-mediated genetic interaction effect. The number following the colon is the simulated SNP's identifier. A SNP with no colon in the label is not-risk related. Maternal and child SNPs with the same identifier represent the same locus. The SNP-sets that contributed to this plot were selected using the method described by Nodzenski *et al.*(Nodzenski et al., 2022) . After applying that filter, we plotted all 61 SNP-pairs (comprising 24 SNPs) that received graphical scores. Thicker, darker connections indicate higher SNP-pair graphical scores; larger, darker vertices indicate higher individual SNP graphical scores.


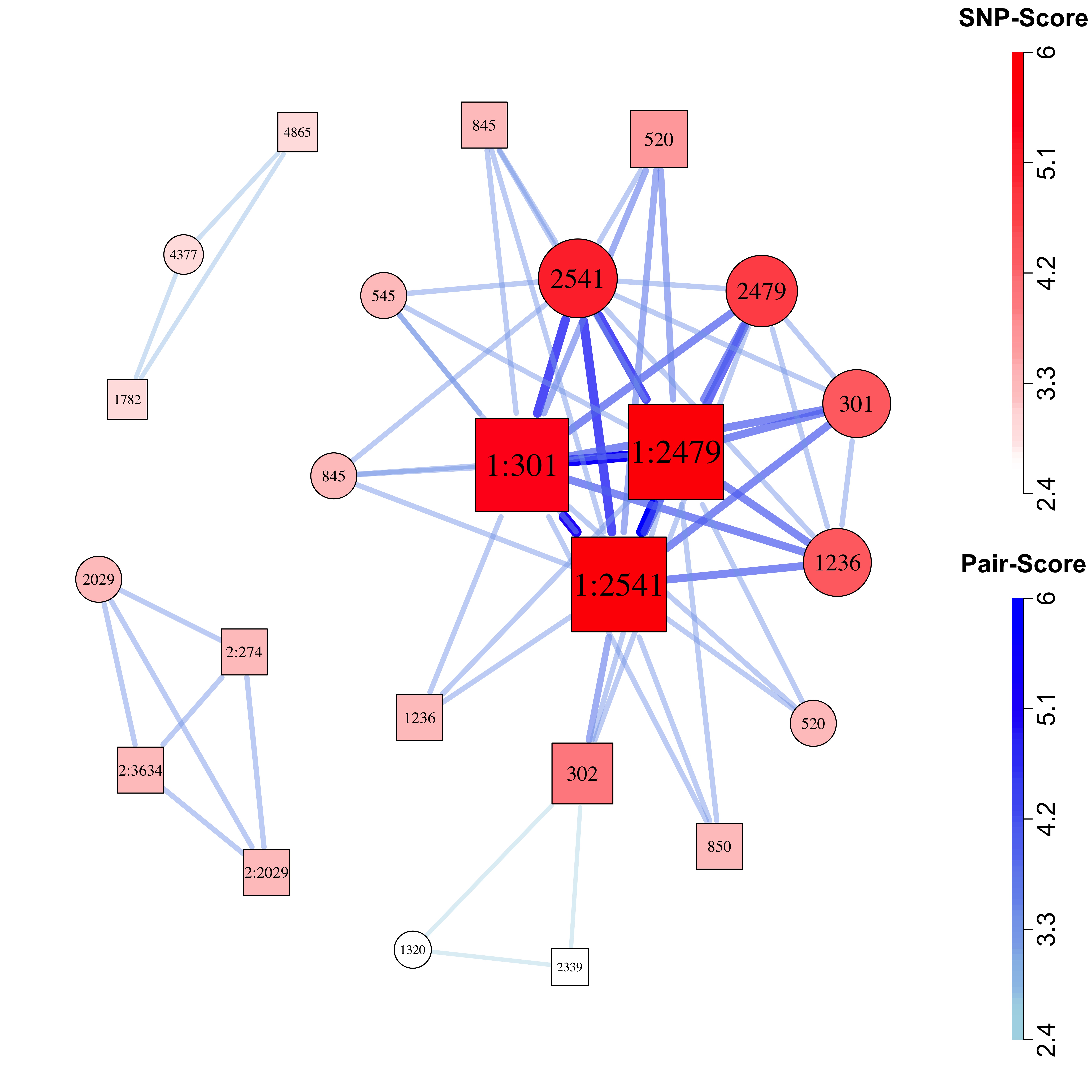


**Supplementary Figure S18. Network plot for simulation scenario 17, replicate 7.** Circles represent child SNPs and squares represent maternal SNPs. SNP labels ‘1:’ or ‘2:’ indicate membership in the first or second risk-related SNP-set, respectively. The first set has a simulated maternal-fetal interaction effect and the second has an epistatic maternally-mediated effect. The number following the colon is the simulated SNP's identifier. A SNP with no colon in the label is not-risk related. Maternal and child SNPs with the same identifier represent the same locus. The SNP-sets that contributed to this plot were selected using the method described by Nodzenski *et al.*(Nodzenski et al., 2022) . After applying that filter, we plotted all 60 SNP-pairs (comprising 23 SNPs) that received graphical scores. Thicker, darker connections indicate higher SNP-pair graphical scores; larger, darker vertices indicate higher individual SNP graphical scores.


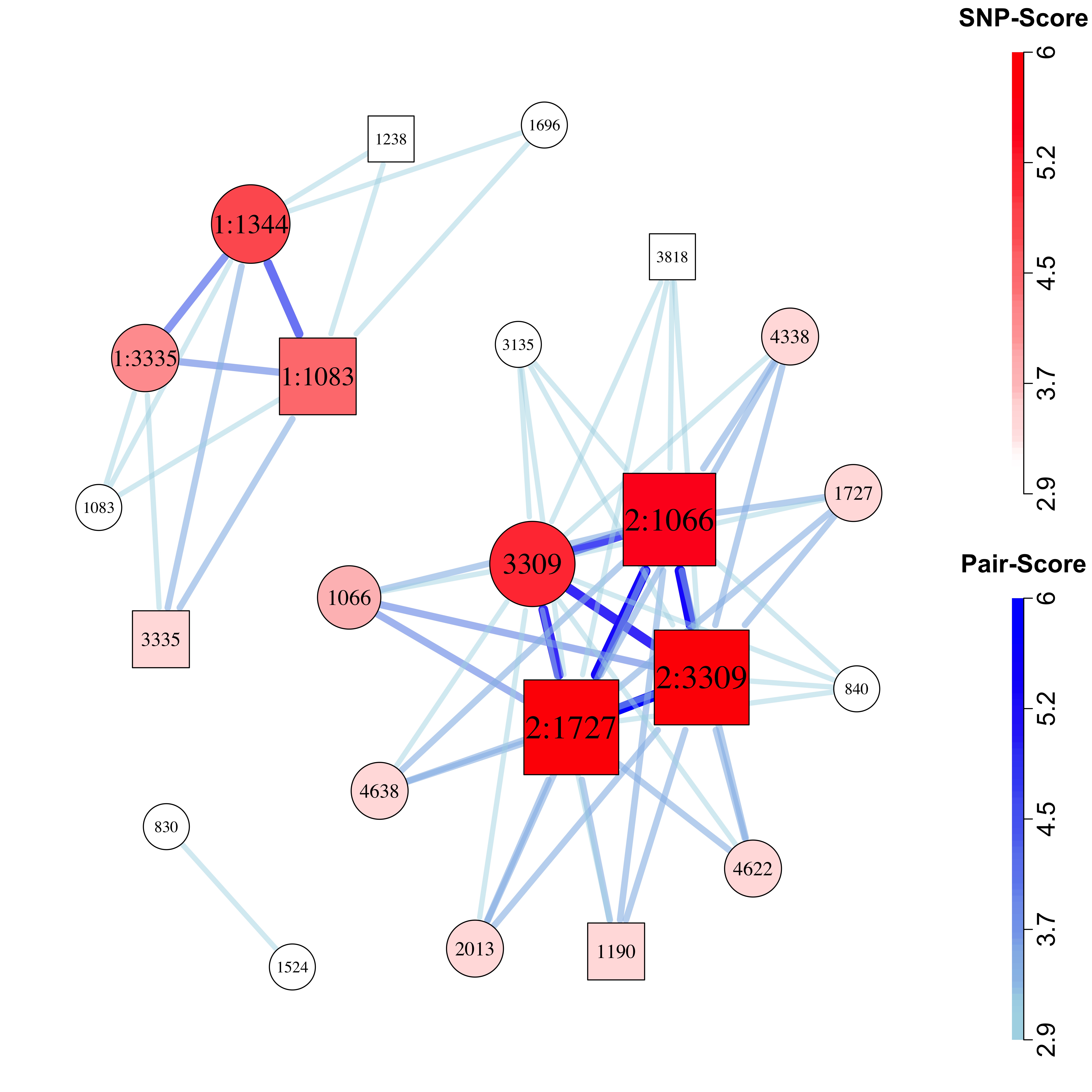


**Supplementary Figure S19. Network plot for simulation scenario 18, replicate 7.** Circles represent child SNPs and squares represent maternal SNPs. SNP labels ‘1:’ or ‘2:’ indicate membership in the first or second risk-related SNP-set, respectively. The first set has a simulated maternal-fetal interaction effect and the second has an epistatic child-SNP interaction. The number following the colon is the simulated SNP's identifier. A SNP with no colon in the label is not-risk related. Maternal and child SNPs with the same identifier represent the same locus. The SNP-sets that contributed to this plot were selected using the method described by Nodzenski *et al.*(Nodzenski et al., 2022) . After applying that filter, we plotted all 72 SNP-pairs (comprising 30 SNPs) that received graphical scores. Thicker, darker connections indicate higher SNP-pair graphical scores; larger, darker vertices indicate higher individual SNP graphical scores. Dashed connections indicate pairs of SNPs located on the same chromosome with pairwise R^2^ of at least 0.1 in controls (complement-siblings for child SNPs, fathers for maternal SNPs).


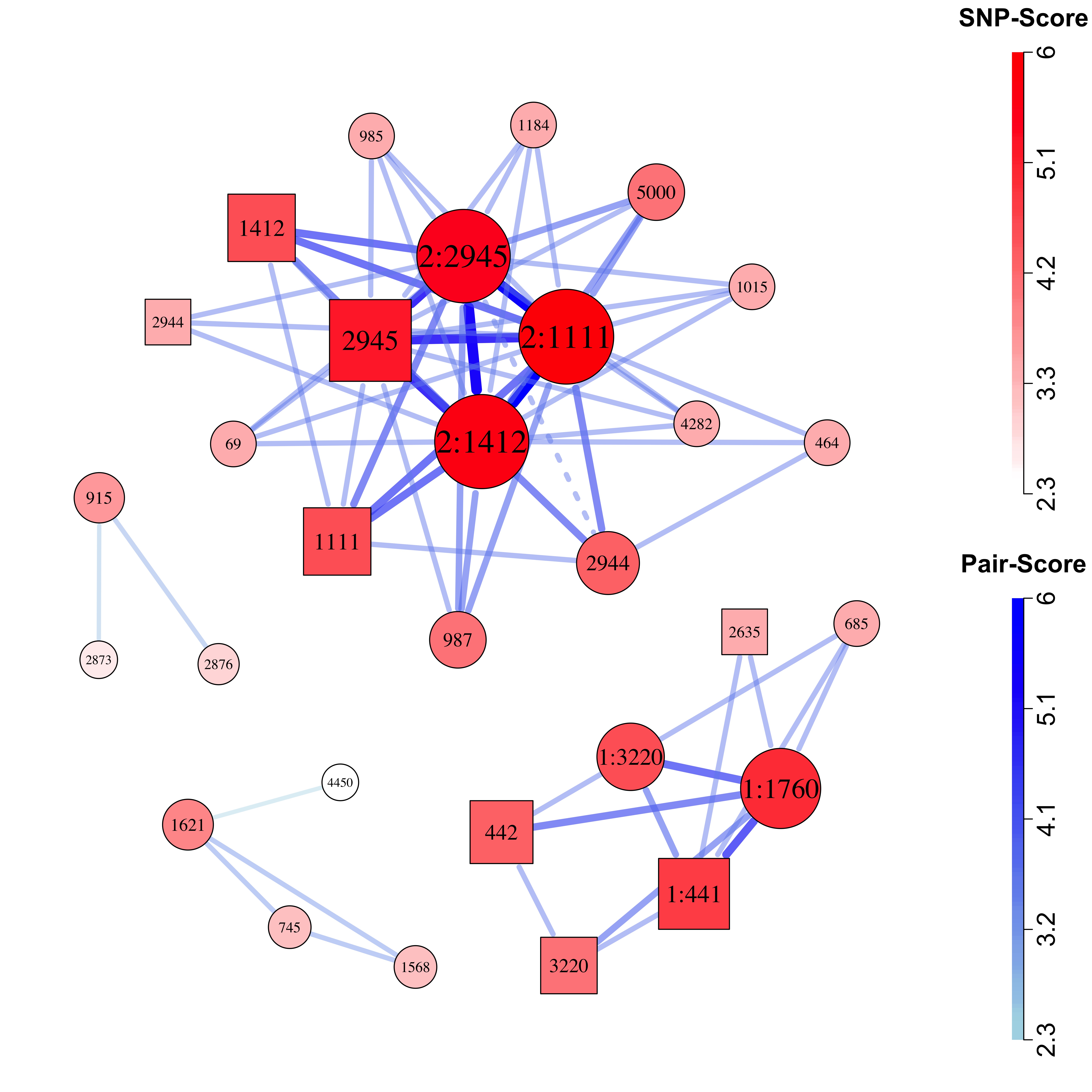


**Supplementary Figure S20. Network plot for simulation scenario 19, replicate 8.** Circles represent child SNPs and squares represent maternal SNPs. SNP labels ‘1:’ or ‘2:’ indicate membership in the first or second risk-related SNP-set, respectively. The first set has a simulated epistatic maternally-mediated interaction effect and the second has an epistatic child-SNP interaction. The number following the colon is the simulated SNP's identifier. A SNP with no colon in the label is not-risk related. Maternal and child SNPs with the same identifier represent the same locus. The SNP-sets that contributed to this plot were selected using the method described by Nodzenski *et al.*(Nodzenski et al., 2022) . After applying that filter, we plotted all 60 SNP-pairs (comprising 23 SNPs) that received graphical scores. Thicker, darker connections indicate higher SNP-pair graphical scores; larger, darker vertices indicate higher individual SNP graphical scores.


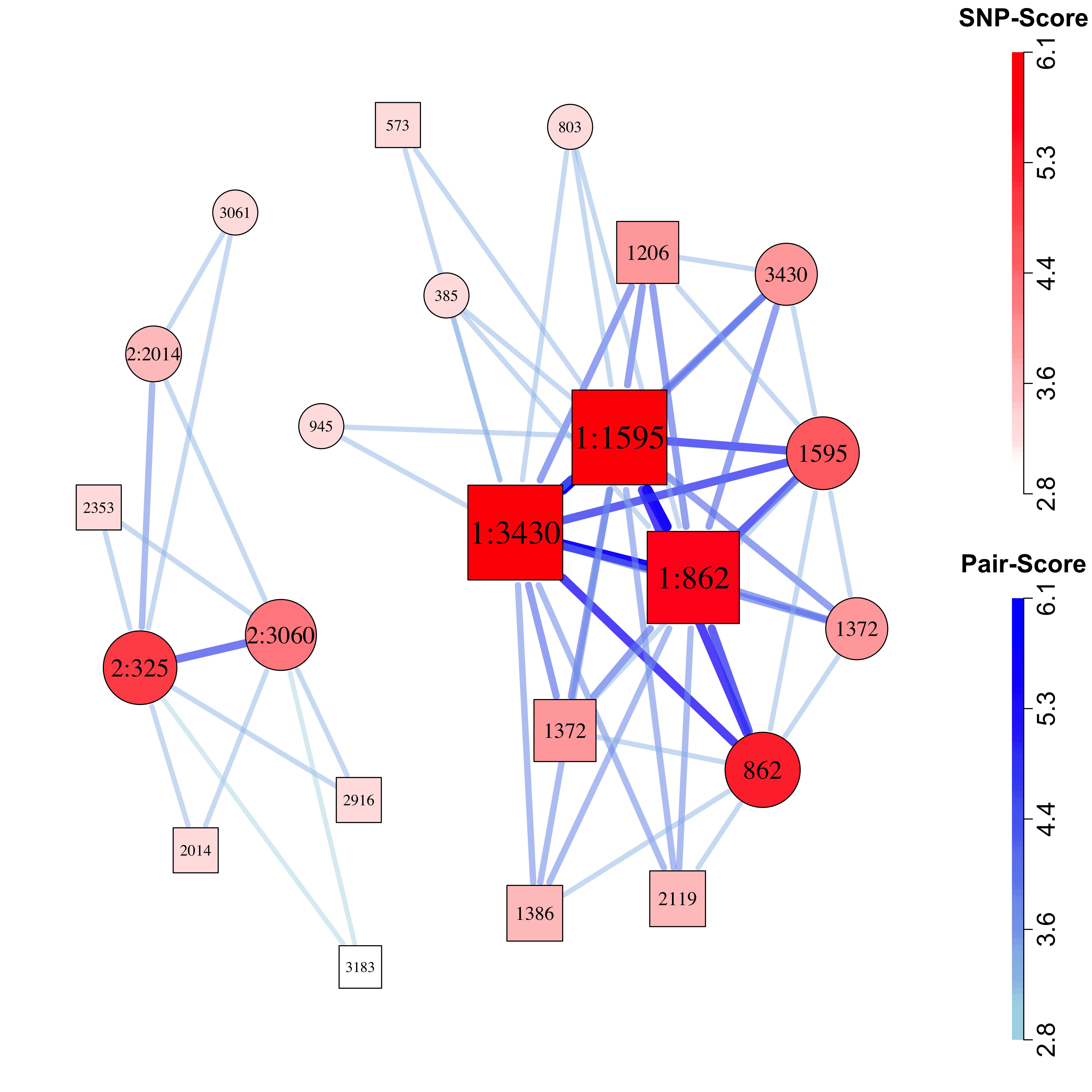


**Supplementary Figure S21. Network plot for simulation scenario 20, replicate 9.** Circles represent child SNPs and squares represent maternal SNPs. SNP labels ‘1:’ or ‘2:’ indicate membership in the first or second risk-related SNP-set, respectively, each with a simulated epistatic child-SNP effect. No SNPs from risk-related SNP-set 2 were identified. The number following the colon is the simulated SNP's identifier. A SNP with no colon in the label is not-risk related. Maternal and child SNPs with the same identifier represent the same locus. The SNP-sets that contributed to this plot were selected using the method described by Nodzenski *et al.*(Nodzenski et al., 2022) . After applying that filter, we plotted all 43 SNP-pairs (comprising 13 SNPs) that received graphical scores. Thicker, darker connections indicate higher SNP-pair graphical scores; larger, darker vertices indicate higher individual SNP graphical scores. Dashed connections indicate pairs of SNPs located on the same chromosome with pairwise R^2^ of at least 0.1 in controls (complement-siblings for child SNPs, fathers for maternal SNPs).


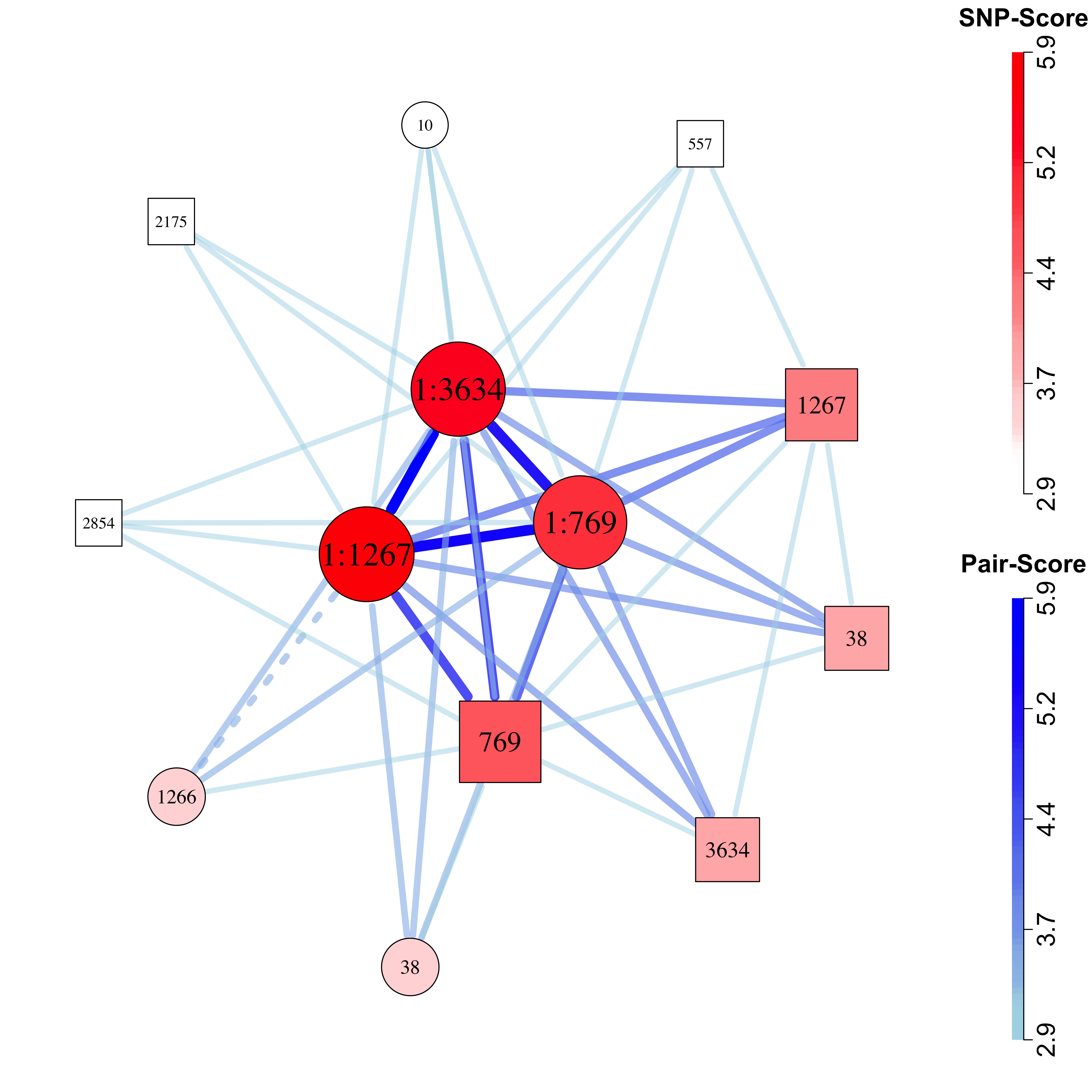


**Supplementary Figure S22. Network plot for simulation scenario 21, replicate 5.** Circles represent child SNPs and squares represent maternal SNPs. SNP label ‘S:’ indicates a singleton with a simulated non-epistatic SNP effect. The number following the colon is the simulated SNP's identifier. SNPs without an “S” are not risk-related. Maternal and child SNPs with the same identifier represent the same locus. This scenario had no epistasis and only multiplicative singleton effects (four child and four maternal SNPs). The SNP-sets that contributed to this plot were selected using the method described by Nodzenski *et al.*(Nodzenski et al., 2022) . After applying that filter, we plotted all 96 SNP-pairs (comprising 34 SNPs) that received graphical scores. Thicker, darker connections indicate higher SNP-pair graphical scores; larger, darker vertices indicate higher individual SNP graphical scores. Only three of the eight singleton SNPs affecting risk were found in this example, and a set of four falsely appears to be epistatic.


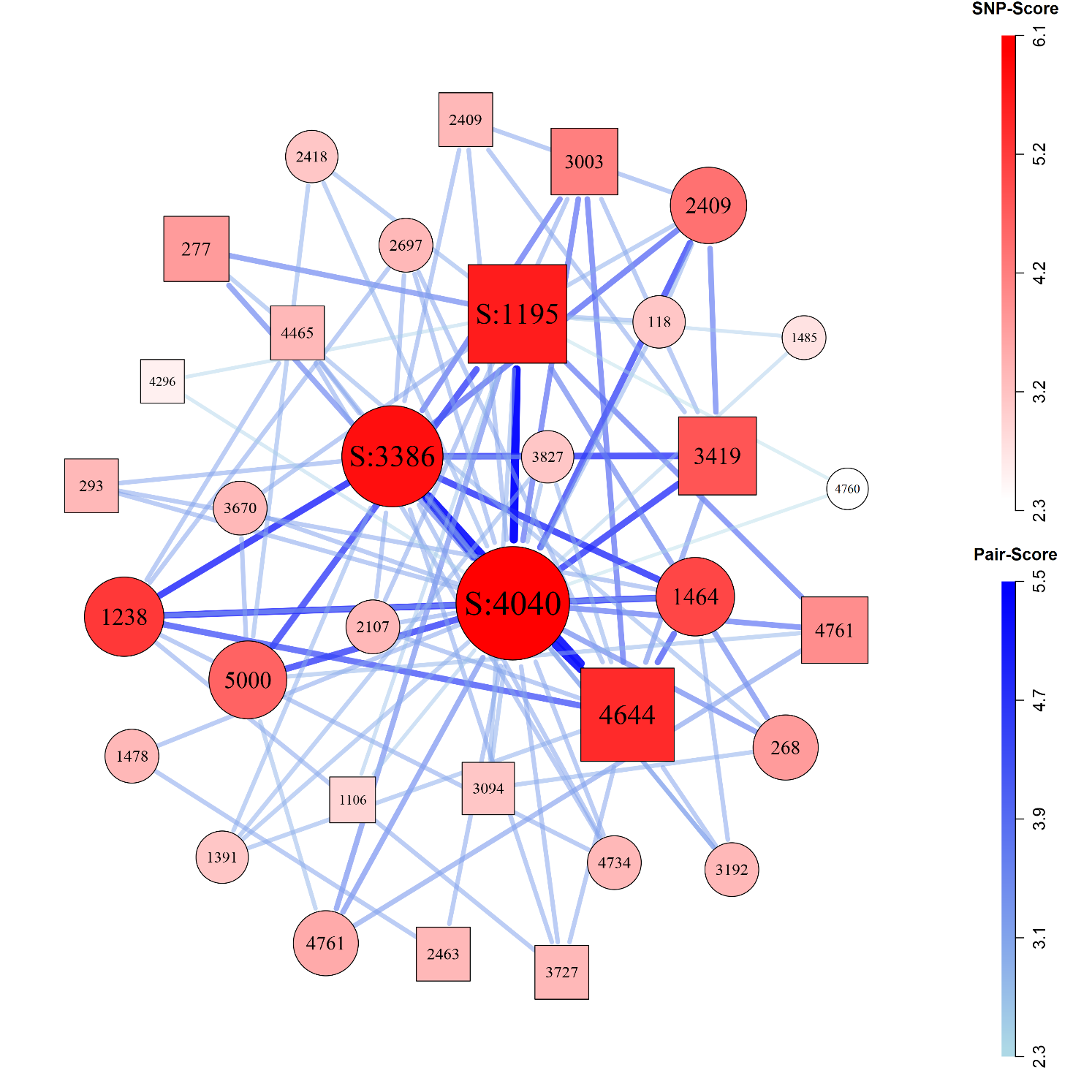


**Supplementary Figure S23. Network plot for simulation scenario 22, replicate 5.** Circles represent child SNPs and squares represent maternal SNPs. SNP labels ‘1:’ or ‘S:’ indicate membership in an epistatic or singleton SNP-set, respectively, each with a simulated risk effect. The number following the colon is the simulated SNP's identifier. A SNP with no colon in the label is not risk-related. Maternal and child SNPs with the same identifier represent the same locus. This scenario had one child three-SNP epistatic set and five singleton SNPs. The SNP-sets that contributed to this plot were selected using the method described by Nodzenski *et al.*(Nodzenski et al., 2022) . After applying that filter, we plotted all 67 SNP-pairs (comprising 26 SNPs) that received graphical scores. Thicker, darker connections indicate higher SNP-pair graphical scores; larger, darker vertices indicate higher individual SNP graphical scores.


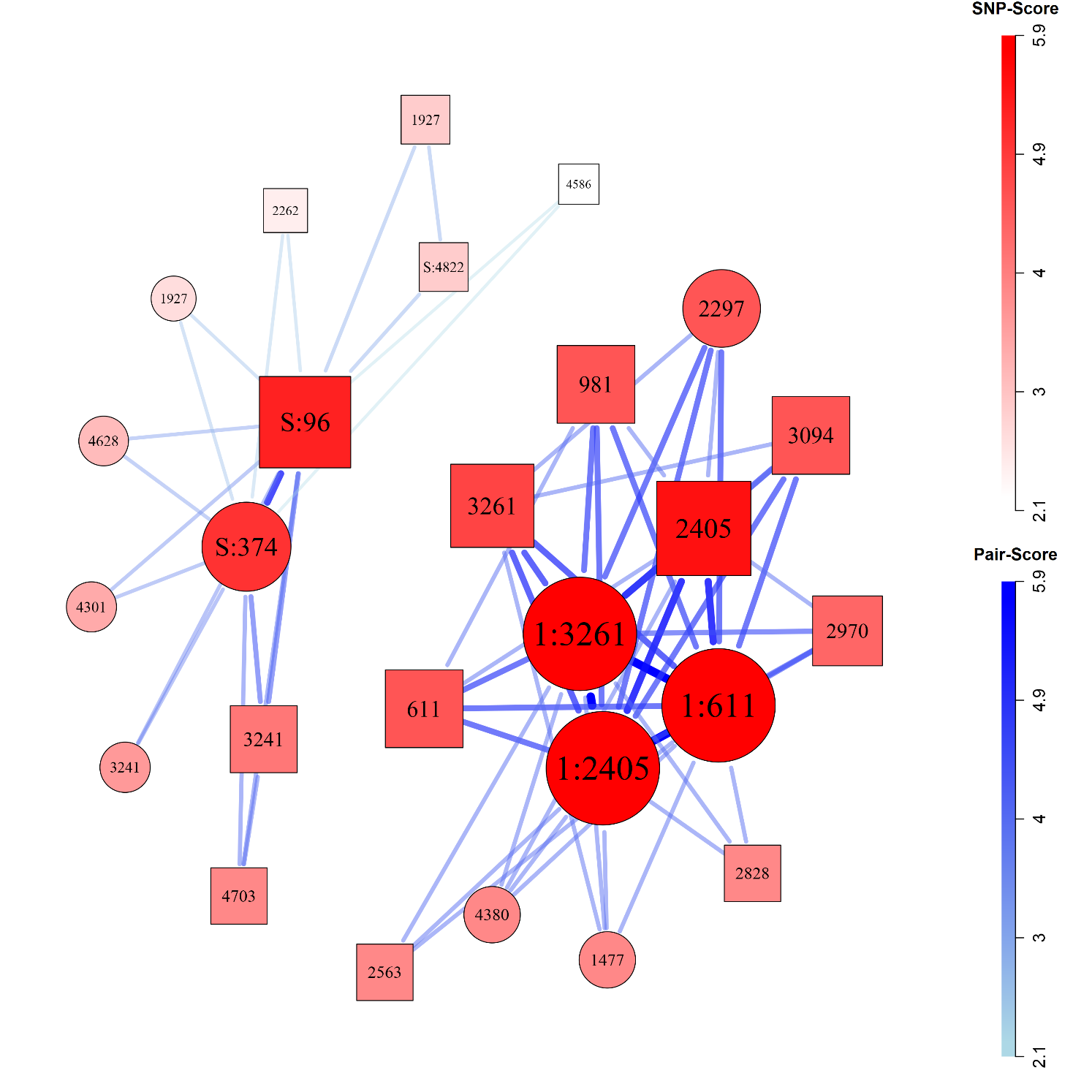


**Supplementary Figure S24. Network plot for simulation scenario 23, replicate 9.** Circles represent child SNPs and squares represent maternal SNPs. SNP labels ‘1:’ or ‘S:’ indicate membership in an epistatic or singleton SNP-set, respectively, each with a simulated risk effect. The number following the colon is the simulated SNP's identifier. A SNP with no colon in the label is not risk-related. Maternal and child SNPs with the same identifier represent the same locus. This scenario had one maternal three-SNP epistatic set and four singleton SNPs. The SNP-sets that contributed to this plot were selected using the method described by Nodzenski *et al.*(Nodzenski et al., 2022) . After applying that filter, we plotted all 90 SNP-pairs (comprising 36 SNPs) that received graphical scores. Thicker, darker connections indicate higher SNP-pair graphical scores; larger, darker vertices indicate higher individual SNP graphical scores. Dashed connections indicate pairs of SNPs located on the same chromosome with pairwise R^2^ of at least 0.1 in controls (complement-siblings for child SNPs, fathers for maternal SNPs).


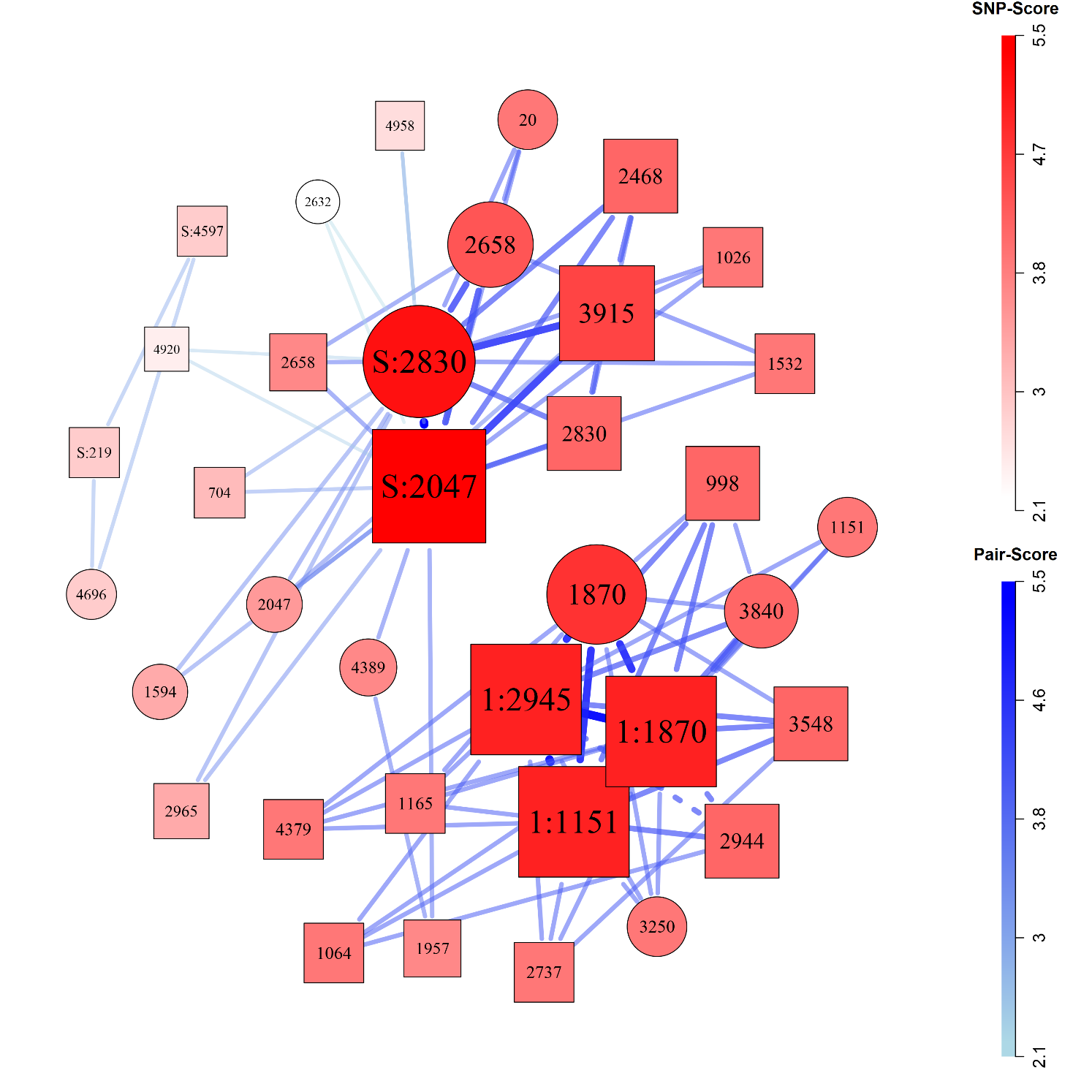


**Supplementary Figure S25. Network plot for simulation scenario 24, replicate 3.** Circles represent child SNPs and squares represent maternal SNPs. SNP labels ‘1:’ or ‘S:’ indicate membership in an epistatic or singleton risk-related SNP-set, respectively, each with a simulated epistatic effect. The number following the colon is the simulated SNP's identifier. A SNP with no colon in the label is not risk-related. Maternal and child SNPs with the same identifier represent the same locus. This scenario had one epistatic set containing four SNPs (two maternal and two child SNPs) and four singleton SNPs. The SNP-sets that contributed to this plot were selected using the method described by Nodzenski *et al.*(Nodzenski et al., 2022) . After applying that filter, we plotted all 97 SNP-pairs (comprising 37 SNPs) that received graphical scores. Thicker, darker connections indicate higher SNP-pair graphical scores; larger, darker vertices indicate higher individual SNP graphical scores.


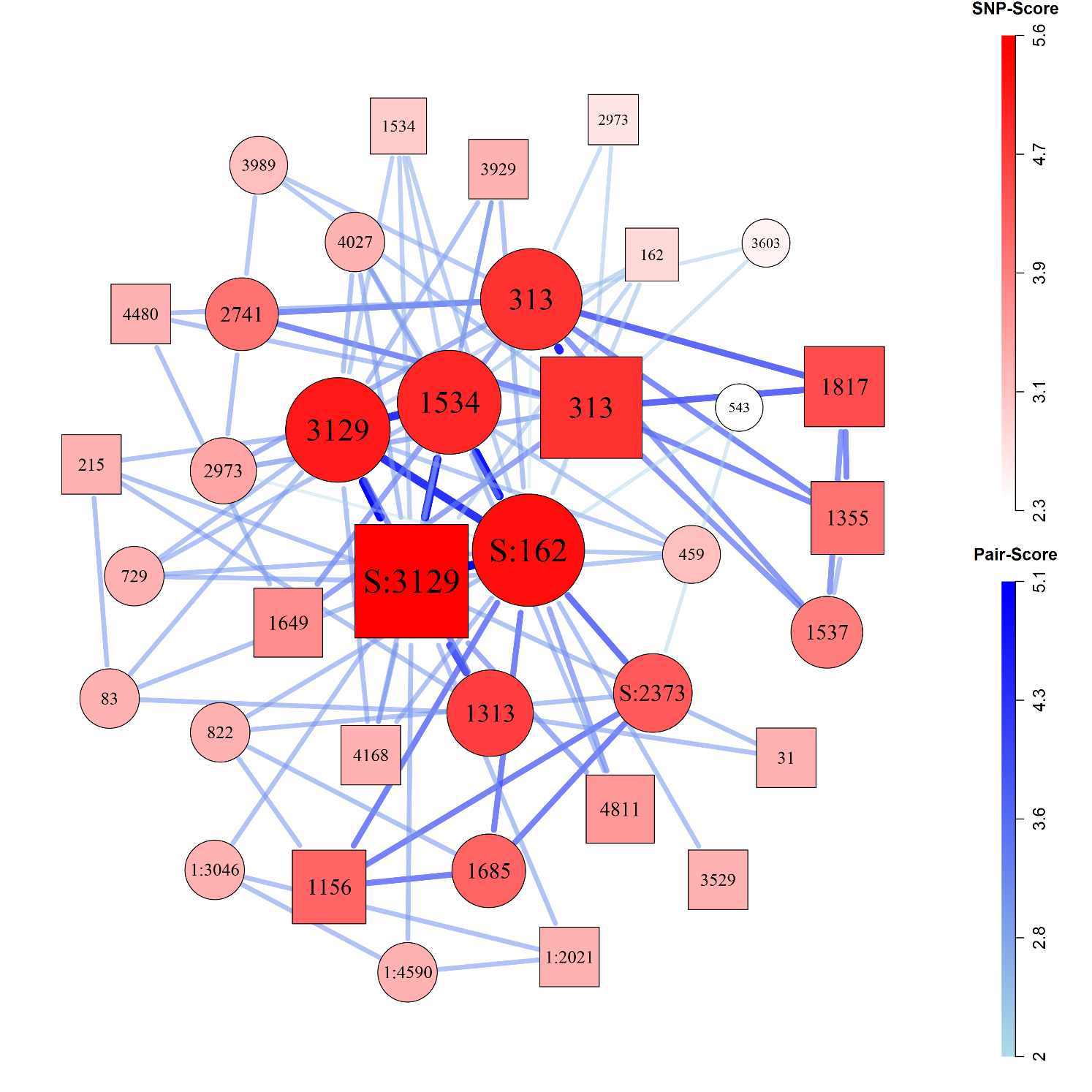

Supplement: Supplementary file 1 [file DataSheet1.docx]
